# Screening populations for copy number variation using genotyping-by-sequencing: a proof of concept using soybean fast neutron mutants

**DOI:** 10.1186/s12864-019-5998-1

**Published:** 2019-08-06

**Authors:** Marc-André Lemay, Davoud Torkamaneh, Guillem Rigaill, Brian Boyle, Adrian O. Stec, Robert M. Stupar, François Belzile

**Affiliations:** 1 Département de phytologie, Université Laval, Québec, QC Canada; 2 Institut de biologie intégrative et des systèmes, Université Laval, Québec, QC Canada; 3 Department of Plant Agriculture, University of Guelph, Guelph, ON Canada; 4 Institute of Plant Sciences Paris, Saclay (IPS2), CNRS, INRA, Université Paris-Sud, Université d’Evry, Université Paris-Saclay, Université Paris-Diderot, Sorbonne Paris-Cité, Paris, France; 5 LaMME, Université d’Evry Val d’Essonne, UMR CNRS 8071, ENSIIE, USC INRA, Paris, France; 6 Department of Agronomy and Plant Genetics, University of Minnesota, Saint Paul, MN USA

**Keywords:** Fast neutron-induced mutagenesis, Reverse genetic screen, Genotyping-by-sequencing, Comparative genomic hybridization, Copy number variation, Intra-cultivar heterogeneity, Functional genomics, Soybean genetics, Crop genetics, *Glycine max*

## Abstract

**Background:**

The effective use of mutant populations for reverse genetic screens relies on the population-wide characterization of the induced mutations. Genome- and population-wide characterization of the mutations found in fast neutron populations has been hindered, however, by the wide range of mutations generated and the lack of affordable technologies to detect DNA sequence changes. In this study, we therefore aimed to test whether genotyping-by-sequencing (GBS) technology could be used to characterize copy number variation (CNV) induced by fast neutrons in a soybean mutant population.

**Results:**

We called CNVs from GBS data in 79 soybean mutants and assessed the sensitivity and precision of this approach by validating our results against array comparative genomic hybridization (aCGH) data for 19 of these mutants as well as targeted PCR and ddPCR assays for a representative subset of the smallest events detected by GBS. Our GBS pipeline detected 55 of the 96 events found by aCGH, with approximate detection thresholds of 60 kb, 500 kb and 1 Mb for homozygous deletions, hemizygous deletions and duplications, respectively. Among the whole set of 79 mutants, the GBS data revealed 105 homozygous deletions, 32 hemizygous deletions and 19 duplications. This included several extremely large events, exhibiting maximum sizes of ~ 11.2 Mb for a homozygous deletion, ~ 11.6 Mb for a hemizygous deletion, and ~ 50 Mb for a duplication.

**Conclusions:**

This study provides a proof of concept that GBS can be used as an affordable high-throughput method for assessing CNVs in fast neutron mutants. The modularity of this GBS approach allows combining as many different libraries or sequencing runs as is necessary for reaching the goals of a particular study. This method should enable the low-cost genome-wide characterization of hundreds to thousands of individuals in fast neutron mutant populations or any population with large genomic deletions and duplications.

**Electronic supplementary material:**

The online version of this article (10.1186/s12864-019-5998-1) contains supplementary material, which is available to authorized users.

## Background

The use of mutants for elucidating gene function has had a long and successful history, having led to some of the most important breakthroughs in genetics over the last century (e.g. [1–4]). Forward genetic screens typically involve mutagenizing organisms through physical, chemical or bio-engineered mutagens and then screening the resulting mutants for atypical phenotypes of interest [5]. Reverse genetic screens, on the other hand, aim to first identify the individuals in a mutagenized population that harbor mutation(s) within a specific gene or group of genes at the DNA level, then study the resulting phenotype [5]. One such mutagen, fast neutron irradiation, has been used to generate mutant populations of several plant species including thale cress (*Arabidopsis thaliana*) [6, 7], rice (*Oryza sativa*) [6, 8], barrel medic (*Medicago truncatula*) [9, 10], birdsfoot trefoil (*Lotus japonicus*) [11], common bean (*Phaseolus vulgaris*) [12] and soybean (*Glycine max*) [13]. Fast neutron irradiation has been popular in plant functional genomics due to its ability to induce a wide array of mutations, ranging from single nucleotide polymorphisms (SNPs) and small indels (e.g. [7]) to large chromosomal disruptions spanning several megabases (e.g. [14, 15]).

Using fast neutron mutants for reverse genetic screens has proved challenging due to the lack of affordable technologies to comprehensively characterize the mutations found in a population. Early approaches used PCR-based methods to screen mutant populations for mutations in genes of interest [6, 9, 16], but these methods only targeted mid-sized deletions (from a few kb up to a dozen kb) and did not provide a genome-wide assessment of the mutations. More recently, array comparative genomic hybridization (aCGH) [13, 14] and whole-genome resequencing (WGS) [8, 15] have been applied to fast neutron populations in order to provide a genome-wide picture of the mutations in a subset of a population. However, with the exception of a study by Li et al. [15], aCGH or WGS can only realistically be performed on a relatively small number of individuals bearing the phenotype of interest (forward screen) or comprising a subset of an entire population, as these approaches are generally too costly for assessing fast neutron-induced mutations on a population scale (usually several thousands of individuals), especially in species with large genomes.

Bolon et al. [13] have developed a soybean fast neutron population which has been successfully used in forward genetic screens to identify genes associated with trichome integrity [17] and seed sucrose and oil content [18] as well genomic regions associated with seed composition and petiole length [14] through a combination of aCGH and WGS. These studies have found a high frequency of very large (up to several Mb) copy number variants (CNVs) in this population. Providing a population-wide assessment of these CNVs would prove beneficial in harnessing this mutant resource for reverse genetic screens; however, many individuals of this population remain uncharacterized due to the lack of an affordable and efficient high-throughput method for assessing them.

Genotyping-by-sequencing (GBS) may represent such a high-throughput method for genome-wide and population-scale assessment of CNVs in fast neutron mutants. Developed by Elshire et al. [19], GBS is one of several methods that use restriction enzymes in order to reduce the complexity of genomes prior to sequencing, enabling the simultaneous discovery and genotyping of thousands of SNPs and small indels in hundreds or thousands of individuals [20, 21]. GBS has been successfully applied for genotyping purposes in a myriad of plant species, including soybean [22], barley [23], wheat [24], and maize [25]. GBS has also recently been used to detect SNPs and small indels in a proton beam mutant population of soybean [26]. However, we are not aware of attempts to bring GBS beyond SNP and indel genotyping other than a study which assigned ploidy levels based on GBS data [27] and another which called CNVs from GBS data but did not provide independent validation of these calls [28].

In this study, we demonstrate that GBS can be used as a high-throughput and low-cost method for characterizing CNVs in a fast neutron-mutagenized population, and thus provide a more affordable approach for population-scale CNV discovery than aCGH or WGS. To do so, we subjected 92 mutants to a standard GBS protocol, 19 of which were also assessed by aCGH in order to provide a validation dataset for benchmarking the GBS pipeline. In addition to aCGH data, we validated some of the smallest CNVs detected by the GBS approach using targeted PCR and droplet digital PCR (ddPCR) assays. Our objectives as part of this study were to 1) develop and optimize an approach for CNV discovery from standard GBS data, 2) characterize the advantages and limits of the GBS approach relative to aCGH, and 3) describe the variation uncovered by GBS among the set of mutants.

## Results

### Development and validation of an approach to call copy number variants using GBS data

We wanted to see whether a GBS protocol could be used to call CNVs in a collection of 92 soybean mutants generated by irradiation with fast neutrons. Briefly, in this approach, the number of GBS reads falling within a 1-kb window in a given line was compared to the mean number observed across all lines within this same window (see Methods for details). The ratio between these two numbers was used to identify under- or over-represented genomic regions in much the same way as aCGH establishes a ratio between the hybridization signals seen in a test line and a control. Our approach for calling CNVs from the first GBS library relied on 32,741 1-kb informative bins in the dataset used for calling homozygous deletions and duplications, and 28,439 bins in the dataset used to call hemizygous deletions (Table 1, Additional file 1: Figure S1). To assess the accuracy and sensitivity of the CNVs called using this approach, we subjected 19 of these lines to aCGH on a 940 K array. We viewed the aCGH data as being reflective of the CNVs present within these lines and tried to optimize our GBS approach so that it would maximize the number of true CNVs retrieved while minimizing the number of false positives.

**Table 1:** Breakdown of the bin filtering steps of read count datasets

	Homozygous deletions and duplications	Hemizygous deletions
Mapping quality threshold	20	35
Initial number of reads	82,923,013^a^	75,841,175
Number of bins with at least 1 read	152,061	141,068
Bins removed by max-read filter	7	6
Bins removed by min-read filter	117,406^b^	111,039^b^
Bins removed by var./mean filter	1907	1584
Number of bins remaining	32,741	28,439
Number of reads in remaining bins	67,905,271	61,254,871

Numbers shown in this table pertain to the sequencing data obtained from the first GBS library. The read and bin counts shown come from 81 mutants and 4 controls. The same dataset was used to call homozygous deletions and duplications. Different filters were applied to the dataset used to call hemizygous deletions

^a^See Additional file 1: Figure S10 for the distribution of the number of mapped reads per individual

^b^Minimum number of reads/bin = 7 for homozygous deletions/duplications and = 8 for hemizygous deletions

**Fig. 1:**
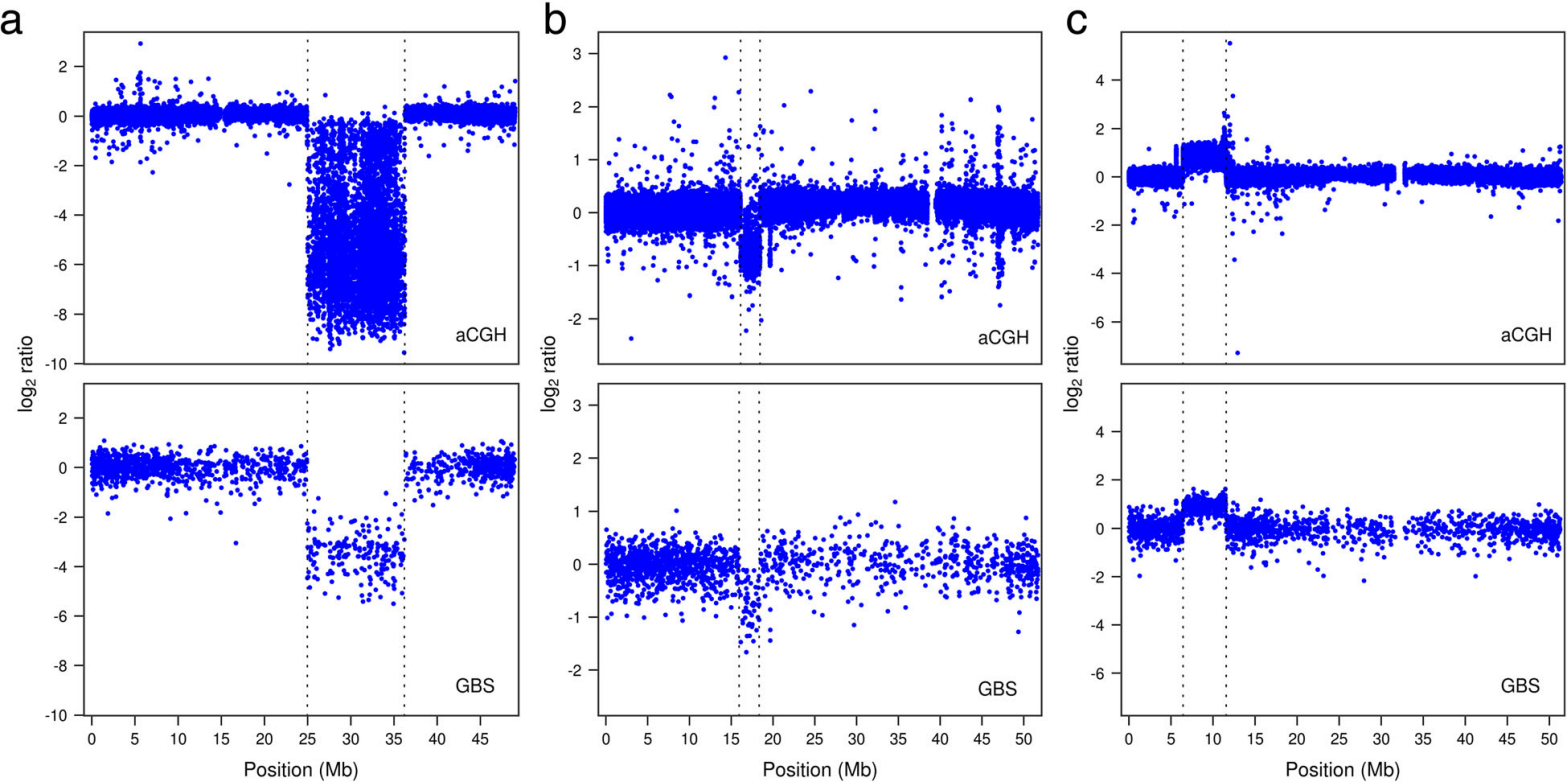
Examples of aCGH and GBS log_2_ ratio profiles of events detected using both methods. Vertical dotted lines mark event boundaries. The GBS log_2_ ratio data are those generated from the dataset used to called homozygous deletions and duplications from the first GBS library in all three cases. **a** log_2_ ratio profiles of a 11.2-Mb homozygous deletion on chromosome 14 of individual FNMN0065. **b** log_2_ ratio profiles of a 2.3-Mb hemizygous deletion on chromosome 15 of individual FNMN0039. **c** log_2_ ratio profiles of a 5.1-Mb duplication on chromosome 6 of FNMN0085. aCGH data also revealed a 50-kb duplication at around 5.6 Mb on this same chromosome; its visual signature can be seen on the GBS profile although it was not called by the automated pipeline

The aCGH analysis revealed the existence of 54 homozygous deletions, 22 hemizygous deletions and 20 duplications among 18 of the 19 lines tested (Additional file 2), one of the lines (FNMN0016) showing no evidence of CNV. Within this same set, our optimized GBS-based approach detected 29 homozygous deletions, 13 hemizygous deletions and 10 duplications, indicating that slightly more than half of the events called by aCGH could be found by GBS. The visual signature of the log_2_ ratios of events found by GBS were analogous to the ones observed from aCGH data (Fig. 1). Analysis of the set of GBS-derived CNVs revealed only one false positive hemizygous deletion, while no homozygous deletions or duplications were incorrectly called. In the course of the development of this approach, we found that a set of more relaxed parameters could detect up to 31 homozygous deletions and 14 hemizygous deletions from the first GBS library, but such a reduced stringency also resulted in several false positive calls (see Additional file 1: Figures S2 and S3).

In a few of the detected cases, GBS failed to correctly identify the structure of large or particularly complex events. This was the case for a set of four neighboring duplications on chromosome 1 of individual FNMN0077, which was captured as a single event by GBS (Additional file 1: Figure S4). Two other duplications in the aCGH dataset, including an almost complete whole-chromosome duplication, were split into two in the GBS set of calls. Attempts at finding a set of parameters that minimized these minor inconsistencies proved unsuccessful (see Additional file 1). Similarly, both the homozygous and hemizygous sets of deletions found by GBS each showed one occurrence of an aCGH deletion split into two, and one occurrence of two aCGH deletions called as a single deletion by the GBS approach.

**Fig. 2:**
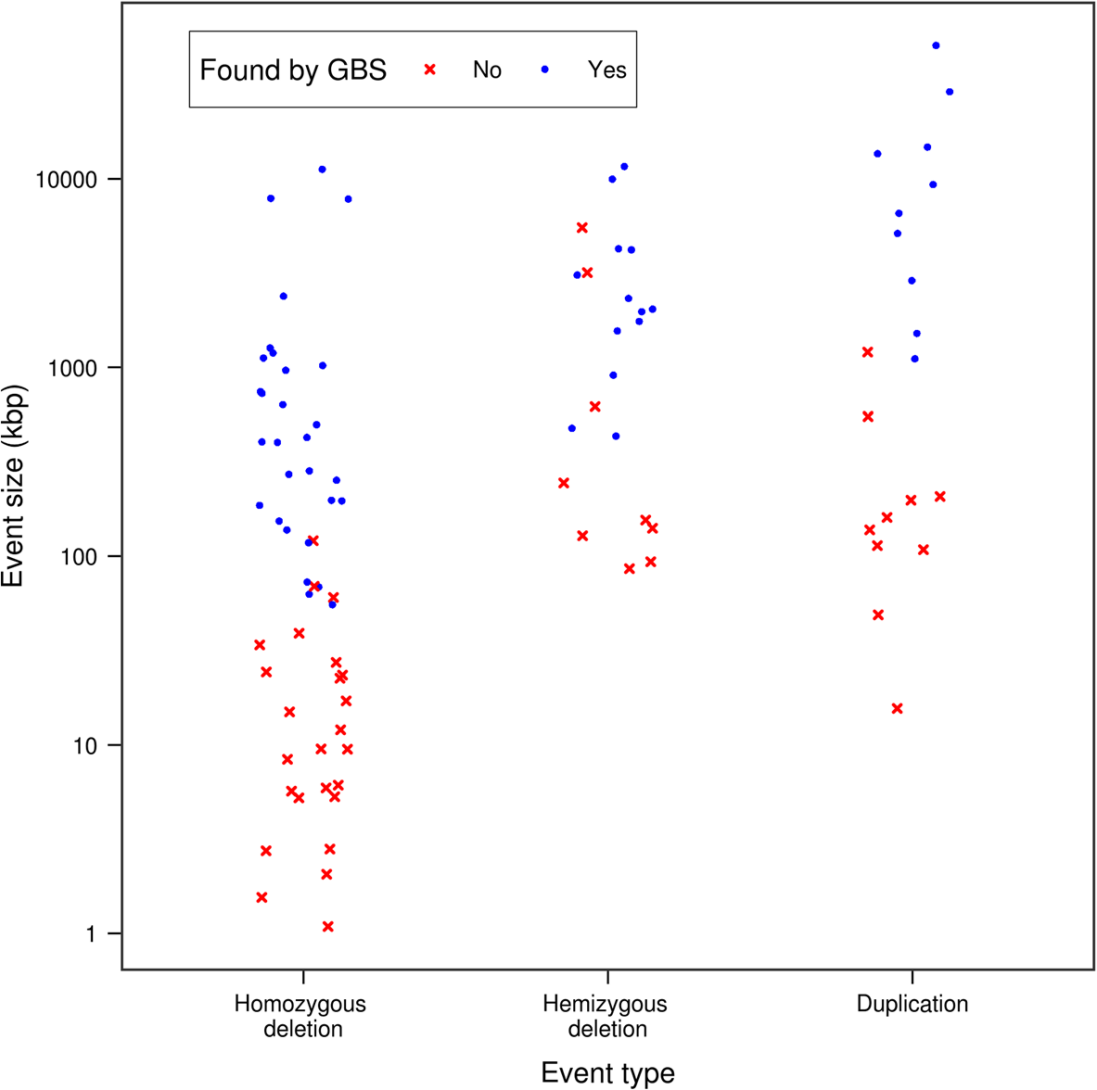
Size distribution of CNVs detected based on the aCGH data according to whether (blue dots) or not (red crosses) the GBS protocol and pipeline for calling CNVs detected them. The sizes plotted are those determined from the aCGH data. Points were jittered along the x-axis to avoid overlap between events of similar sizes. Note that the scale of the y-axis is logarithmic

Whether an event was detected by GBS was largely dependent on its size. To illustrate this, we labelled each of the CNVs identified using aCGH as having been successfully uncovered or not using GBS data and plotted their size distributions (Fig. 2). Except for one 120-kb deletion, GBS was able to detect all homozygous deletions larger than 70 kb, in addition to a few smaller deletions. The less extreme log_2_ ratio values generated by events such as duplications or hemizygous deletions put a greater constraint as to the minimal size of the events detectable by GBS, but this was also true of the aCGH approach (Fig. 2). Overall, the sizes above which events were likely to be detected were approximately 70 kb, 500 kb and 1 Mb for homozygous deletions, hemizygous deletions and duplications, respectively.

**Fig. 3:**
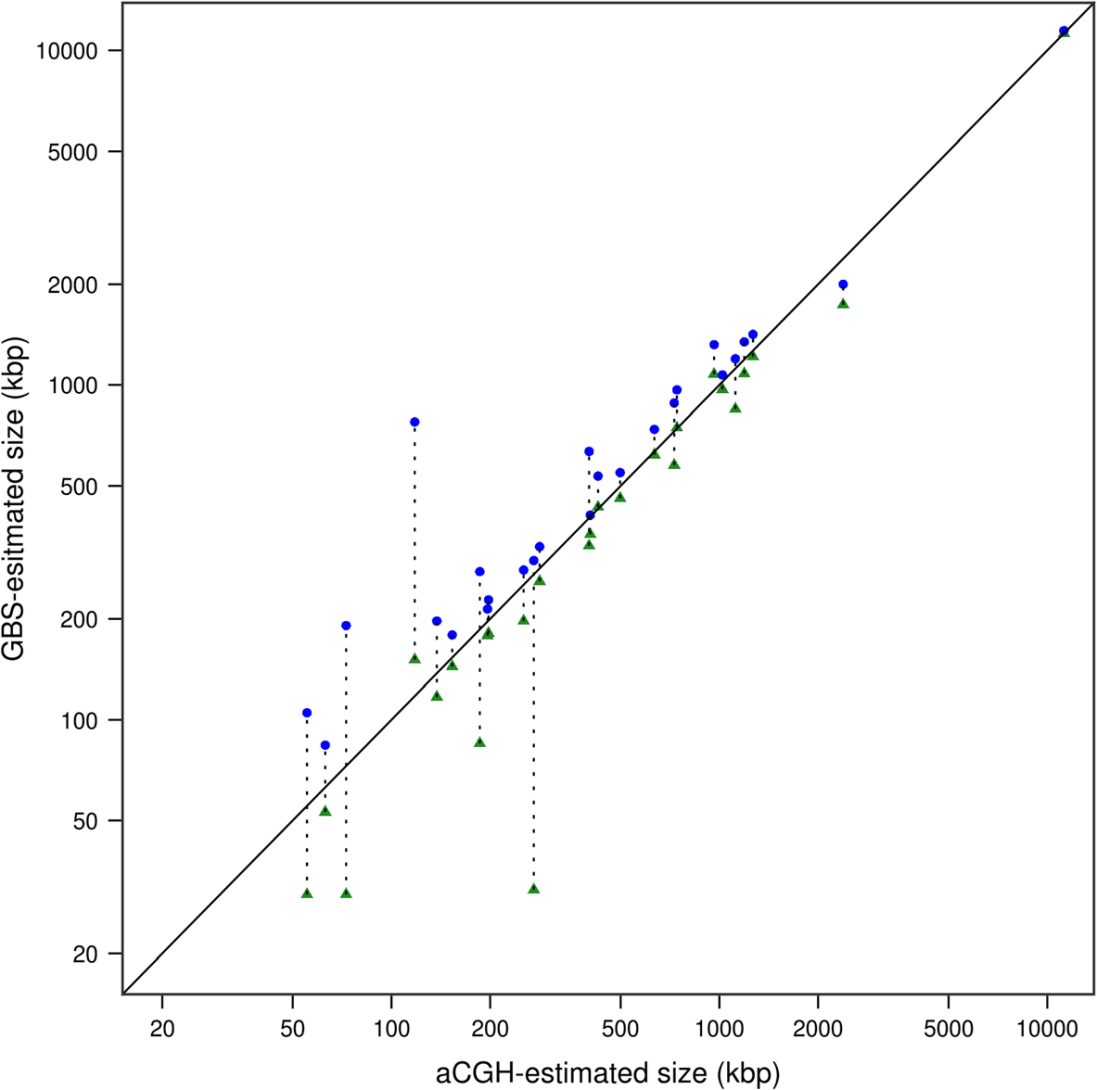
Comparison of the sizes of homozygous deletions as estimated from aCGH and GBS approaches. For a given GBS deletion, the minimum deletion span (green triangles) and the maximum deletion span (blue dots) are linked by a dotted line. The solid diagonal line marks the equality line on which all points should ideally lie. Note that the scales of both axes are logarithmic

To explore whether the size of homozygous deletions was accurately inferred from the GBS data, we calculated a maximum and minimum span for each deletion based on the positions of the GBS bins defining its boundaries. As expected, the size of the deletions estimated on the basis of the aCGH data generally fell between the minimum and maximum deletion spans as estimated from GBS data (Fig. 3). Most deletions identified by GBS defined a narrow range around the size estimated by aCGH; the minimum and maximum deletion span corresponded on average to 84 and 148% of the aCGH-estimated size, respectively. A simple linear model aiming at predicting the (log_10_-transformed) aCGH deletion size as a function of the (also log_10_-transformed) GBS-estimated minimum and maximum deletion spans had a very high predictive capability (adjusted R^2^ = 0.93). These results are based on the 26 homozygous deletions for which there was a one-to-one correspondence between the aCGH- and GBS-derived deletions (i.e. split and merged deletions were ignored).

### CNV events detected from the first GBS library among a set of 79 soybean mutants

Although the GBS library originally comprised 96 lines (92 mutants + 4 controls), a SNP catalogue produced using the GBS data indicated that 11 of the lines were not pure. Given this contamination, we chose to eliminate these lines from our efforts to call CNVs. After running our optimized CNV-calling pipeline on the GBS data, we also noticed a suspiciously high number of CNVs (30 and 11, respectively) in two of the individuals (FNMN0018 and FNMN0006), and the log_2_ ratio profiles of these individuals indeed suggested that these calls were spurious. Therefore, in total, we called 97 homozygous deletions, 30 hemizygous deletions, and 17 duplications among a final set of 79 mutants. Surprisingly, two additional deletions were found in each of two replicates of the control (M92–220) line. Given this surprising result, we chose to validate these two deletions by PCR and ddPCR assays, one of which proved to be true whereas inconclusive results were obtained for the other (more details below).

**Fig. 4:**
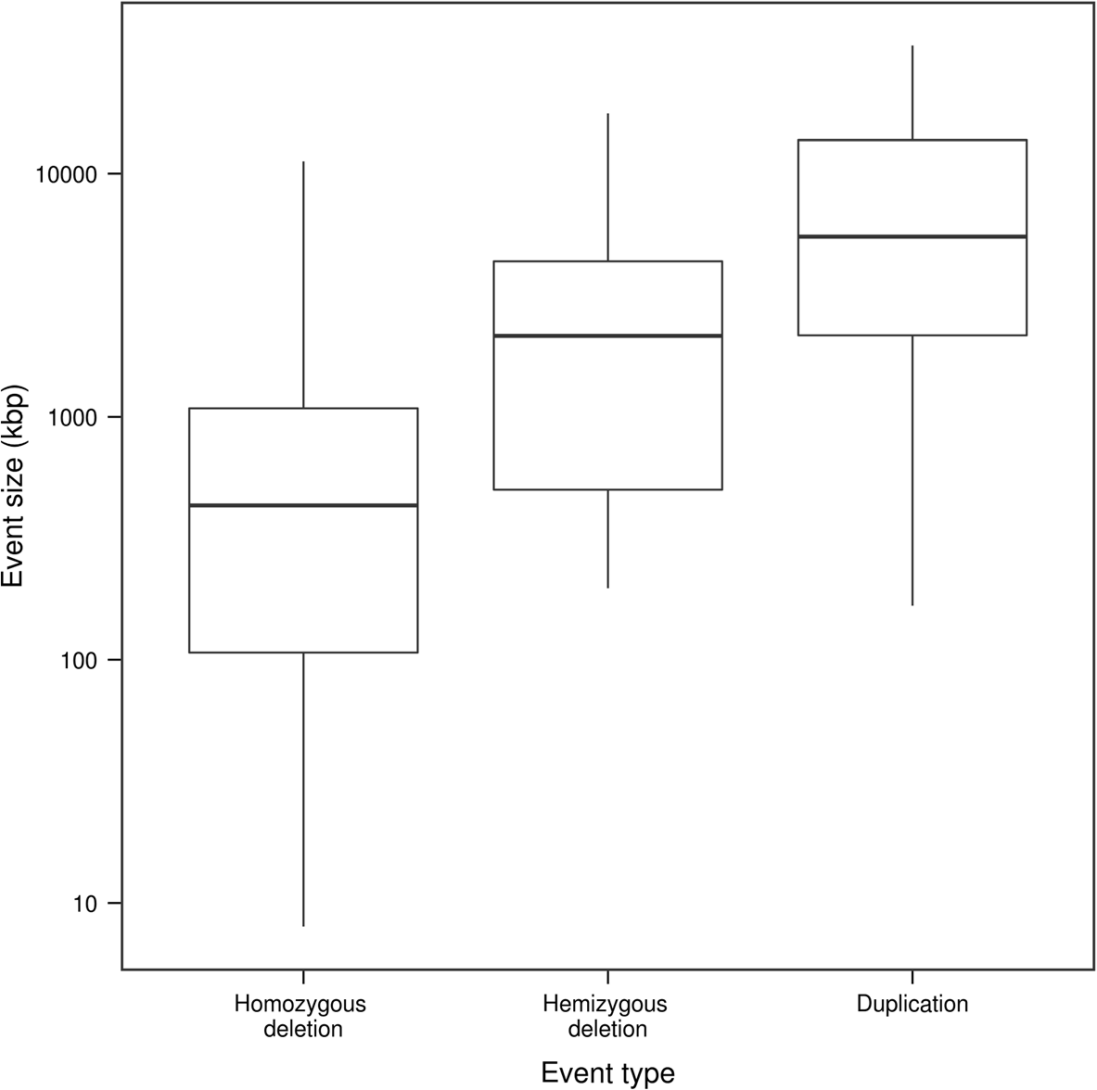
Size distribution of the events called from the data of the first GBS library among 79 fast neutron-mutagenized soybean lines. The event size plotted is the minimum span as estimated from the CNV-calling pipeline and is therefore likely an underestimation of the true event size. *n* = 97 for homozygous deletions, *n* = 30 for hemizygous deletions, and *n* = 17 for duplications. Note that the scale of the y-axis is logarithmic

**Fig. 5:**
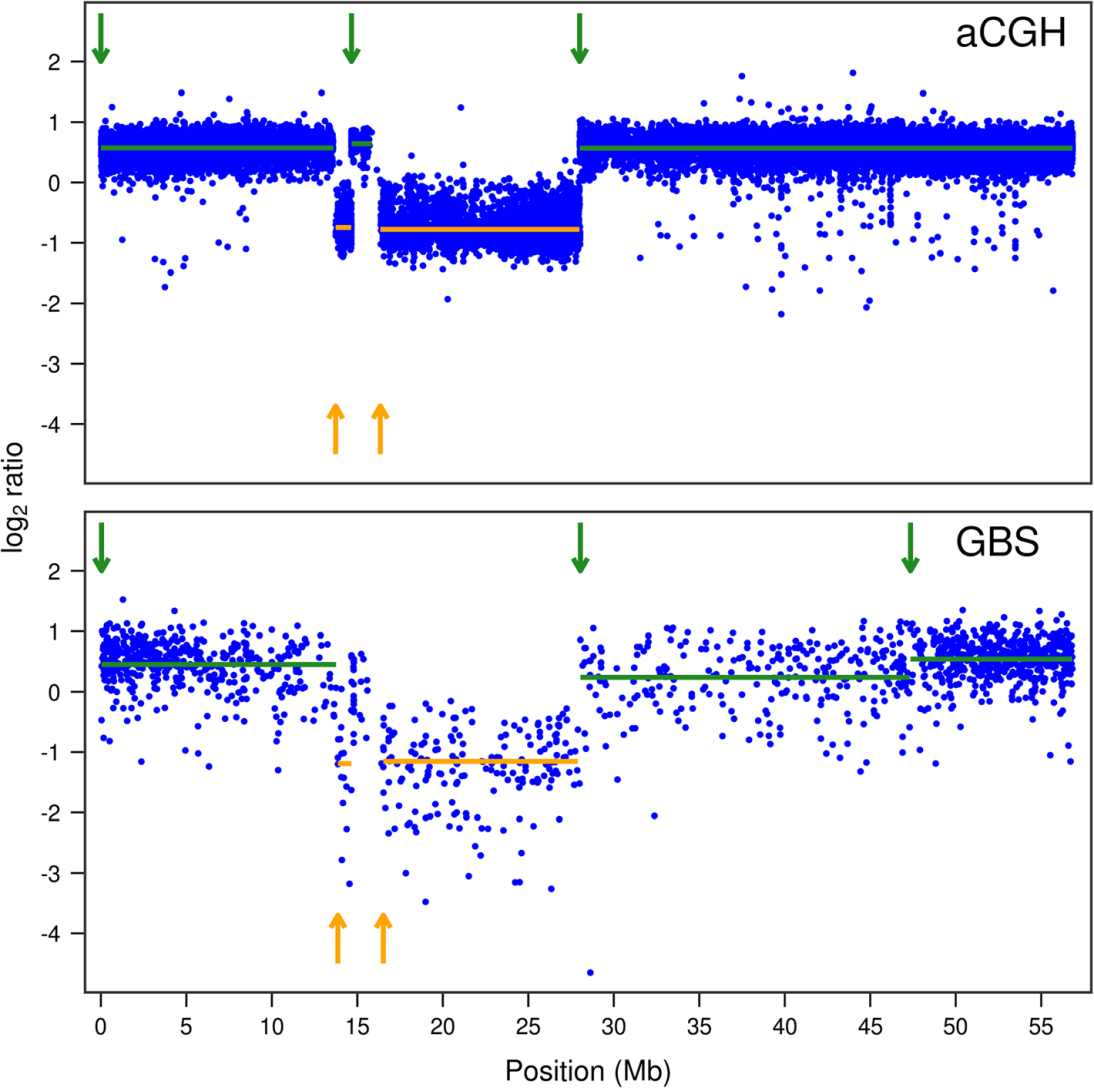
aCGH and GBS log_2_ ratio profiles showing an unusual succession of events on chromosome 1 of individual FNMN0066. Duplications and hemizygous deletions are indicated in green (top arrows) and orange (bottom arrows) respectively. Arrows indicate the starting points of the events and solid horizontal lines indicate the span (x-axis) and mean log_2_ ratio (y-axis) of the segment. Note that the automated GBS approach missed the middle duplication around position 15 Mb and split the last duplication into two separate events. Both datasets, however, outline the same series of duplications and hemizygous deletions

The size distribution of the events found in the complete dataset was similar to that reported for lines assessed by aCGH, homozygous deletions being on average smaller than hemizygous deletions or duplications (Fig. 4). The GBS CNV call set included exceptionally large events that exceeded previous records reported in this population by Bolon et al. [14] and to our knowledge in any fast neutron population. We indeed found an 11.2 Mb-long homozygous deletion (previous record 8.1 Mb) as well as 11.6 Mb-, 10.4 Mb- and 10.0 Mb-long hemizygous deletions (previous record 9.3 Mb). We also found a near-complete chromosome duplication (larger than 50 Mb) of Chr19 in an individual, a size similar to a duplication reported by Bolon et al. [14]. Except for the 10.4-Mb hemizygous deletion, which was found in an individual that was not assessed by aCGH, all these events were also validated by aCGH data. A suspected 17.6-Mb hemizygous deletion called from the GBS data of the first library was shown to actually consist of two separate events upon the addition of the sequencing data of the second library (see below). An unusual succession of duplications and hemizygous deletions was detected by both aCGH and GBS on Chr01 of an individual (Fig. 5), raising questions as to the mechanisms that might explain it.

### Effect of the larger size-selection GBS protocol

We tested the effect of sequencing a GBS library targeting larger restriction fragments on the performance of CNV calling from GBS data. Our rationale was that this would increase the resolution of the GBS approach by diversifying the genomic regions used to call CNVs. As expected, the reads generated from this second GBS library mapped to larger in silico-digested restriction fragments, spanning fragments ranging from ~ 150 to 350 bp, whereas the first library generated fragments ranging from ~ 50 to 225 bp (Additional file 1: Figure S5). As a result of the overlap between the fragment sizes found in the two libraries, about half of the informative 1-kb bins contributed by the second library were exclusive to this library, whereas the other half was already covered by the first library (Additional file 1: Figure S6). This resulted in an increase from 32,741 to 47,036 of the number of informative 1-kb bins used for calling homozygous deletions and duplications, and from to 28,439 to 36,709 for the dataset used to call hemizygous deletions. This increased resolution and sequencing depth made it possible to call two additional homozygous deletions measuring 22 and 60 kb, as well as one additional 128-kb hemizygous deletion (among the set of events identified by aCGH). The addition of this new library thus effectively brought our detection threshold for homozygous deletions close to 60 kb. The combined data of the two libraries resulted in a call set of 108 homozygous deletions, 36 hemizygous deletions and 20 duplications among the 79 mutant lines assessed (Additional file 3).

### Validation of the GBS approach using PCR and ddPCR

We selected the smallest four duplications, eight hemizygous deletions and fourteen homozygous deletions found in the set of lines that were only assessed by GBS to provide independent validation data for these events near the detection threshold. In addition, two homozygous deletions found in the control line M92–220-Long R6–1 were assessed by PCR and ddPCR.

**Fig. 6:**
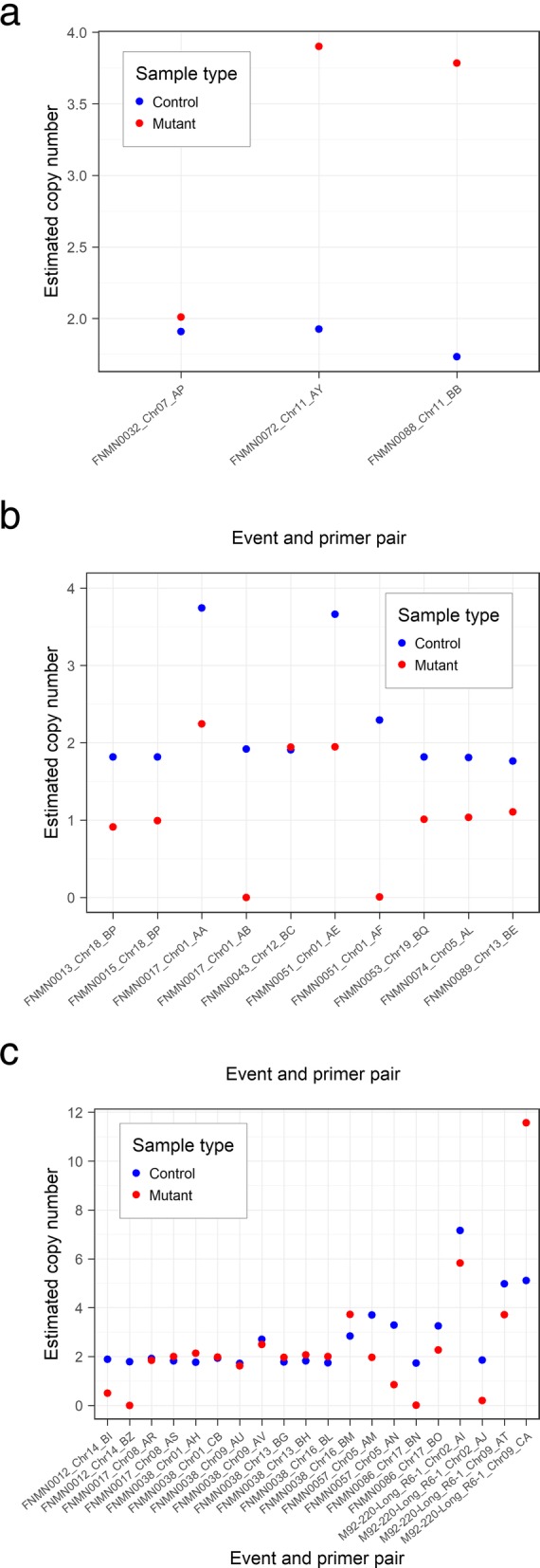
Copy number of target regions in control and mutant samples as estimated by ddPCR for putative (**a**) duplications, (**b**) hemizygous deletions and (**c**) homozygous deletions. Estimated copy number was calculated by dividing the concentration (in number of DNA templates per μl) obtained for the primer pair considered by the concentration obtained for the reference BY primer pair in the same sample, times two (considering diploidy). X-axis labels include the name of the mutant sample in which the event was called, the chromosome on which the event is located, and the primer pair used to assess the number of copies (see Additional file 7 for primer information). The putative duplication in FNMN0044 and the putative homozygous deletion on chromosome 6 of FNMN0064 were omitted from these graphs because of their very high copy number (> 20)

Of the four duplications, two were confirmed by ddPCR, whereas one was found to be a false positive call and another one yielded inconclusive results due to the high number of loci (> 5) amplified by the primers (Fig. 6a and Additional file 4). Out of the eight hemizygous deletions, five were validated, while two putative hemizygous deletions turned out to be homozygous deletions (the ones in FNMN0017 and FNMN0051) and the remaining one was a false positive call (Fig. 6b). Evidence that the putative hemizygous deletions in FNMN0017 and FNMN0051 were in fact homozygous deletions was provided by the fact that, in both cases, one of the primer pairs yielded a copy number of 0 in the mutant and 2 in the control, whereas the other primer pair yielded a copy number of 2 in the mutant and 4 in the control. ddPCR results were highly reproducible across assays, the five validated hemizygous deletions calls having a mutant:control copy number ratio of 0.56 ± 0.05 (mean ± s.d.) whereas the 10 cases in which the mutant and the control had the same copy number had a mutant:control copy number ratio of 1.07 ± 0.09 (mean ± s.d.).

Of the 16 homozygous deletions, five were readily validated by simple PCR and were not assessed by ddPCR (Additional file 1: Table S1). Three other deletions for which the two primer pairs yielded contradictory results using PCR (in FNMN0012, FNMN0086, and chromosome 2 of M92–220-Long R6–1; see Additional file 1: Table S1) were confirmed as the ddPCR assay provided evidence that the associated primer pairs either did not amplify in the mutant or amplified more than one locus in the control lines and consistently amplified a lower number of copies in the mutant (Fig. 6c). The results obtained for the putative deletion in FNMN0057 were consistent with this region being deleted in the mutant, although the fact that none of the two primer pairs designed for that event were specific to the intended target prevented any robust conclusion to be reached (Fig. 6c). Two events (on chromosome 9 of M92–220-Long R6–1 and on chromosome 6 of FNMN0064) were targeted by primer pairs that amplified so many loci that no conclusion could be reached in these cases. Finally, five homozygous deletions (the four putative homozygous deletions on FNMN0038 and the one on FNMN0017) proved to be false positives as the ddPCR provided evidence of the amplification of approximately two copies of the target regions in both the control and mutant lines. The fact that these five deletions were false positives could largely be explained by the lower coverage for these two samples, which made them more susceptible to spurious CNV calls (Additional file 1: Figures S7 and S8).

As a result of the validation by aCGH, PCR and ddPCR assays, the set of CNVs found by GBS could be updated to remove false-positive calls and change the CNV type of the hemizygous deletions that were in fact homozygous deletions. Therefore, the final set of CNVs called from GBS comprises 105 homozygous deletions, 32 hemizygous deletions and 19 duplications (see Additional file 1: Figure S9 for a graphical summary).

### Number of genes affected among the lines assessed by aCGH

The total number of genes affected among the lines assessed by aCGH was 1185 for homozygous deletions, 1267 for hemizygous deletions, and 7386 for duplications (each number including duplicate counts of genes that were affected independently in more than one line). Among these, 1154 homozygous deletions (97.4% of total) were due to lesions that were also detected by GBS. Similarly, 1024 gene disruptions due to hemizygous deletions (80.8% of total) and 7271 gene duplications (98.4% of total) were associated with lesions detected by GBS. Therefore, collectively, only 389 gene deletions or duplications were due to the 41 lesions missed by GBS, whereas the 55 events that were detected accounted for a total of 9449 genes affected.

## Discussion

### CNV calling with GBS: a proof of concept

The first objective of our study was to develop and optimize an approach for CNV discovery in fast neutron populations from standard GBS data. Our results demonstrate that GBS can indeed be used as a high-throughput and low-cost approach for CNV assessment in fast neutron mutants. Using a pipeline specifically designed for this purpose, we were able to detect over 150 events among 79 fast neutron mutants, including exceptionally large or complex events beyond what had been previously reported in the same fast neutron population.

Validation using aCGH data allowed us to quantify the performance of the GBS approach and optimize parameters such that a very low false discovery rate could be attained. Although validation using PCR and ddPCR assays did reveal a few false positive calls, these likely represented almost all false positive calls in our dataset given that we focused on the smallest events, which are the most difficult to detect. For example, all five false positive homozygous deletion calls were supported by only three 1-kb bins, while all homozygous deletions supported by four 1-kb bins were supported by the PCR/ddPCR assays. Using these assays to target the smallest events therefore helped in identifying conditions (i.e. lower read coverage and only three supporting bins) in which homozygous deletion calls should be regarded more cautiously.

Even though the GBS approach has higher resolution for homozygous deletions, it was still able to detect large hemizygous deletions and duplications with high accuracy as demonstrated by both aCGH and ddPCR assays. A caveat of the validation approach used here, however, is that hemizygous deletions and duplications were called visually and not computationally from the aCGH data, such that the actual number of such events that are detectable by aCGH but not by GBS may be higher than reported here. In two cases, CNVs initially called as hemizygous turned out to be homozygous deletions as assessed by ddPCR, but this is not a major problem since any follow-up studies on such erroneously called mutants should generate independent data that would provide a correct picture.

The biggest advantage to this GBS approach is arguably the very high level of multiplexing to which it is amenable, which could make it possible to assess hundreds or thousands of individuals at relatively low cost, providing the capacity to address new questions regarding the impacts of fast neutron mutagenesis and broaden the application range of such populations. As of 2019, the cost of running 96 samples on aCGH (including labor but excluding the DNA preparation and analysis steps) is approximately 73,000 USD. On the other hand, the cost for performing GBS as described here (three sequencing chips of one size-selection library and one sequencing chip of another, again excluding DNA preparation and analysis) was approximately 4000 USD, representing a ~ 18-fold reduction in cost. Performing the analysis on a single sequencing chip for each of two size-selection libraries, which would perhaps be the most cost-effective approach, would bring the cost down to approximately 2500 USD per 96-sample plate, resulting in a ~ 29-fold reduction in cost relative to aCGH. Our method therefore fills a gap in the range of methods available for screening irradiation-induced CNVs by providing a low-cost method with reasonable resolution, whereas previously existing computational methods either relied on high-depth WGS which is hardly affordable for large populations (e.g. [10, 15]), or on low-depth WGS which (at least as the methods were designed) were limited to events larger than 300 kb [29, 30].

The GBS approach described here would be most useful in the context of reverse genetics, where one seeks to identify individuals bearing particular mutations rather than to find the mutation responsible for a particular phenotype. Indeed, the resolution of GBS was expectedly several times lower than that of aCGH, which limits its applicability to forward genetics as several smaller events will be missed. However, this lower resolution is a reasonable trade-off for reverse genetics given the much lower cost and higher throughput that can be afforded by using a GBS protocol. Moreover, even though GBS detected only slightly more than half of the events detected by aCGH, the vast majority of gene deletions or duplications in the population will still be detected, as the largest events (which also span the largest number of genes) are easily found by GBS. Given these results, we suggest a paradigm in which GBS can be used as a first step to screen entire fast neutron populations, after which whole-genome sequencing can be performed on individuals of interest to resolve event breakpoints and discover variants that were overlooked by GBS.

### Using the SNP genotyping capabilities of GBS

Fast neutron irradiation has been shown to also induce single-base substitutions and small indels (e.g. [7, 15]). In this context, it should be noted that the aCGH technology will likely miss these lesions as well as deletions up to a few kb, which similarly limits its applicability to forward genetics. On the other hand, the sequencing technology employed by GBS has the ability to discover SNPs and indels. While calling mutations presents a few challenges to avoid confounding them with sequencing errors, the fact that ~ 30,000 bins were covered by a mean number of reads > 7 in our dataset suggests that there are plenty of well-covered fragments from which to confidently call putative nucleotide substitutions and indels even in a 96-plex library. Although the calling of base substitutions and indels was outside the scope of this study, larger-scale characterization of fast neutron populations using GBS should make use of the genotyping capabilities of this technology for a more comprehensive description of the mutation spectrum. An interesting outcome related to the use of GBS data in this study is that SNP calls were useful for identifying contamination and genetic heterogeneity in the mutant population. The SNP markers called by GBS identified individuals that were not of the M92–220 genetic background as well as regions of natural heterogeneity among the M92–220 seed stock originally subjected to mutagenesis. Although aCGH data can also be used to achieve these goals (e.g. [14, 31]), SNP genotyping through sequencing allows for a more straightforward way to do so.

### Use of methylation-sensitive enzymes

An interesting aspect of the GBS protocol used here is that it naturally adjusts to provide higher resolution in gene-rich areas due to the use of a methylation-sensitive enzyme (*Ape*KI), effectively concentrating sequencing efforts in regions that are more likely to contain genes. Due to the reduced cutting frequency of *Ape*KI in the largely methylated heterochromatic regions, the resolution of the GBS approach was indeed much higher in euchromatic than heterochromatic regions, as evidenced by the distribution of the informative 1-kb bins across the genome (Additional file 1: Figure S1). The 120-kb deletion that went undetected by GBS, notably, was located in a pericentromeric region (at position ~ 27 Mb on chromosome 14), as were the three largest hemizygous deletions that went undetected by GBS, which were all located in the same pericentromeric region of chromosome 9 in FNMN0041. This higher sensitivity in euchromatic regions likely also contributes to the very high percentage of gene disruptions that are detected by GBS among those predicted from aCGH.

### Modularity of the GBS approach

The GBS approach described here is modular in the sense that sequencing data generated from different libraries or different sequencing runs can be sequentially added to reach the resolution required for a given application. In this study, we sequenced a first library ranging from ~ 50–225-bp restriction fragments on three Ion Proton chips and a second library ranging from ~ 150–350-bp fragments on a single Ion Proton chip. These data allowed us to assess the relative contributions of sequencing depth and library content on the performance of CNV calling. In our validation using aCGH data, the addition of a second library improved the resolution of the GBS approach by enabling two additional aCGH-confirmed homozygous deletions and one additional hemizygous deletion to be detected. Resampling simulations (presented in Additional file 1) showed that the benefits of deeper sequencing largely plateaued after two sequencing runs of a 96-plex library, and that the marginal benefits of sequencing a different library were higher than those of deeper sequencing of the same library. For the purposes of CNV assessment in this population, a good trade-off between cost and performance would therefore be obtained by performing two sequencing runs of one library and one run of another library, or even only a single run of each. One drawback to keep in mind, however, is that uneven sequencing of the samples may make CNV discovery more difficult in samples that are less deeply sequenced (see e.g. Additional file 1: Figure S10). For example, the five false-positive homozygous deletions that were found by the PCR and ddPCR assays were identified in individuals that suffered from low coverage (Additional file 1: Figures S7 and S8). Visual analysis of the log_2_ ratio profiles of the individual (FNMN0032) bearing the duplication that was revealed as a false positive call by ddPCR also revealed that this segment was declared significantly different from its surroundings only because it was embedded between two homozygous deletions, such that the use of a more stringent threshold for calling duplications may have prevented this erroneous call (Fig. S11). These observations highlight the need for users of the GBS approach to assess the results critically and visually using the log_2_ ratio profiles despite our best efforts at automating the CNV calling process.

In our particular study, costs were reduced due to the fact that both libraries were derived from a single digestion-ligation step and diverged only at the size-selection step. The cost of performing additional sequencing on libraries that are ready for sequencing is also lower than going through the whole library preparation and sequencing process. One advantage of this modularity is that results can be reassessed as soon as new data is generated, such that researchers can evaluate the needs for further sequencing in a stepwise manner and thus progress smoothly towards a final dataset that suits their needs. Although there comes a point where WGS will be cheaper than adding “modules” (different libraries or additional chips), we argue that this modularity allows for flexibility in adjusting to the needs of a particular application. Moreover, increased sequencing of a GBS library guarantees high coverage of particular positions in the genome, which is likely more useful in calling duplications and hemizygous deletions than low-depth WGS (e.g. 1X) would be.

### New insights into the soybean fast neutron population

Although we assessed fewer mutants in this study (79 by GBS, including 19 by aCGH) than the 264 mutants surveyed by Bolon et al. [14], we were able to expand the size range of previously reported events. We indeed found four homozygous or hemizygous deletions larger than 10 Mb as well as a near-complete chromosomal duplication. Collectively, these results reinforce the notion that the soybean genome is highly resilient to structural variation, as such large events could manifestly be sustained without lethality. The larger size of duplications and hemizygous deletions as compared to homozygous deletions is likely to be a consequence of both 1) the higher detection threshold of duplication and hemizygous deletion events and 2) the stronger selection pressure against large homozygous deletions. Given that the lines assessed here went through several generations (ranging from 5 to 11) of self-pollination after mutagenesis, deletions that remained hemizygous are indeed very likely to have done so because complete deletion of one or several of the affected genes would have been lethal.

Our study also found an event with a very unusual structure on chromosome 1 that appeared to include an alternating succession of duplications and hemizygous deletions (Fig. 5). One possible explanation for this could be that one of the copies of chromosome 1 in this individual lost its centromere during irradiation, and both extremities became attached to some other chromosome in the cell. Resequencing and/or karyotyping of this individual might provide data to test this hypothesis and yield insights into the kinds of aberrations that can be induced by fast neutron irradiation. We hypothesize that the higher throughput afforded by the GBS approach will enable the discovery of several similarly unusual events in other individuals of this fast neutron population. Complex rearrangements, such as inversions and translocations, have been previously reported in this population [17, 18] and in gamma-irradiated poplars [29]. However, such events are likely more common than have been identified to date.

### Perspectives on potential applications

The reduced cost of GBS potentially makes it conceivable to assess hundreds or even thousands of individuals in existing or future populations of fast neutron mutants. Such an increase in the number of samples that can be assessed would make it possible to address questions that were not accessible before. For example, the assessment of thousands of individuals would make it possible to identify genomic regions that are never deleted and could thus be thought to contain essential genes. Moreover, striking mutations that did not necessarily result in noticeable phenotypes in the initial forward screening of the population could be investigated further and result in new insights into gene function. Current plans for the soybean fast neutron population envision the assessment of hundreds of other individuals using the GBS approach described here, so as to gain unprecedented insights into this population and make this genomic resource more useful to the scientific community. More broadly, this GBS approach could be used to screen any population in which large CNVs are known or expected to be found. For example, a similar approach has recently been used to track large introgressions associated with variation in copy number in wheat and barley germplasm (Jens Keilwagen, personal communication). If sensitivity is to be valued over precision, or if known CNVs are to be assessed by GBS, analysis parameters can also be made less stringent in order to call CNVs from as few as one or two 1-kb bins.

## Conclusion

Our study provides a proof of concept that GBS can be used to assess copy number variation in a set of fast neutron mutants. This method could be used to allow population-scale assessments in this and other collections of mutants, an endeavour that is cost-prohibitive using previous technologies. Improvements and adjustments to the GBS workflow described here could also be made to enable the use of standard GBS data to assess CNVs in elite cultivars or advanced lines. This would be especially powerful, as it might enable researchers to revisit the tremendous amount of already-existing GBS data and extract the CNV information that remains undiscovered.

## Methods

### Plant material and DNA extraction

The DNA of 92 fast neutron-mutagenized soybean plants derived from cultivar M92–220 (population described by Bolon et al. [13]) was extracted at the University of Minnesota using a Qiagen DNeasy plant DNA kit. The 92 samples were selected at random from within a larger core collection of diverse mutants that is currently being developed. For each genotype, DNA samples were extracted from a single leaf of a single mutant plant. These plants were grown in the field in 2016 in Saint Paul, MN, and ranged from the M5 to the M11 generation. DNA samples of four wild-type M92–220 individuals were also used as controls, thus bringing the total number of samples to 96. To control for heterogeneity in the control sample, the wild-type M92–220 individuals were all previously derived from a single individual known as M92–220-Long.

### Array comparative genomic hybridization

Array CGH was performed at the University of Minnesota for 19 of the mutant DNA samples using a 940 K CGH array developed for soybean (Agilent Technologies, Santa Clara, CA, USA). The detailed information about this array platform can be found in accession number GPL22907 in the National Center for Biotechnology Information Gene Expression Omnibus (GEO) (http://www.ncbi.nlm.nih.gov/geo). For each of the 19 hybridizations, the DNA of the fast neutron line to be assessed was labeled with Cy3 whereas control (wild-type M92–220) DNA was labeled with Cy5. Following hybridization, arrays were read with a scanner and fluorescence values were used to compute the log_2_ of the Cy3/Cy5 fluorescence ratio, which we will refer to as the aCGH log_2_ ratio. The methods used to scan and compute the log_2_ ratios were performed as previously described [18]. The 19 individuals subjected to aCGH were not randomly selected but rather chosen to maximize the spectrum of CNVs predicted from the GBS data.

### Genotyping-by-sequencing

All 96 DNA samples were sent to the Plateforme d’analyses génomiques at Université Laval for GBS library preparation and sequencing. A 96-plex GBS library was prepared following a standard protocol (see [22]) using the enzyme *Ape*KI and a size-selection step targeting 50–225 bp restriction fragments. This library was sequenced on four chips of an Ion Proton sequencer (Thermo Fisher Scientific, Waltham, MA, USA), for a total yield of 199 million single-end reads of an average length of 149 bp. However, one of the runs yielded only three million reads due to technical issues, such that the number of reads generated corresponds to what would be expected from three sequencing runs.

### Calling copy number variation using aCGH data

The aCGH data output provided log_2_ ratio profiles that could readily be used for copy number variation calling. Homozygous deletions were detected by segmenting the log_2_ ratio profiles using a robust segmentation algorithm implemented in the R package *robseg* [32] (additional details in Additional file 1). A segment was labeled as a deletion if its mean log_2_ ratio was below − 3 as this value (representing a ratio of 1/8) was heuristically found to accurately separate true positives from false positives. Contiguous deletions that had been partitioned in segments with different mean log_2_ ratios were merged and considered as a single deletion. The span of a homozygous deletion was defined as extending from the 3′ end of the first probe supporting the deletion to the 5′ end of the last probe supporting it. Homozygous deletion calls were filtered out if the event was located in regions of naturally occurring (i.e. not fast neutron-induced) heterogeneity among the M92–220 seedstock originally subjected to mutagenesis (see Additional file 1). Homozygous deletion calls supported by less than three probes were also filtered out as they potentially represented false positives and were not expected to be detected by the GBS approach in any case. Hemizygous deletions and duplications proved more challenging to call computationally from aCGH data due to their less extreme log_2_ ratio values. Indeed, we found it hard to find a unique set of parameters that would yield results that matched what could be observed from the log_2_ ratio profiles, so we called them visually from the log_2_ ratio profiles instead. In the context of this study, the term “duplication” refers to any increase in copy number.

### Calling copy number variation using GBS data

CNV calling from GBS data was more challenging as the sequencing data could not be readily converted to log_2_ ratio-like values. As a consequence, we devised a custom analysis pipeline for calling CNVs from GBS data. Raw sequences were processed using the Fast-GBS pipeline [33], which includes the following steps: demultiplexing with Sabre [34], adapter trimming with Cutadapt [35], alignment to the reference genome using BWA [36], and finally SNP- and indel-calling using Platypus [37]. At the alignment step, we used a composite reference genome combining the version 2 assembly of the *Glycine max* genome (Gmax_275_v2.0) [38, 39] and the mitochondrial and chloroplastic DNA sequences retrieved in August 2017 from SoyBase [40]. We included the mitochondrial and chloroplastic reference sequences in order to avoid the numerous reads originating from the organellar genomes (10.5 and 4.0% of the ~ 130 M mapped reads aligned to the chloroplastic and mitochondrial genomes, respectively) from aligning to potential homologous sites in the nuclear genome. CNV calling from GBS data relied entirely on the position and count of reads aligned to the nuclear reference genome. SNP calls were not used for CNV calling per se but were useful in revealing 11 putative mutant lines that showed signs of contamination by other germplasm. The 11 “contaminated” fast neutron lines (see Additional file 1: Figure S12) were not considered further in this study as their CNV calls might be due to naturally occurring variation rather than fast neutron irradiation. The SNP calls were also useful for identifying genomic areas of heterogeneity among M92–220 individuals (see Additional file 1). We used the heterogeneous regions identified through SNP calls (Additional file 1: Figure S13) for filtering out CNV calls overlapping these regions from both aCGH and GBS data.

Our pipeline for CNV calling from the alignments starts by dividing the reference genome (excluding scaffolds) into 1-kb bins and counting, for each individual, the number of reads mapping to each bin. This bin size provided a natural framework to think about our data, as most reads originating from a single restriction fragment will map to the same bin, whereas reads originating from different restriction fragments will likely map to different bins. Reads are tallied according to their 5′ end position on the + strand and filtered for mapping quality, after which read counts are normalized across the population by multiplying the read counts for each individual by a factor such that all individuals have the same number of reads. The minimum mapping quality was set to 20 for calling homozygous deletions and duplications, and to 35 for calling hemizygous deletions (see Additional file 1 for details on how and which parameters were optimized for different categories of CNVs). Three filtering steps are then applied to reduce the dataset to a set of bins deemed informative for the purposes of CNV calling. The first filtering step consists in removing bins that have a mean count of reads per individual > 150, so as to remove bins that present exceptionally high read counts, possibly due to paralogous loci or organellar sequences that would have mapped to the nuclear genome despite our precautions. The second filtering step consists in removing bins with a mean read count per individual < 7 (< 8 for hemizygous deletions), as these bins do not provide enough power for discriminating true variation in copy number from random variation. The third and last step consists in removing bins with a very high variance in the mean read count: when the ratio of the variance of the number of reads per individual to the mean number of reads per individual is > 3, we eliminate these as the high variance in these bins might lead to spurious CNV calls.

In order to translate the read count data into information that could be used for CNV calling, we computed a log_2_ ratio value analogous to the one obtained from aCGH for every informative bin of every individual. This log_2_ ratio was computed by dividing the number of reads (+ 1 to avoid undefined logarithms) observed for an individual in a given bin by the overall mean number of reads (+ 1) observed per individual in this bin, and then computing the base 2 logarithm of this ratio. Because this approach compares relative read depth across samples rather than to the reference, any structural differences between the reference genome (Williams 82) and the M92–220 genome will have a minimal impact on CNV calling, with the exception of translocations which may cause regions that are contiguous in Williams 82 to be far apart in M92–220 or vice-versa. The GBS log_2_ ratio profiles were segmented using the R package robseg (as above for the aCGH data) and segments were considered as homozygous deletions if their mean log_2_ ratio values were < − 2.5, as hemizygous deletions if their mean was between − 0.5 and − 2.5, and as duplications if their mean was > 0.2. Homozygous deletion calls supported by fewer than three bins or hemizygous deletions or duplications supported by fewer than six bins were discarded as many of these proved to be false positives when validated against the aCGH data. For homozygous deletions, we defined the size of the deletions by computing two measures: 1) a “minimum deletion span” that went from the 3′-end of the first bin supporting the deletion to the 5′-end of the last bin supporting it, and 2) a “maximum deletion span” that went from the 5′-end of the leftmost informative bin not supporting the deletion until the 3′-end of the rightmost bin not supporting the deletion. For hemizygous deletions and duplications, the equivalent of the minimum span was used as a single measure of event size.

### Validation of the GBS approach using aCGH data

We assessed the performance of the GBS approach by comparing the set of events found by GBS and aCGH in the 19 fast neutron individuals for which aCGH data was available. For this purpose, any GBS event that overlapped with an event of the same type in the aCGH dataset was considered a true positive, whereas any event detected by GBS but not by aCGH was considered a false positive. Events that were found by aCGH but not by GBS were simply considered undetected by GBS. Although aCGH has its own disadvantages and issues (such as inconsistent hybridization efficiencies across probes and possible cross-hybridization among paralogous sequences), it was taken here as a gold standard given that its resolution was expected to be much higher than that of GBS, such that any event that is detectable by GBS should also be detected by aCGH. However, we cannot rule out the possibility that some of the smallest events detected by aCGH may be false positives, or that other small events may have been missed.

### Effect of a different size-selection protocol

In order to increase the resolution of the GBS approach, we generated and sequenced a second GBS library derived from a size-selection step targeting larger restriction fragments on an additional Ion Proton chip. The sequencing run yielded a total of 80 million single-end reads of an average length of 154 bp. The “small” and “large” libraries will be referred to hereafter as the first and second GBS libraries, respectively. CNV calling was first performed on the data from the first GBS library to assess the performance of a simple, standard GBS protocol, and then on the data from the two combined libraries to assess the effects of adding a different library. When tallying the reads from the second GBS library, we used the 3′ position for the reads that mapped to the “+” strand and the 5′ position for the reads that mapped to the “-” strand instead of using the 5′ position for both strands as we did for the first library. This modification to the pipeline aimed at preventing two reads generated from different strands of the same restriction fragment from falling in different 1-kb bins, which might have happened given that the fragments generated by this library were longer. CNV-calling parameters were also slightly modified to process the combined data of the two libraries (see Additional file 1 for details). Resampling simulations were carried to thoroughly evaluate the relative contributions of deeper sequencing and sequencing of different libraries on the performance of the GBS approach; the related methods and results are described in Additional file 1.

### Validation of the GBS approach using PCR and ddPCR

In order to provide an independent assessment of the accuracy of CNV calls near the detection threshold, we selected the smallest four duplications, eight hemizygous deletions, and 14 homozygous deletions among the set of lines that were assessed by GBS but not by aCGH and assessed them with PCR and/or ddPCR assays; the number of events selected from each type reflected the relative frequency of these types of events in our dataset. In addition, we sought to validate two homozygous deletions which were each found in two replicates of a control line (M92–220-Long R6–1 and M92–220-Long R6–2).

Up to two primer pairs targeting each event were designed using an in-house pipeline which combined Primer3 [41] for primer design and Exonerate [42] for specificity checking (see Additional files 1, 4, 5, 6, 7 and 8 for detailed methods and results related to this section). Homozygous deletions were validated by observing the presence or absence of an amplification product following PCR using diagnostic primers in control and mutant lines. The failure of a diagnostic primer pair to amplify the target region in a mutant was considered as evidence that this region was deleted in the mutant. Duplications and hemizygous deletions were instead assessed by ddPCR to provide an absolute measure of copy number of the target region in mutant and control lines, since these could not be assessed by the mere presence/absence of an amplification product. Duplications were considered validated if the number of copies observed in the mutant was approximately twice that observed in the control line, whereas hemizygous deletions were deemed to be confirmed if the number of copies observed in the mutant was approximately half that observed in the control line. Homozygous deletions for which amplification of the diagnostic primers was observed in the mutant were also subjected to ddPCR to verify whether amplification might have occurred due to DNA contamination or due to the primers amplifying more than one locus.

### Analysis of the number of genes affected

To estimate the proportion of gene disruptions that are found by GBS among those identified by aCGH, we performed a simple analysis to compute the number of genes that are affected (by any type of CNV) for each event. Gene model coordinates for the genome assembly version 2 of soybean were retrieved from SoyBase [40] in January 2019. We then simply computed the number of gene models overlapping with each of the lesions and tallied them by type and according to whether or not they were found by the GBS dataset generated from the use of the combined libraries. A given gene model was allowed to be counted more than once if it was affected independently by CNV events occurring in different lines.

### Software used and implementation

Unless otherwise stated, all analyses were performed in R version 3.4.3 or 3.5.1 [43]. Bioconductor packages Rsamtools [44] and GenomicAlignments [45] were used for reading and manipulating the aligned reads. The R package that we developed for implementing the CNV-calling pipeline described here, named *delgbs*, is publicly available on GitHub (github.com/malemay/delgbs). Starting from sorted and indexed .bam files, the segmentation and CNV calling of the data generated from the first GBS library took 10 min to run on a single CPU and required 2 Gb of RAM, and it thus amenable to run on any personal computer.

## Additional files

Additional file 1: Supplementary methods and supplementary Figs. S1 through S35 (DOCX 13364 kb)

Additional file 2: Copy number variants found in the 19 individuals subjected to aCGH. ID: the identifier of the mutant; CNV_type: the type of CNV (homdel = homozygous deletion, hetdel = hemizygous deletion, dup = duplication); chr: the chromosome on which the CNV is located; start: the starting position of the CNV; end: the end position of the CNV; kbp: the estimated size of the CNV in kbp; n_probes: the number of aCGH probes supporting this CNV; mean_log2: the mean log_2_ ratio of the probes across this CNV; found_by_GBS: whether or not this CNV was detected by the GBS approach. (CSV 6 kb)

Additional file 3: Copy number variants found in the 79 mutants sequenced by GBS using the two combined libraries. ID: the identifier of the mutant; CNV_type: the type of CNV (homdel = homozygous deletion, hetdel = hemizygous deletion, dup = duplication); chr: the chromosome on which the CNV is located; start: the starting position of the CNV; end: the end position of the CNV; kbp: the estimated size of the CNV in kbp; n_bins: the number of 1-kb bins supporting this CNV; mean_log2: the mean log_2_ ratio of the bins across this CNV; validated_by_CGH: whether or not this CNV was validated by the aCGH dataset; validated_by_PCR: whether or not this CNV was validated by the PCR/ddPCR assays. (CSV 11 kb)

Additional file 4: Copy number of templates amplified by 37 primer pairs in mutant and control lines as assessed by a ddPCR assay. ID: the identifier of the mutant; CNV_type: the type of CNV (homdel = homozygous deletion, hetdel = hemizygous deletion, dup = duplication); chr: the chromosome on which the CNV is located; start: the starting position of the CNV; end: the end position of the CNV; primer_pair: the identifier of the primer pair that was used for that assay; mutant_conc: the concentration (in number of DNA templates per μl) measured in the mutant for that assay; mutant_ref: the concentration (in number of DNA templates per μl) measured using reference primer pair BY in that mutant; mutant_n: the copy number of the templates amplified by primer_pair in the mutant as determined by dividing mutant_conc by mutant_ref; control_conc: the concentration (in number of DNA templates per μl) measured in the control for that assay; control_ref: the concentration (in number of DNA templates per μl) measured using reference primer pair BY in the control; control_n: the copy number of the templates amplified by primer_pair in the control as determined by dividing control_conc by control_ref; ratio: the ratio of the copy number of the mutant to the copy number of the control for the templates amplified by primer_pair. (CSV 4 kb)

Additional file 5: Description of the CNVs selected for validation by PCR/ddPCR and summary of the results. ID: the identifier of the mutant; CNV_type: the type of CNV (homdel = homozygous deletion, hetdel = hemizygous deletion, dup = duplication); chr: the chromosome on which the CNV is located; start: the starting position of the CNV; end: the end position of the CNV; kbp: the estimated size of the CNV in kbp; n_bins: the number of 1-kb bins supporting this CNV; mean_log2: the mean log_2_ ratio of the bins across this CNV; primer_pair1: the identifier of the first primer pair designed for that CNV; primer_pair2: the identifier of the second primer pair designed for that CNV (N/A means that there is no second primer pair for that CNV); validation_status: a description of the conclusion reached for that particular CNV based on the PCR and ddPCR results. (CSV 2 kb)

Additional file 6: Primer3 settings file used for the primer design of the PCR and ddPCR primers. (TXT 955 bytes)

Additional file 7: Description of the primers used for the PCR and ddPCR assays. pair_id: the identifier attributed to a particular primer pair; chromosome: the chromosome on which the intended target of the primer pair is located; start_pos: the starting position of the intended target of the primer pair on the chromosome reference sequence; end_pos: the end position of the intended target of the primer pair on the chromosome reference sequence; amplicon_length: the predicted amplicon length of the intended target; forward_primer_sequence: the sequence of the forward primer; reverse_primer_sequence: the sequence of the reverse primer. (CSV 4 kb)

Additional file 8: Concentration values output by the QuantaSoft software for the 130 ddPCR assays. Sample: the DNA sample used for that assay; primer_pair: the identifier of the primer pair that was used for that assay; concentration: the concentration (in number of DNA templates per μl) measured for that assay. (CSV 2 kb)

## Data Availability

The data of the 19 aCGH experiments and the sequencing data are available through the NCBI BioProject number PRJNA486165. The read counts of the two size-selection libraries at different mapping quality thresholds is available on Figshare (10.6084/m9.figshare.7122977.v1). The *delgbs* package is available on GitHub (github.com/malemay/delgbs). Requests for mutant seed should be addressed to R. Stupar (stup0004@umn.edu).

## Abbreviations

aCGH: array comparative genomic hybridization
CNV: copy number variation
ddPCR: droplet digital PCR
GBS: genotyping-by-sequencing
SNP: single-nucleotide polymorphism
WGS: whole-genome sequencing
