# Supplementary material for: Screening populations for copy number variation using genotyping-by-sequencing: a proof of concept using soybean fast neutron mutants
Source: BMC Genomics. 2019 Aug 6;20:634. doi: 10.1186/s12864-019-5998-1 (PMC6683502; doi:10.1186/s12864-019-5998-1)
Supplement: Supplementary file 1 — Supplementary methods and supplementary Figs. S1 through S35 (DOCX 13364 kb) [file 12864_2019_5998_MOESM1_ESM.docx]

**Supporting information for "Screening populations for copy number variation using genotyping-by-sequencing: a proof of concept using soybean fast neutron mutants"**

*Identification of contaminated individuals and heterogeneous genomic regions*

The SNPs called from the GBS data using the Fast-GBS pipeline (Torkamaneh et al. 2017) were not directly used for CNV calling. However, they allowed us to identify individuals that showed signs of contamination from other germplasm and to delineate genomic regions that were polymorphic due to naturally occurring heterogeneity among the original M92-220 seedstock subjected to mutagenesis.

SNP calls generated by Fast-GBS from the first library were subjected to a series of filters using vcftools (Danecek et al. 2011) in order to keep only SNPs that most likely represented true polymorphisms. We removed SNPs that either had more than 75% of missing data, more than 10% of heterozygous genotype calls, or a minor allele frequency below 5%. All indels, as well as SNPs with more than two alleles, were also removed from the dataset. A PCA performed using TASSEL (Bradbury et al. 2007) on this SNP dataset revealed that 11 of the 92 fast neutron mutants assessed had SNP profiles that set them apart from the rest of the population (Figure S12). Graphical analysis of the SNP profiles of these individuals showed that polymorphic SNPs tended to cluster together in certain genomic regions (Figure S14), which was consistent with the explanation of contamination by other germplasm. Since the polymorphic regions of these individuals may also harbor copy number variants, we chose to exclude them from further analysis in order to avoid 1) calling CNVs that were not induced by fast neutron irradiation and 2) inaccurately estimating the expected number of reads per 1-kb bin.

Following the removal of these 11 individuals, we subjected the SNP dataset to a second round of more stringent filters by keeping only SNPs with a minor allele frequency larger than 10% and for which at least 60% of the individuals were genotyped. The 184 SNPs that passed these filters clustered in 11 genomic regions (Figure S13) which were presumed to represent remnants of polymorphism that naturally occurred among the M92-220 seedstock originally subjected to fast neutron mutagenesis. The first and last SNPs found in these regions were used to delineate, respectively, the start and end of heterogeneous regions which were used to filter out CNVs called from both aCGH and GBS data, as these might be due to naturally occurring variation rather than mutagenesis. Two of these regions (both on Chr06) were manually expanded as they did not entirely encompass heterogeneity artifacts that were observed in the aCGH data. A single SNP found on chromosome 10 was not used to delineate a heterogeneous region as we were unsure as to whether it represented a true polymorphism.

*Optimization of the parameters for CNV calling from the GBS data of the first library*

We sought to optimize three parameters that may affect the sensitivity and specificity of our GBS pipeline for calling CNVs: 1) the minimal mapping quality for a mapped read to be considered; 2) the minimal mean number of reads per individual in a 1-kb bin for this bin to be kept; 3) the segmentation threshold used when segmenting the log_2_ ratio profiles.

The potential implications of varying the first two parameters are rather straightforward. Lowering the minimal mapping quality and the minimal number of reads per individual is expected to increase sensitivity by increasing the number of positions for which information is available. However, this is also expected to decrease specificity as incorrectly mapped reads or basing calls on shallowly covered bins could result in spurious CNV calls.

The role that the third parameter plays in CNV calling needs some more explanation. The segmentation algorithm implemented in our pipeline for CNV calling from GBS data (Fearnhead and Rigaill 2018) essentially aims at identifying breakpoints between segments of data points that have differing mean log_2_ ratios. In order to identify how many breakpoints there should be and where they should be placed, the algorithm seeks the segmentation with the lowest overall cost. The overall cost is the sum of two parts: the first part evaluates the fit of the segmentation to the data through a loss function, whereas the second part penalizes for the number of breakpoints (otherwise each data point would have its own segment). More precisely, the loss function measures how far the data points are from the value of the segment to which they belong. The loss function used as part of this study is robust to outliers as it increases quadratically as a function of distance until some value, and then remains constant; this effectively prevents data points with (potentially spurious) extreme values from driving the creation of their own segment, while still being statistically consistent. The parameter that we have sought to optimize here is the multiplier applied to the standard deviation estimate of the log_2_ ratio in order to obtain the value at which the cost function becomes constant; the higher this parameter is, the more sensitive the algorithm is to large values. For simplicity, we will refer to this parameter as the “segmentation threshold”. In the context of homozygous deletion calling from aCGH data, we used a segmentation threshold of 15, whereas lower values were used for GBS data. The penalty parameter was kept constant throughout this study at 2*log(n), where *n* is the number of data points to segment.

We tested four different minimal mapping quality values (mapq = 20, 25, 30 or 35), five different minimal number of reads (minr = 5, 6, 7, 8 or 9) and four different segmentation thresholds (segt = 4.5, 5, 5.5 or 6) in a factorial way, effectively resulting in 80 combinations being tested. For each of these combinations, we took the reads aligned by BWA and processed them through the whole CNV calling pipeline. Sensitivity and specificity were respectively assessed by counting, for each parameter combination, the number of true positives (calls found in the aCGH dataset) and the number of false positives (calls not found in the aCGH dataset). Homozygous deletions, hemizygous deletions and duplications were assessed separately in order to identify the best parameter combination for each of these event types.

For homozygous deletions, the highest number of true deletions that could be called by any single parameter combination without yielding false positive calls was 29 (Figure S2). Two parameter combinations (mapq = 25, minr = 7, segt = 6; mapq = 20, minr = 7, segt = 5.5) corresponded to this situation, but we chose the latter as it yielded the lowest number of false positive hemizygous deletion calls among the two. Other parameter combinations detected up to 31 homozygous deletions, but also resulted in several false positives being called, so we did not choose these parameter combinations as we prioritized specificity over sensitivity.

For hemizygous deletions, the best parameter combination (mapq = 35, minr = 8, segt = 4.5) was able to detect 13 events while declaring a single false positive, which was the lowest number of false positives called among all parameter combinations (Figure S3). Hemizygous deletions thus appeared to require more stringent parameters to be accurately called from GBS data. This is likely due to the reduced impact of a hemizygous deletion on the number of reads as compared to homozygous deletions, which makes hemizygous deletion calling more likely to be affected by incorrectly mapped reads or shallowly covered bins.

For duplications, none of the parameter combinations yielded any false positives, whereas the different combinations detected either 7 or 10 true positives. Since the combination that worked best for homozygous deletions was among those detecting 10 duplications, we chose this parameter combination as the standard for calling duplications. We also tested whether any of the combinations performed better at detecting the correct boundaries of duplications, as a few duplications observed by aCGH were either split into several duplications or merged into a single duplication according to the GBS calling pipeline. However, all parameter combinations resulted in exactly the same number of such inconsistencies, although the exact set of inconsistencies varied among the different combinations.

Following parameter optimization, we also tested whether varying the log_2_ ratio thresholds used for calling the events (-2.5 for homozygous deletions, -0.5 for hemizygous deletions, and 0.2 for duplications) yielded better results. However, no other thresholds yielded better results, so we kept the initial thresholds for all analyses of the data from the first GBS library.

*Optimization of the parameters for CNV calling from the GBS data of the combined libraries*

Upon analyzing the data from the two combined libraries using the parameters deemed optimal for the analysis of the first library, we found that this combined analysis did not detect some of the true events that were detected with the data from the first library alone. We concluded from this that the parameters used for the first library did not suit the purposes of calling CNVs from the combined libraries and thus decided to carry a second run of parameter optimization using the data from the two libraries combined to understand where the problem came from and solve this issue. We modified the parameter values relative to the first optimization run, testing the following values: mapq (15, 20, 25, 30, 35), minr (6, 7, 8, 9), segt (5, 5.5, 6, 6.5, 7). One hundred (100) parameter combinations were thus tested. The threshold for declaring homozygous deletions was also modified from -2.5 to -3.1 in the analysis of the combined datasets, as a value of -3.1 was found to be a better cut-off between true and false calls in this dataset. The thresholds for hemizygous deletions and duplications were maintained at -0.5 and 0.2, respectively.

For homozygous deletions, several parameter combinations were able to achieve an optimal result of 31 true positive calls and no false positive calls (Figure S15). Only a few parameter combinations, all stemming from including reads with mapping quality ≥ 15, resulted in a false positive call being made; all other combinations yielded no false positives. Among the optimal parameter combinations, we selected the one with mapq = 20, minr = 7 and segt = 7, as the only difference from the parameters used with the first GBS library was then the segmentation parameter. It followed from this that the main difference in optimal calling parameters between the first library dataset and the combined dataset was that the combined dataset required a higher segmentation parameter for the proper detection of smaller events. This presumably occurred as the higher number of data points (roughly 1.5 times more bins in the combined dataset) resulted in lower standard deviation estimates for the log_2_ ratio values. In summary, the combined dataset allowed two additional aCGH-validated deletions to be detected from the GBS dataset while maintaining zero false positives.

For hemizygous deletions, the best results (14 true positives and 1 false positive) were obtained using the parameter combination mapq = 35, minr = 9, segt = 5 (Figure S16). The addition of the sequencing data from the second library thus resulted in one additional true positive relative to the data from the first library only, while maintaining the same number of false positives (1). Again, it was apparent that hemizygous deletions required more stringent parameters than homozygous deletions to be accurately called from GBS data.

For duplications, results were similar to those obtained for the parameter optimization of the first library. Results were again split between parameter combinations resulting in 10 and 7 true positive calls. A few (8) of the parameter combinations resulted in a single false positive call; all other parameter combinations declared zero false positives. Since the parameter combination that we chose for homozygous deletions (mapq = 20, minr = 7 and segt = 7) was also among the combinations yielding optimal results for duplications, we decided to use this parameter combination for calling duplications.

Overall, although future research should build upon our results to select optimal parameters, every new dataset will likely require slightly different parameters to obtain optimal results. Since not all research projects will have aCGH or WGS data at hand to validate their results, we recommend that researchers implementing similar methods at least look at the log_2_ ratio profiles of their data to verify that all or most visually striking events have been found and that a minimal number of apparently spurious calls is being made.

*Simulating the effect of different sequencing depths on CNV calling from GBS data of the first library*

In order to identify the ideal sequencing depth for detecting CNVs from GBS data, we performed simulations in which we sampled reads from the complete dataset and assessed the performance of our CNV calling pipeline in terms of the number of true positive events called, the number of false positive events called, and the mean size of the true events that were found. We performed 10 replications of 9 different sampling proportions (from 10% to 90% with 10% intervals) using the parameters that had been found optimal for each event type. This neglected the existence of possible interactions between sequencing depth and optimal calling parameters but reflected the fact that not all datasets will go through a thorough parameter optimization assessment in practice.

For homozygous deletions, the number of true events found appeared to reach a plateau at around 50-60 million mapped reads (Figure S17a). This number of reads corresponded roughly to the usable output generated by two Ion Proton chips after bioinformatic processing, quality filtering (mapping quality ≥ 20), and the removal of 11 out of the 96 assessed samples. One interesting result was that the sequencing depth did not appear to influence the number of false positive calls, suggesting that the approach and parameters used are robust to false positives when it comes to calling homozygous deletions. The mean size of the homozygous deletions found dropped rapidly and appeared to stabilize at around 40 million mapped reads, suggesting that any deeper sequencing is unlikely to increase the resolution of the approach (Figure S18). The better performance of the CNV-calling pipeline when using the total number of reads is likely due to the fact that parameters were optimized specifically for this dataset rather than to the increased number of reads.

For hemizygous deletions, it was unclear whether the number of true events found plateaued or not when using the full set of reads, however it clearly appeared that the number of usable reads generated from two Ion Proton chips was not sufficient for extracting the full potential of the library (Figure S17b). The mean size of the true hemizygous deletions called decreased slowly but steadily until approximately 60 million mapped reads, indicating that more small events were found as sequencing depth increased (Figure S18). A noteworthy observation made from the sampling simulation results was that the number of false positives called increased almost linearly with the number of true positives. This indicates that one should be careful when calling hemizygous deletions from GBS data, as a non-negligible proportion of the calls are likely to be false. Until better solutions are found, manual and visual review of hemizygous deletion calls may be the best method for filtering out false calls. This issue might be solved by adjusting the generic segmentation approach used here to make it better tailored to our particular problem.

For duplications, the number of true events found plateaued surprisingly rapidly at the predicted output of a single Ion Proton chip (Figure S17c). As a consequence, the mean size of the duplications found also stabilized rapidly as sequencing depth increased (Figure S18). Duplication calls were highly robust, as almost no false duplications were called among datasets with at least as many reads mapped as can be generated by a single Ion Proton chip.

*Simulating the effect different sequencing depths have on CNV calling from GBS data of the two combined libraries*

We wanted to assess whether CNV calling from GBS data benefited more from deeper sequencing or from the addition of a different library. Since we had only carried out one sequencing run from our second GBS library, it was not really possible to test different combinations of sequencing depths from the two libraries. (We could have sampled from the reads of the single run of the second library, but it would not have been very informative.) Instead, we decided to replicate the sampling experiment done for the data from the first library, but to add the full data from the second library at every iteration. Our objective in doing this was to evaluate the effect of sequencing depth of a given library *given that data was already available from another library*. We thus performed the same 10 replications at 9 different sampling proportions of the first library and added the reads of the second library before processing the resulting read counts through our pipeline. For every iteration, we naively used the parameters that were determined to be optimal for the combined dataset (see above). The number of true positive and false positive calls relative to the aCGH data for every event type were tallied at every iteration without considering the size of the events detected for these sampling simulations.

The number of homozygous deletions detected appeared to increase slowly but steadily as the number of reads increased, although the benefits of increased sequencing were arguably negligible above 40 million reads sampled from the first GBS library (Figure S19a). Interestingly, the number of true homozygous deletions seemed to be slightly higher when combining a single sequencing run of the second library and the equivalent of a single sequencing run of the first library, than when sampling the equivalent of two sequencing runs from the first library only; it thus appears that the marginal benefits of a different library are higher than those of deeper sequencing of the same library. Based on these results, we conclude that two sequencing runs of one library and a single run of another library, or even only a single sequencing run of each library, would satisfy the needs of homozygous deletion calling. As for hemizygous deletions, the number of events accurately called appeared to plateau at higher sequencing depth (around the equivalent of two chips sampled from the first library; Figure S19b). The results from duplication calling were surprising in showing that the number of duplications accurately called was already optimal even at the lowest sequencing depths (Figure S19c), showing again that these events can be robustly called even with minimal sequencing depth. This is likely due to the large size of the duplication events, which are on average 9.0 times larger than homozygous deletions and 2.8 times larger than hemizygous deletions, making duplications more reliably discoverable at lower sequencing depths.

*Validation of the GBS approach using PCR and ddPCR – detailed methods and results*

To further assess the accuracy of the calling of CNVs based on GBS data, we adopted a PCR approach focused on the most challenging cases, those resting on the least information. Also, since aCGH provides only a relative measure of copy number (mutant versus control), we wanted to assess some of the events detected by the GBS approach using methods that would provide an absolute measure of copy number. To these ends, we selected the smallest events of each type (duplications, hemizygous deletions, and homozygous deletions) among samples not assessed by aCGH and assessed them by PCR and/or ddPCR (droplet digital PCR) to provide an absolute assessment of copy number of events which are near the detection threshold. In this context, the “smallest” events are those with the least number of supporting bins rather than the smallest physical size, as confidence in CNV calls generated using the GBS approach depends more on the number of supporting bins than on the actual physical size of the event. We concentrated our efforts on events which had not been validated by aCGH in order to provide independent validation results for a different set of samples. We thus selected the smallest four duplications, eight hemizygous deletions, and 14 homozygous deletions for assessment with PCR and/or ddPCR; the number of events selected from each type reflected the relative frequency of these types of events in our dataset. In addition, we sought to validate two homozygous deletions which were each found in two replicates of a control line (M92-220-Long R6-1 and M92-220-Long R6-2) by using M92-220-Long R6-1 DNA. The resulting set of 28 events validated by PCR and ddPCR and associated results are summarized in Additional file 5.

PCR primer design was automated using an in-house pipeline which used the command-line version of Primer3 (Untergasser et al. 2012) for generating primers (see Additional file 6 for the Primer3 parameters that were used) and Exonerate (Slater and Birney 2005) for aligning primers to the *Glycine max* reference genome assembly version 2 (supplemented with mitochondrial and chloroplast genome as described in the main manuscript) in order to check for the specificity of primer pairs. To define the regions in which to design primer pairs, we visually assessed the boundaries of the *Ape*KI restriction fragments that supported each event and used the sequences in these regions as input to Primer3; this way, we could use the GBS data generated as part of this project in order to filter out the candidate primer pairs which contained SNPs relative to the reference genome. Up to 200 primer pairs which met specific criteria (see Additional file 6) were generated for each candidate region using Primer3. The primers obtained were then input to Exonerate for alignment. Primer pairs meeting the following requirements were considered as potential amplification products following alignment with Exonerate: (1) both primers have an Exonerate score > 80; (2) any of the combinations of the two primers (forward-reverse, forward-forward, or reverse-reverse) align to the reference genome at a distance of less than 5 kb from one another; (3) the number of nucleotide differences between any primer in a matching pair and the reference sequence is < 6; (4) the number of differences between any primer in a matching pair and the reference is < 3 in any of the five last 3’ nucleotides. Primer pairs for which a single amplification product (i.e. the intended target) was predicted following alignment were considered as candidates for use in PCR and/or ddPCR. Up to two (when possible) primer pairs per event were selected and checked for specificity relative to the *Glycine max* genome on the Primer-BLAST online tool (Ye et al. 2012) before ordering primers. The two primer pairs selected for each event were constrained to target different restriction fragments.

The first phase of primer design described above yielded a total of 32 primer pairs targeting 19 distinct events (out of a total of 28). To obtain at least one primer pair for the remaining events, we applied more relaxed parameters for primer design by neglecting potential secondary amplicons that (1) were larger than 1 kb (which should be distinguishable on electrophoresis), (2) resulted from primers having 5 or more differences with the reference sequence, and (3) resulted from primers having 2 or more differences with the reference sequence in any of the five last 3’ nucleotides. The primers designed following these steps were again checked for specificity with Primer-BLAST before ordering. At that point, 39 primer pairs targeting a total of 22 distinct events had been designed.

To provide primer pairs for the remaining events, we allowed for primer pairs to be designed within sequences located at distances up to 5 kb from the ends of a restriction fragment while being within the boundaries of the putative CNV. We could not filter the primers designed at this step for SNPs because the target sequences were then outside the boundaries of the sequences that had been captured by GBS. Primers were designed following the same steps as described above using the stringent set of parameters for the identification of potential secondary targets. Following this last step, we obtained a total of 52 primer pairs which could target all 28 events. The putative hemizygous deletions on individuals FNMN0013 and FNMN0015 were targeted by the same single primer pair (primer pair ID: BP) as they overlapped each other.

Four potential control primer pairs that could serve as single-copy reference controls in ddPCR were also designed. To be useful, these primers had to amplify a single locus in every mutant or control line. To identify such potential primer pairs, we first filtered our dataset of read counts at a mapping quality ≥ 35 and kept only bins in which at least five but less than 100 reads per individual were observed. These bins were further filtered to remove those in which any sample had a log2 ratio ≤ -0.8 or ≥ 0.5. Restriction fragment coordinates in the remaining bins were identified visually and the corresponding sequences were subjected to primer design with primer3 and specificity assessment with Exonerate as described above. From the candidate primer pairs, four pairs (labeled BV, BW, BX and BY) were randomly selected and ordered, although only BY (which indeed amplified a single locus) was the only one effectively used in ddPCR assays. All primers were ordered from Integrated DNA Technologies, Inc. (Coralville, IA, USA). The primers that were ordered are listed along with their intended target coordinates and predicted amplicon sizes (range from 75 bp to 173 bp) in Additional file 7.

All primer pairs were tested on control M92-220 wild-type DNA (sample M92-220-Long V3-2) to check that they resulted in a single band of the expected length following PCR on gel electrophoresis. All PCR reactions (except ddPCR reactions – see below for details) were performed in the same conditions in a 20-µl volume which comprised 2 µl of 2 mM dNTPs, 2 µl of 10X ThermoPol reaction buffer (New England Biolabs inc., Ipswich, MA, USA), 1 µl of DNA at an estimated concentration of 20 ng/µl (measured by Nanodrop), 1 µl of each primer at a concentration of 10 µM (one or two pairs depending on the experiment), 1 µl of *Taq* DNA Polymerase (New England Biolabs inc., Ipswich, MA, USA) and ddH_2_O to complete the volume to 20 µl. The PCRs were run on a Biometra T Professional gradient thermocycler in the following conditions: 2 min at 94°C, followed by 36 cycles of 45 s at 94°C, 45 s at 60°C, and 1:30 min at 72°C, and a final elongation step of 5 min at 72°C. Migration of the PCR products on a 3% agarose gel showed that all but three of the primer pairs (AG, AJ and BW) yielded a single band of the expected length. The primer pairs AG and BW was not used for subsequent analyses, but AJ could still be used for presence/absence diagnosis because its secondary amplicon was much larger (> 400 bp) and could be easily distinguished on gel electrophoresis.

All putative homozygous deletions were first assessed by regular PCR for presence/absence of an amplification product. For every putative deletion, two reactions were performed: one with control wild-type M92-220 DNA (sample M92-220-Long V3-2) and mutant DNA. Each reaction was performed with both a pair of diagnostic primers and another pair of control primers that was expected to amplify in both the control and mutant DNA and was selected so that both bands (the diagnostic and the control band) would resolve on electrophoresis. PCR products were run on a QIAxcel Advanced capillary electrophoresis system (Qiagen, Hilden, Germany) for assessment of the presence or absence of PCR products. A presence/absence call for the diagnostic band was made only if both bands were observed in the control sample and the control band amplified in the mutant sample. In some cases, other bands which presumably resulted from an interaction between the two primer pairs were observed, but a call was still made if the conditions above were met.

Following the results of the PCR amplification (summarized in Table S1 and Figures S20 through S35), five of the 16 homozygous deletions could be readily validated as none of their target primer pairs resulted in amplification in the mutant. Another three events were unusual in that amplification occurred for one of the primer pairs but not for the other. Finally, amplification occurred for all primer pairs for the remaining 8 putative deletions. Whenever amplification of the diagnostic band occurred in a mutant, the two primer pairs targeting that deletion were subjected to ddPCR to determine whether amplification might have occurred due to contamination of the mutant DNA or the amplification of secondary targets. In the first case (contamination), the ddPCR results would indicate residual amounts of wild-type DNA in the mutant, whereas in the second case (secondary targets), ddPCR results would indicate that the primer pair used amplified more than one locus in the control sample.

Droplet digital PCR (ddPCR) was conducted to assess all duplications and hemizygous deletions as well as putative homozygous deletions that unexpectedly yielded an amplification product on PCR followed by capillary electrophoresis. ddPCR essentially provides an assessment of the number of copies of DNA templates amplified by a given primer pair in a sample. We conducted a total of 130 ddPCR assays that could be grouped in four categories. The first category of assays comprised 38 assays which assessed the copy number of templates amplified by 37 different primer pairs (36 diagnostic pairs plus two assays using the reference pair BY that amplified a single locus in the genome) on control M92-220 wild-type DNA; these assays were used to determine the number of loci amplified by a given primer pair in a wild-type background. The second category of assays comprised 18 assays which assessed the DNA concentration in each mutant sample using a common reference primer pair (pair BY) that was confirmed to amplify a single locus by the results obtained on the control DNA; the value determined for each mutant could be used to express the results obtained for each primer pair in a mutant as an absolute number of copies as these assays provided the expected values for two copies of the template DNA in each mutant. The third category of assays comprised 37 assays which evaluated the copy number of the templates amplified by each diagnostic primer pair in its target mutant; these values were used to assess whether the region was deleted (hemizygous or homozygous) or duplicated in the mutant. Finally, a last group of 37 assays served as a negative control of each primer pair by testing these without input DNA, effectively confirming that the results obtained for all primer pairs were due to actual amplification of the target DNA and not to primer dimers.

We prepared each ddPCR assay by mixing 4 µl of target DNA at 1 ng/µl (measured by a Qubit dsDNA HS assay, Invitrogen, Waltham, MA, USA) with 1 µl of each primer at a concentration of 2 µM, 4 µl of ddH_2_O and 10 µl of QX200 ddPCR EvaGreen Supermix (Bio-Rad Laboratories, Hercules, CA, USA). This 20-µl mix was then used along with 60 µl of QX200 Droplet Generation Oil for EvaGreen (Bio-Rad Laboratories) for generating ~20,000 droplets in a Bio-Rad QX200 Droplet Generator. The plate was then sealed with a Bio-Rad PX1 PCR Plate Sealer before running the PCR on a Bio-Rad C1000 Touch Thermal Cycler in the following conditions: 95°C for 5 min, followed by 50 cycles of 95°C for 30 s, 60°C for 1 min and 72°C for 30 s, followed by two steps of 4°C for 5 min and 90°C for 5 min. Ramp rates of 2°C/s were used for all temperature transitions, except for the transition from 95°C to 60°C for which a ramp rate of 2.5°C/s was used. After PCR, droplet fluorescence was measured using a Bio-Rad QX200 Droplet Reader coupled with the QuantaSoft Software (Bio-Rad). All assay results were individually verified to make sure that the fluorescence threshold automatically set by the software between positive and negative droplets was appropriate, and manually adjusted in some cases.

The concentration (in number of template copies per µl) values output by the QuantSoft software (see Additional file 8) were processed into an estimated number of copies per genome by dividing the concentration obtained for each assay by the concentration obtained for the corresponding sample with the BY reference primer pair, times two (because of the diploid status of the soybean genome). Twenty-two (22) out of the 36 diagnostic primer pairs were found to amplify a single locus and could thus provide a robust and reliable assessment of copy number in mutant samples. The copy number for the 14 other primer pairs ranged from 2.7 to 104.6. Most values of copy number were close to integer multiples of two and likely represented primer pairs amplifying different templates with equally high efficiency, whereas in a few cases copy number values appeared more random and likely represented the fact that one or several secondary templates were amplified with reduced efficiency relative to the intended target. Despite this, meaningful information could often be obtained from these primers, but the fact that they amplified several loci made it harder to reach robust conclusions. Copy number values for both control DNA and mutant DNA are listed for every primer pair assessed by ddPCR in Additional file 4 and visually represented in Figure 6 in the main manuscript.

Following ddPCR assays, two out of the four duplications could be validated, whereas one was found to be a false positive call and the remaining one could not be assessed because the two primer pairs targeting it amplified a large number of loci (> 5) which prevented validation. Out of the eight hemizygous deletions assessed by ddPCR, five could be validated, while two putative hemizygous deletions turned out to be homozygous deletions (the ones in FNMN0017 and FNMN0051) and the remaining one was found to be a false positive call. Evidence that the putative hemizygous deletions on FNMN0017 and FNMN0051 were in fact homozygous deletions was provided by the fact that in both cases, one of the primer pairs yielded a copy number of 0 in the mutant and 2 in the control, whereas the other primer pair yielded a copy number of 2 in the mutant and 4 in the control. Finally, primer pairs targeting 11 homozygous deletions were also assessed by ddPCR. The three events (the ones found in FNMN0012, FNMN0086, and chromosome 2 of M92-220-Long R6-1) for which PCR amplification occurred for one of the primer pairs but not for the other were confirmed to be homozygous deletions by the ddPCR results. Indeed, ddPCR results confirmed the absence of amplification for one of the primer pairs, while for the primer pair that resulted in the observation of a PCR product, ddPCR results indicated that these primers did amplify at least one locus in the mutant, but consistently resulted in a lower number of copies than in the control line. The two primer pairs targeting the putative homozygous deletion of FNMN0057 were shown to amplify two additional copies in the control line relative to the mutant, supporting the existence of a homozygous deletion at that locus although no definite conclusion could be reached in that case. Two events (the one on chromosome 9 of M92-220-Long R6-1 and the one on chromosome 6 of FNMN0064) were targeted by primer pairs that amplified so many loci that no conclusion could be reached in these cases. Finally, five homozygous deletions (the four putative homozygous deletions on FNMN0038 and the one on FNMN0017) were confirmed to be false positive calls as the ddPCR provided evidence of the amplification of approximately two copies of the target regions in both the control and mutant lines.

**Literature cited**

Bradbury PJ, Zhang Z, Kroon DE, Casstevens TM, Ramdoss Y, and Buckler ES. 2007. TASSEL: software for association mapping of complex traits in diverse samples. Bioinformatics 23:2633-2635.

Danecek P, et al. 2011. The variant call format and VCFtools. Bioinformatics 27:2156-2158.

Fearnhead P, and Rigaill G. 2018. Changepoint detection in the presence of outliers. Journal of the American Statistical Association. doi:10.1080/01621459.2017.1385466.

Slater GS, and Birney E. 2005. [Automated generation of heuristics for biological sequence comparison](http://europepmc.org/abstract/MED/15713233). BMC Bioinformatics 6(31).

Torkamaneh D, Laroche J, Bastien M, Abed A, and Belzile F. 2017. Fast-GBS: a new pipeline for the efficient and highly accurate calling of SNPs from genotyping-by-sequencing data. BMC Bioinformatics 18(5).

Untergasser A, Cutcutache I, Koressaar T, Ye J, Faircloth BC, Remm M, and Rozen SG. 2012.

Primer3--new capabilities and interfaces. Nucleic Acids Res 40(15):e115.

Ye J, Coulouris G, Zaretskaya I, Cutcutache I, Rozen S, and Madden T. 2012. Primer-BLAST: A tool to design target-specific primers for polymerase chain reaction. BMC Bioinformatics 13(134).

**Supplementary table**

| **Table S1**: Results of the validation of putative homozygous deletions by presence/absence of the amplification product after PCR | | | | | | |
| --- | --- | --- | --- | --- | --- | --- |
| Sample | Chromosome | Start (bp) | End (bp) | Primer pair 1† | Primer pair 2† | Assessed by ddPCR‡ |
| FNMN0012 | Chr14 | 14,436,000 | 14,508,000 | yes | no | yes |
| FNMN0017 | Chr08 | 9,450,000 | 9,458,000 | yes | yes | yes |
| FNMN0037 | Chr10 | 6,034,000 | 6,069,000 | no | N.D. | no |
| FNMN0038 | Chr01 | 47,907,000 | 47,936,000 | yes | yes | yes |
| FNMN0038 | Chr09 | 39,115,000 | 39,202,000 | yes | yes | yes |
| FNMN0038 | Chr13 | 23,112,000 | 23,126,000 | yes | yes | yes |
| FNMN0038 | Chr16 | 33,120,000 | 33,137,000 | yes | yes | yes |
| FNMN0049 | Chr14 | 24,802,000 | 24,913,000 | no | N/A | no |
| FNMN0051 | Chr01 | 46,717,000 | 46,729,000 | no | no | no |
| FNMN0055 | Chr20 | 40,774,000 | 40,782,000 | no | N/A | no |
| FNMN0057 | Chr05 | 29,564,000 | 29,766,000 | yes | N.D. | yes |
| FNMN0064 | Chr06 | 47,068,000 | 47,086,000 | yes | yes | yes |
| FNMN0064 | Chr20 | 35,731,000 | 35,750,000 | N.D. | no | no |
| FNMN0086 | Chr17 | 31,528,000 | 31,674,000 | no | yes | yes |
| M92-220-Long_R6-1 | Chr02 | 21,966,000 | 22,879,000 | yes | no | yes |
| M92-220-Long_R6-1 | Chr09 | 13,714,000 | 14,037,000 | yes | yes | yes |
| †"yes" indicates that an amplification product was observed; "no" indicates that no amplification product was observed; "N.D." stands for “not determined” and means that the results did not allow a conclusion for that primer pair; "N/A" means that there was no second primer pair available for that event. Results are based on the electropherograms presented in figures S20 through S35. The first and second primer pairs refer to those listed in Additional file 5. | | | | | | |
| ‡ All events for which at least one primer pair produced an amplification product in the mutant had all their primer pairs assessed by ddPCR. | | | | | | |

**Figures and figure captions**

**
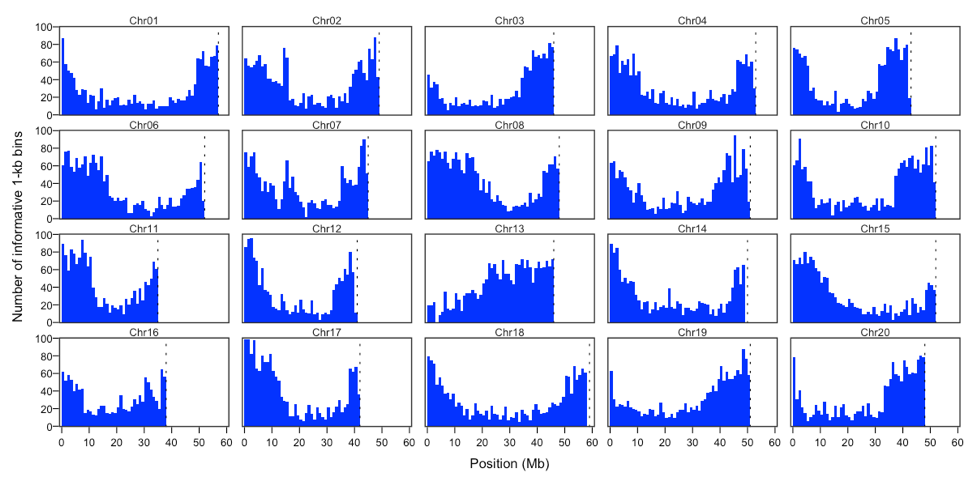
**

**Figure S1:** Number of informative 1-kb bins per Mb of the soybean reference genome. Vertical dotted lines mark the end position of each chromosome. The higher resolution of the approach in euchromatic regions as compared to pericentromeric (heterochromatic) regions is clearly apparent. The dataset represented here was used for calling homozygous deletions from the first GBS library (total number of bins = 32,741).

**
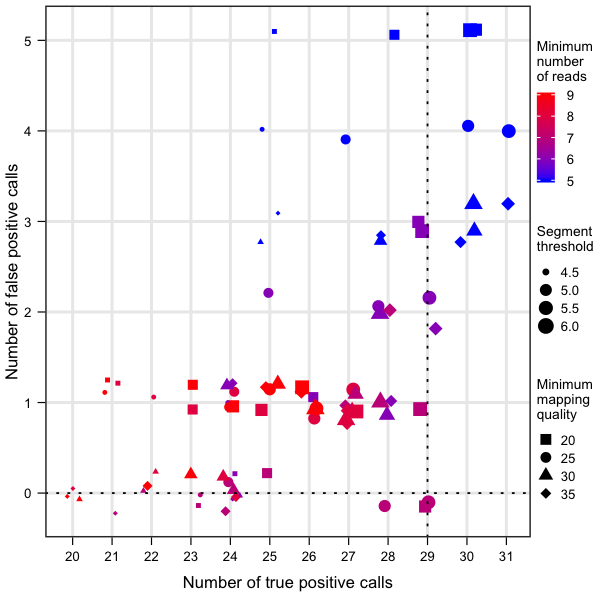
**

**Figure S2:** Number of true positive and false positive homozygous deletions called from the GBS data of the first library using different combinations of minimum number of reads per bin, segmentation threshold, and minimum mapping quality. Deletions are labeled as true positives if they overlap with a deletion found by the aCGH array and considered as false positives otherwise. Points were jittered along both the x and y directions to allow for points mapping to the same coordinates to be visualized. The intersection of the two dotted lines indicates the optimal results.

**
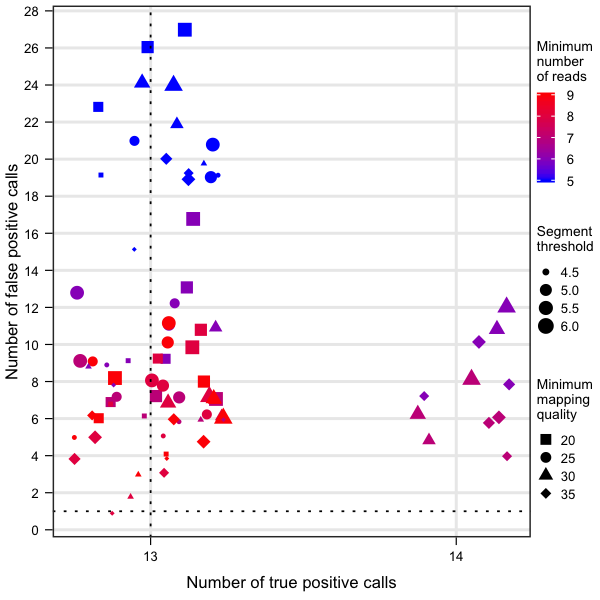
**

**Figure S3:** Number of true positive and false positive hemizygous deletions called from the GBS data of the first library using different combinations of minimum number of reads per bin, segmentation threshold, and minimum mapping quality. Deletions are labeled as true positives if they overlap with a deletion found by the aCGH array and considered as false positives otherwise. Points were jittered along both the x and y directions to allow for points mapping to the same coordinates to be visualized. The intersection of the two dotted lines indicates the optimal results.

**
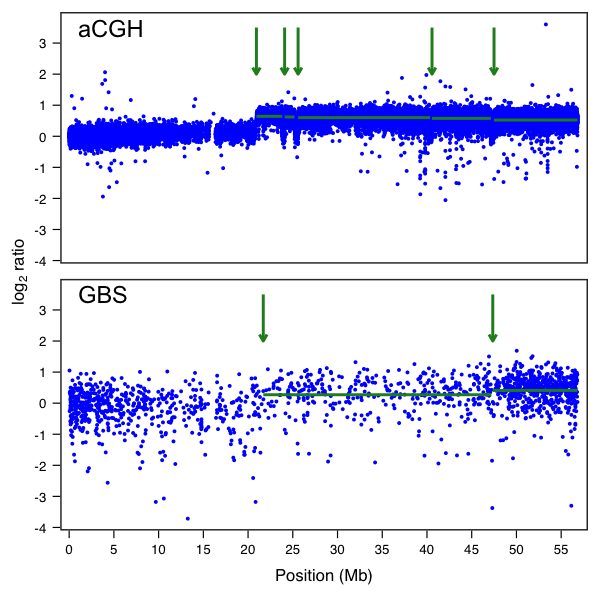
**

**Figure S4:** aCGH and GBS log_2_ ratio profiles of chromosome 1 of FNMN0077. These data show a duplication within the heterochromatin called by GBS that is revealed by the aCGH data to likely represent four different duplications. The GBS log_2_ ratio data plotted are those generated from the dataset used to called homozygous deletions and duplications with the first library. Arrows indicate the starting points of the duplications and solid horizontal lines indicate the span (x-axis) and mean log_2_ ratio (y-axis) of the segment.

**
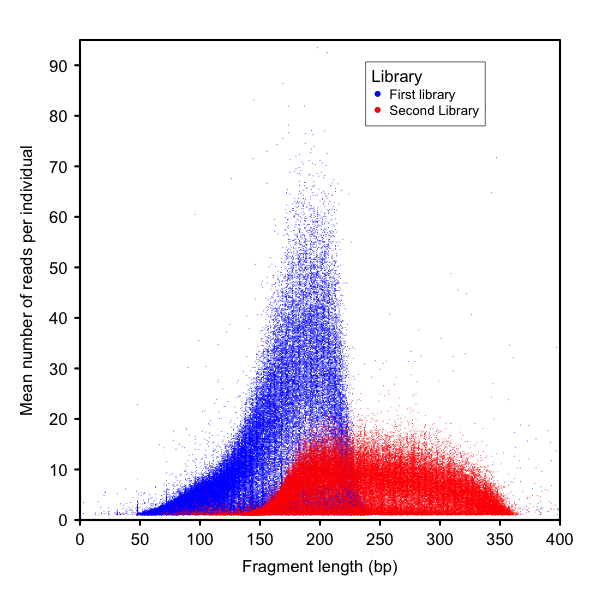
**

**Figure S5:** Mean number of reads observed per restriction fragment as a function of the length of the fragment. The length of the restriction fragment from which a read comes was determined by mapping it to fragments generated *in silico* from the soybean Williams 82 reference genome. Visualisation was restricted to restriction fragments that were observed at least once per individual on average. Reads mapping to restriction fragments longer than 400 bp were excluded as these are likely to result from restriction site polymorphisms between the Williams 82 reference and the M92-220 cultivar used for generating this fast neutron population.


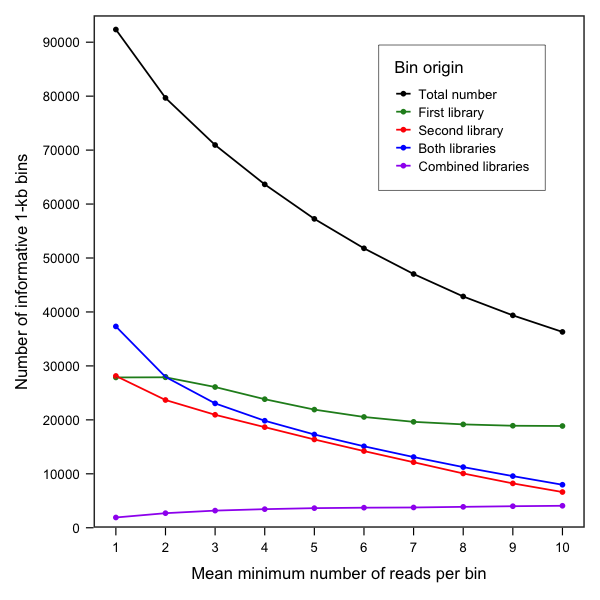


**Figure S6:** Number of informative 1-kb bins contributed by different GBS libraries at different thresholds of mean number of reads per individual for a bin to be considered informative. *Total number* refers to the number of bins kept after merging the two datasets and then filtering the bins. *First library* and *second library* correspond to bins uniquely found in each of the first and second libraries, respectively, at a given threshold. *Both libraries* refers to the number of bins found to be informative in both libraries even without them being combined. *Combined libraries* refers to the number of bins that emerged as informative only when the reads from the two libraries were combined. Note that the data of the first library comes from three different sequencing runs whereas the data of the second library comes from a single sequencing run, which likely explains the higher number of informative bins obtained from the first library.


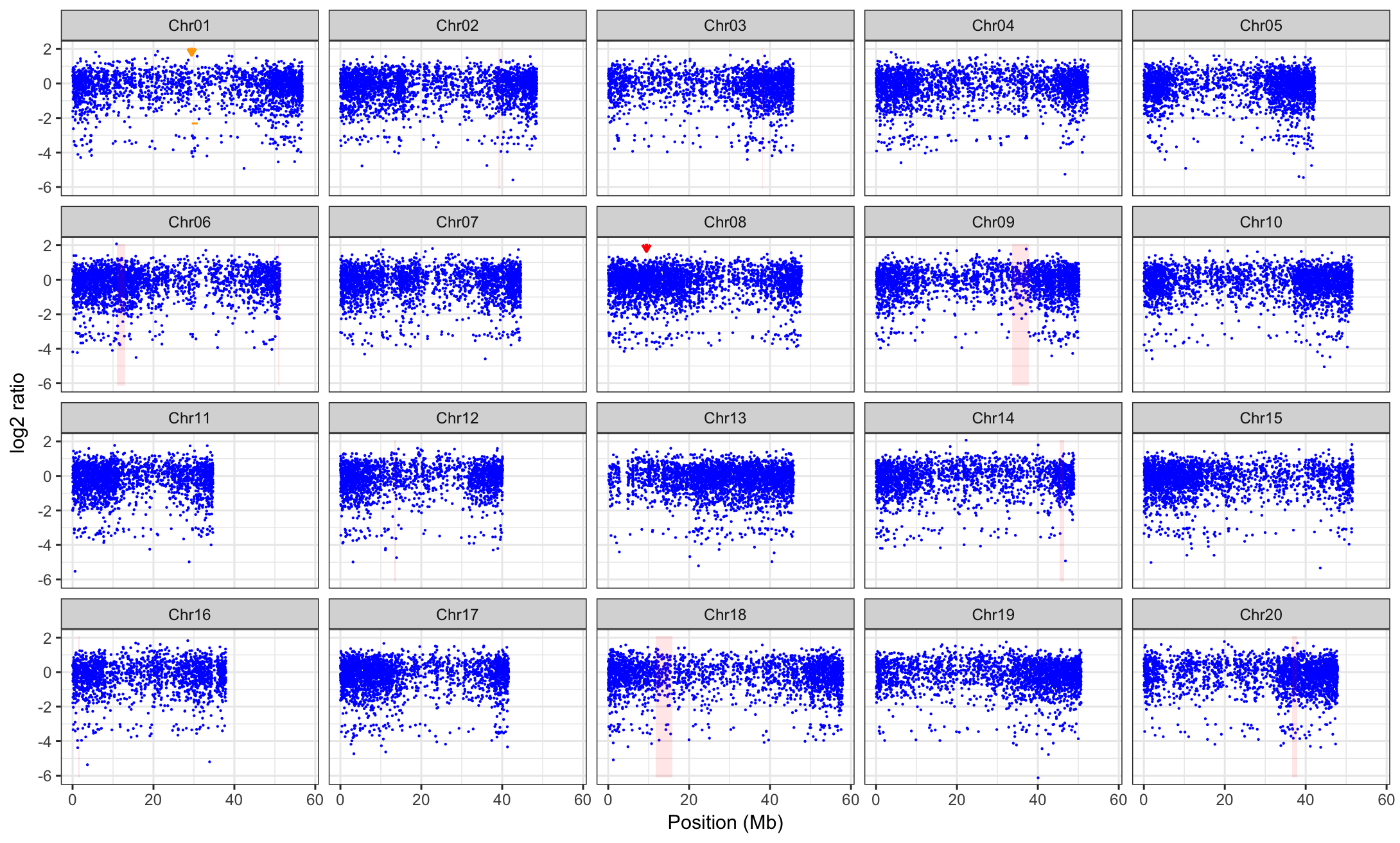


**Figure S7:** Genome-wide log_2_ ratio profiles obtained for individual FNMN0017 by combining the datasets from the two size-selection libraries. The orange arrow on chromosome 1 and the red arrow on chromosome 8 denote a hemizygous deletion and a homozygous deletion, respectively. The results of the ddPCR assay showed that the putative hemizygous deletion was in fact a homozygous deletion, whereas the putative homozygous deletion was in fact a false positive call. The lower coverage in this individual relative to the rest of the dataset caused the grouping of some data points at lower log_2_ ratio values and made this individual more prone to spurious CNV calls. Areas shaded in red denote regions of intracultivar heterogeneity.


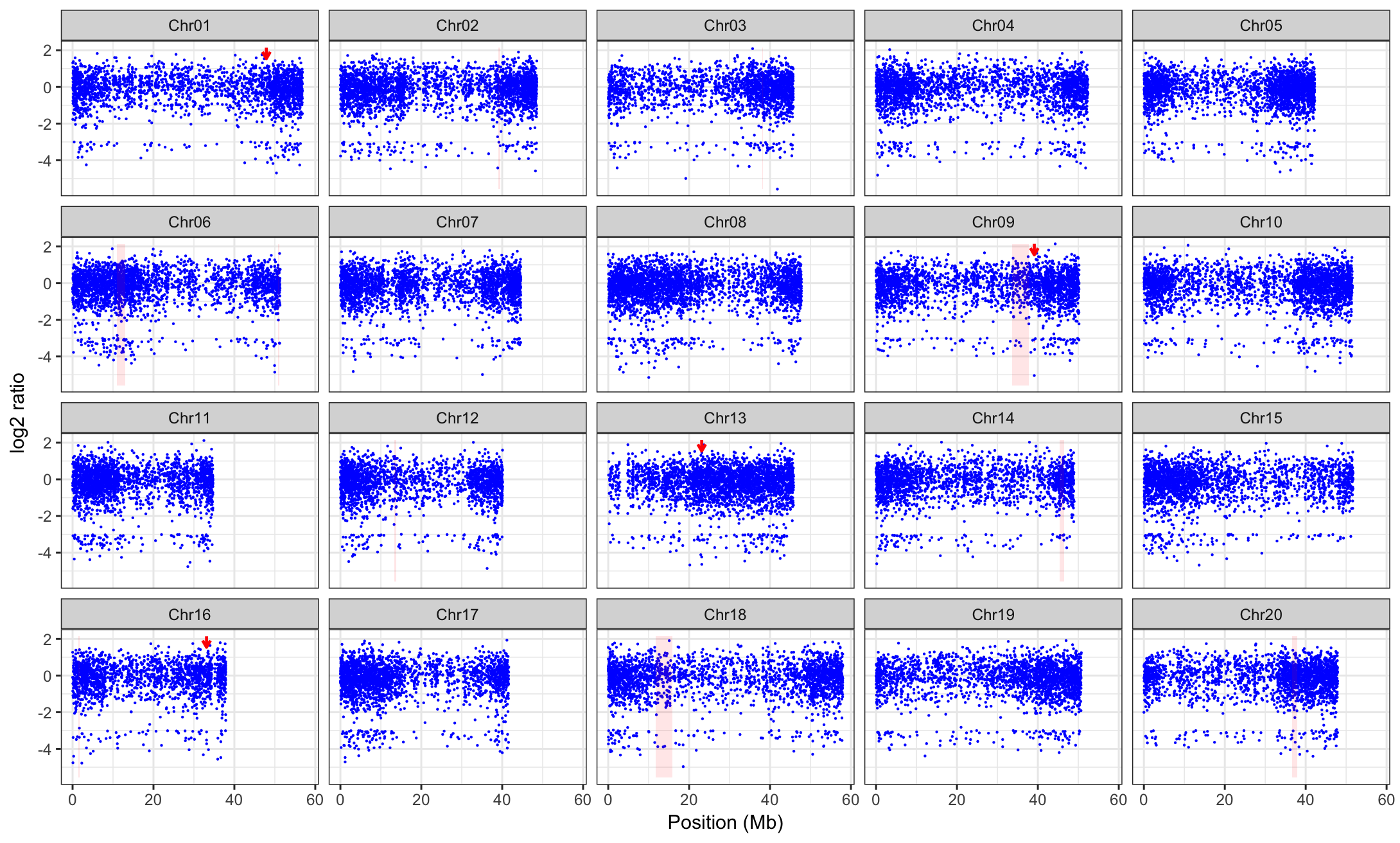


**Figure S8:** Genome-wide log_2_ ratio profiles obtained for individual FNMN0038 by combining the datasets from the two size-selection libraries. The red arrows on chromosomes 1, 9, 13 and 16 denote homozygous deletions. The results of the PCR and ddPCR assays showed that all the homozygous deletions called in this sample were false positives. The lower coverage in this individual relative to the rest of the dataset caused the grouping of some data points at lower log_2_ ratio values and made this individual more prone to spurious CNV calls. Areas shaded in red denote regions of intracultivar heterogeneity.


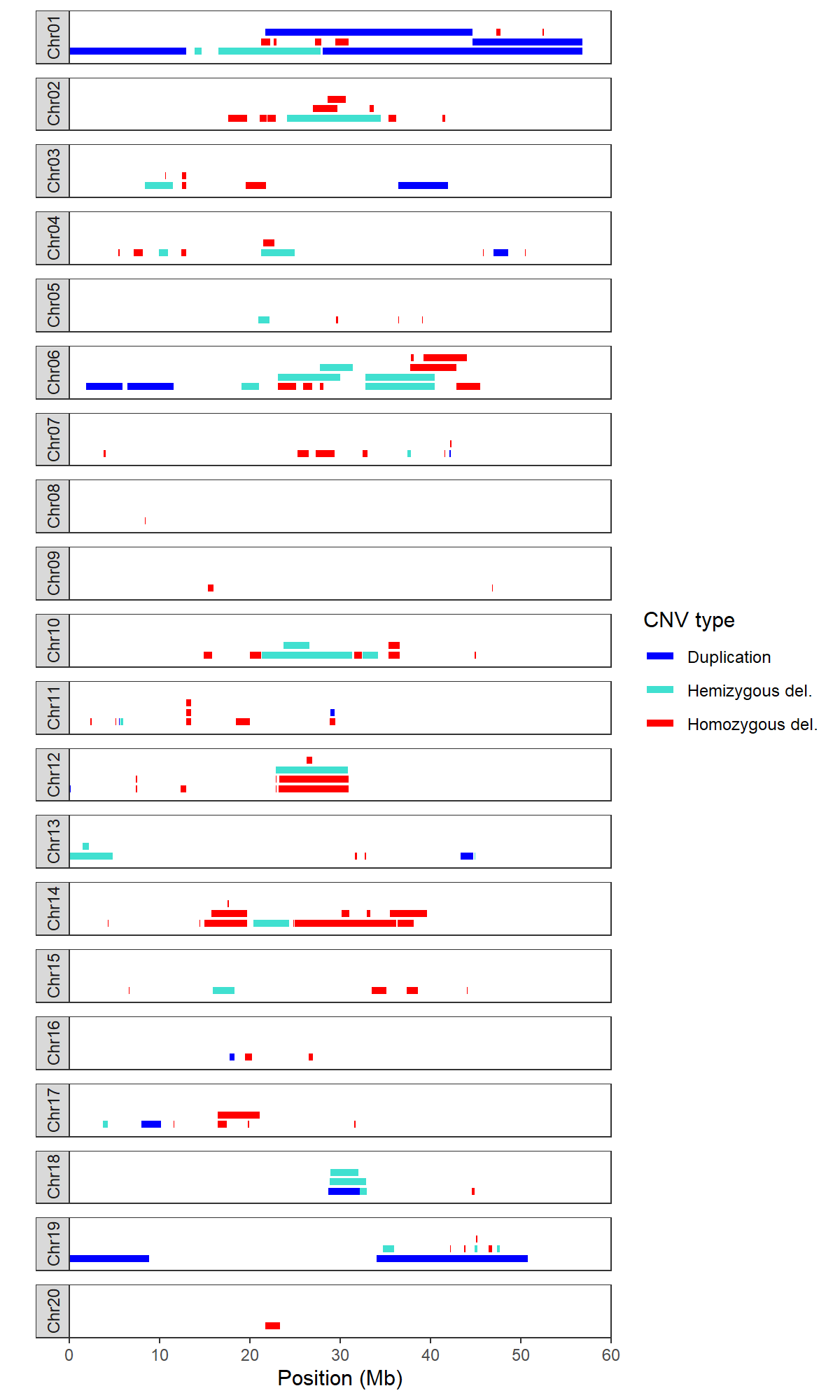


**Figure S9:** Genome-wide location of the events found by the dataset of the two combined GBS libraries among the set of 79 fast neutron mutants assessed. Each rectangle represented a distinct event. The dataset represented here was updated to take the results of the PCR and ddPCR assays into account, i.e., false positive calls were removed and hemizygous deletions that were in fact homozygous deletions were considered as such.

**
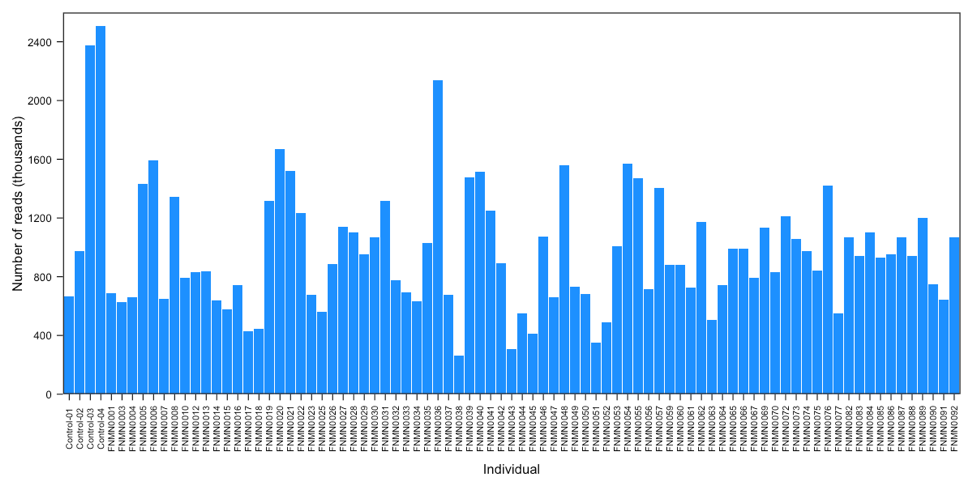
**

**Figure S10:** Number of mapped reads with mapping quality ≥ 20 for the four M92-220 wild-type controls and 81 fast neutron individuals used for CNV calling from GBS data of the first library.


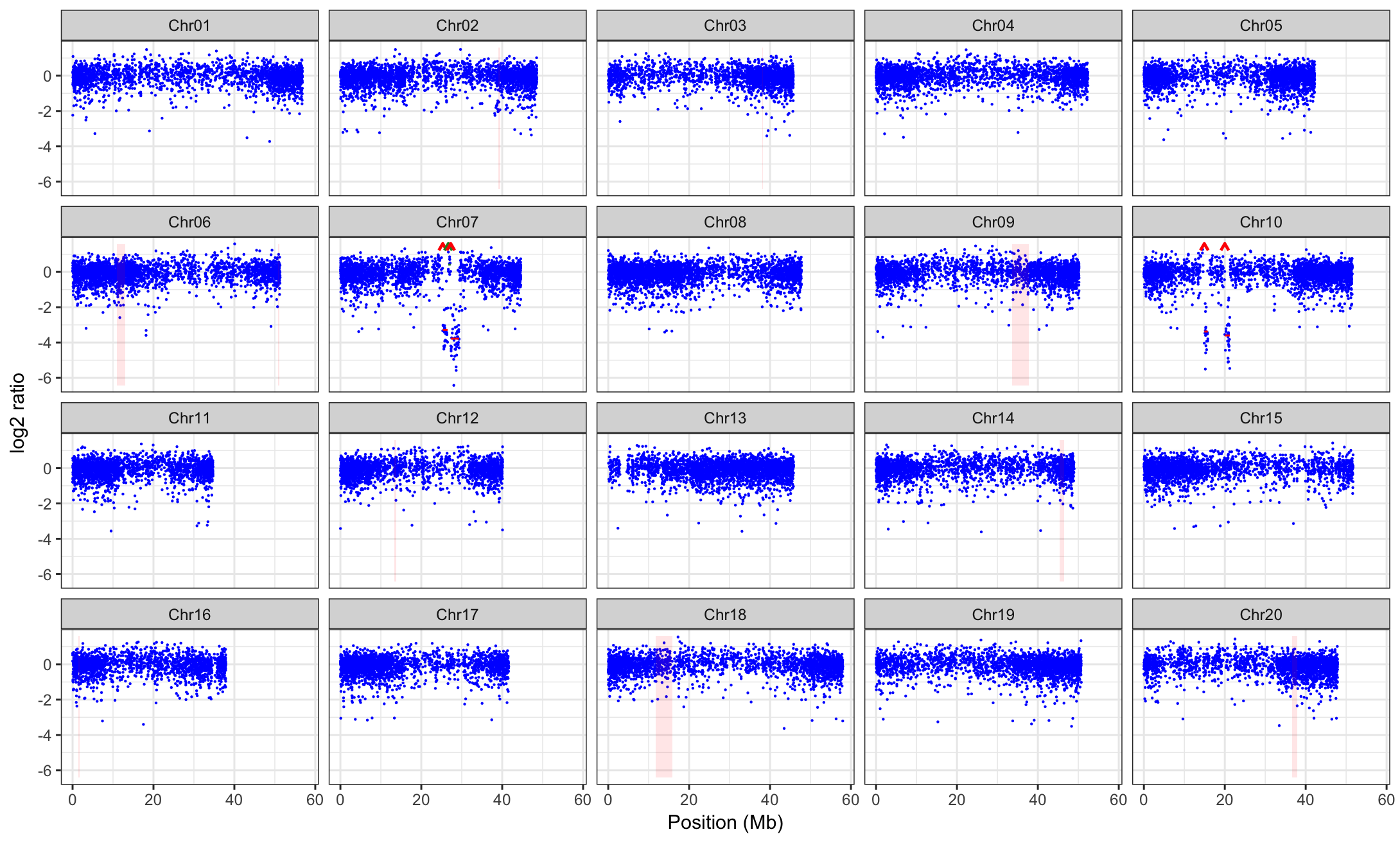


**Figure S11:** Genome-wide log_2_ ratio profiles obtained for individual FNMN0032 by combining the datasets from the two size-selection libraries. The red arrows on chromosomes 7 and 10 denote homozygous deletions whereas the green arrow on chromosome 7 (located between the two red arrows) represents a putative duplication. The results of the ddPCR assays showed that the duplication on chromosome 7 was a false positive call. The location of this section of the chromosome between two homozygous deletions caused it to be set aside as its own segment, which happened to have a mean log_2_ ratio value greater than the duplication threshold. Areas shaded in red denote regions of intracultivar heterogeneity.


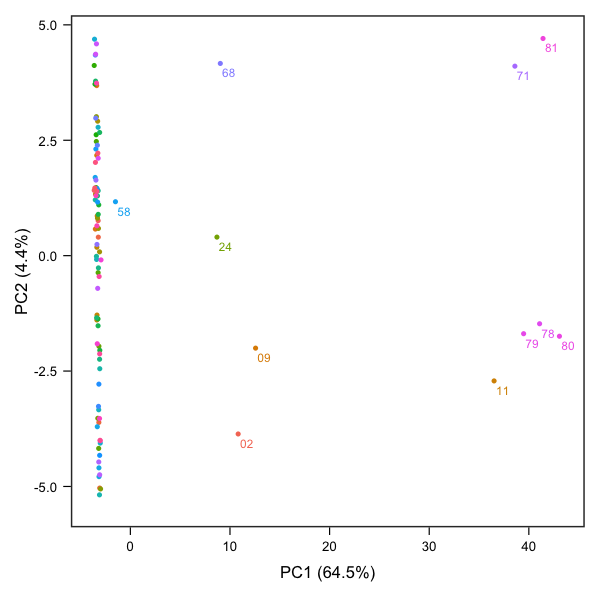


**Figure S12:** Principal component analysis of the SNP data of 92 fast neutron individuals and four wild-type M92-220 controls. The 11 individuals whose SNP profiles clearly differed from the others were labeled with their IDs.

**
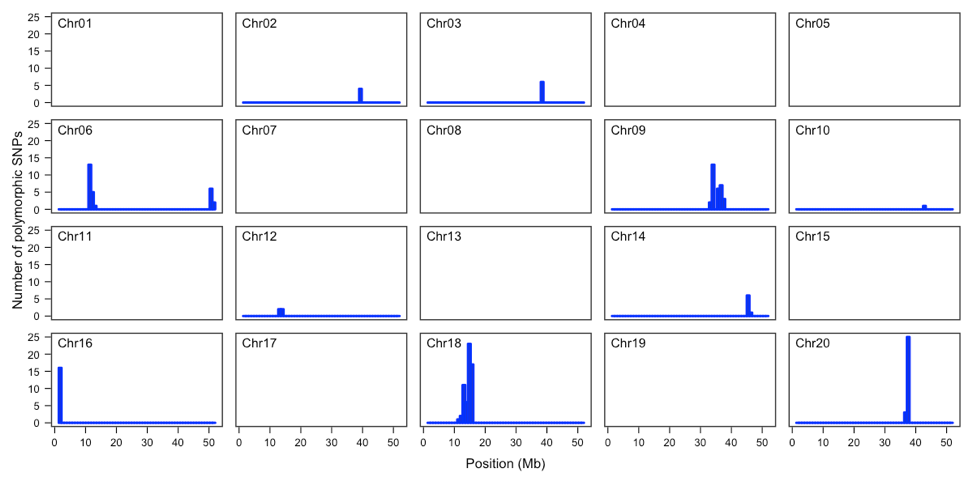
**

**Figure S13:** Physical positions of the polymorphic SNPs among the set of 81 fast neutron individuals and four controls retained for CNV calling from GBS data. Except for the single SNP on chromosome 10, all polymorphic SNPs were used to define so-called heterogeneous regions from which CNV calls were filtered out both in aCGH and GBS datasets.

**
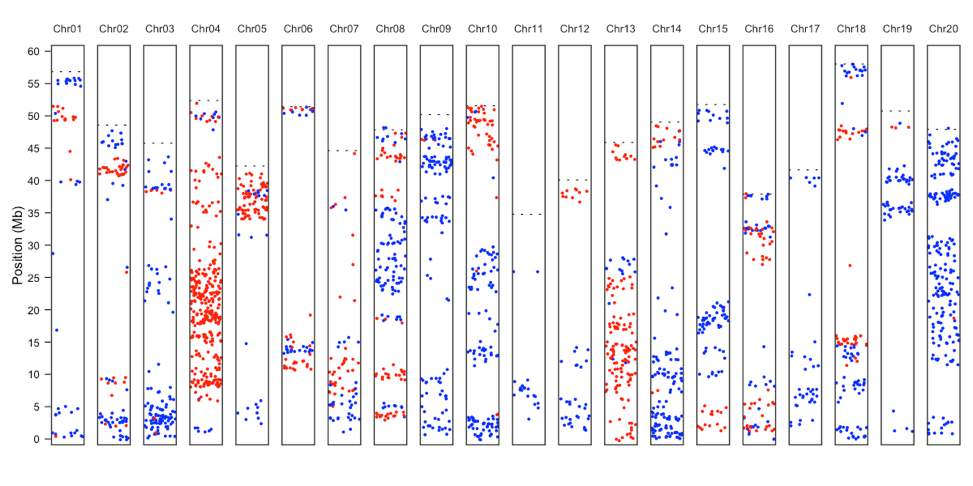
**

**Figure S14:** Physical positions of the concordant (blue) and discordant (red) SNP calls between fast neutron individual FNMN0009 and one of the wild-type M92-220 controls. The results show that discordant calls tend to cluster in given genomic regions, consistent with the interpretation that these polymorphisms must have been introduced from other germplasm. The dotted horizontal lines mark the end position of each chromosome. Points were jittered along the x-axis to allow for easier visualization.

**
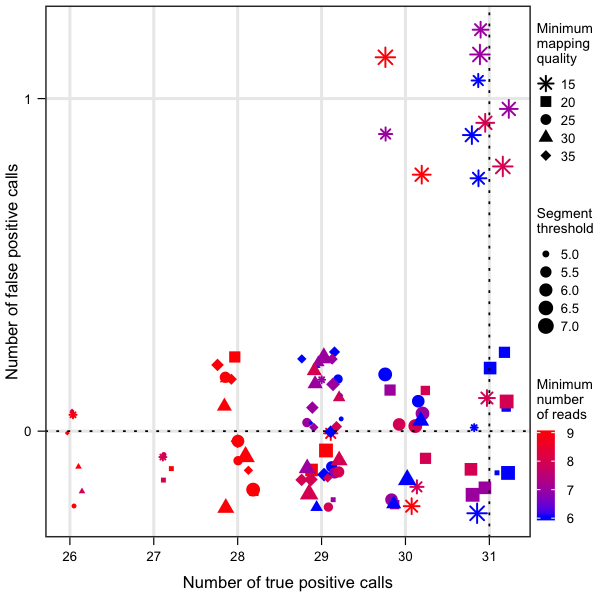
**

**Figure S15:** Number of true positive and false positive homozygous deletions called from the GBS data of the first and second libraries combined using different combinations of minimum number of reads per bin, segmentation threshold, and minimum mapping quality. Deletions are labeled as true positives if they overlap with a deletion found by the aCGH array and considered as false positives otherwise. Points were jittered along both the x and y directions to allow for points mapping to the same coordinates to be visualized. The intersection of the two dotted lines indicates the optimal results.

**
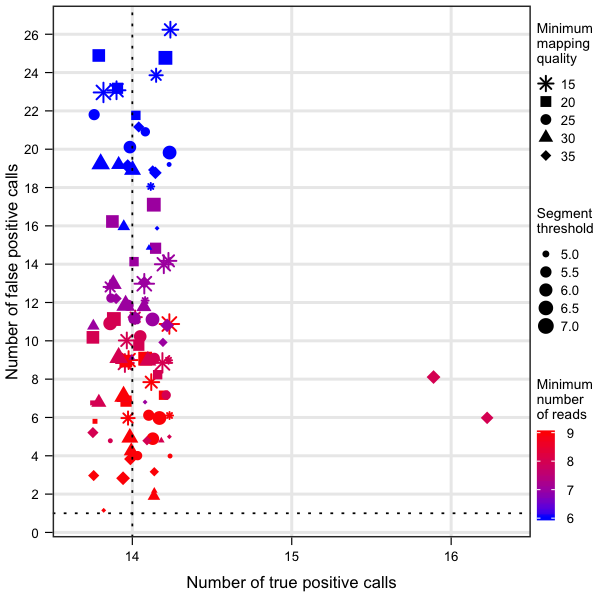
**

**Figure S16:** Number of true positive and false positive hemizygous deletions called from the GBS data of the first and second libraries combined using different combinations of minimum number of reads per bin, segmentation threshold, and minimum mapping quality. Deletions are labeled as true positives if they overlap with a deletion found by the aCGH array, and considered as false positives otherwise. Points were jittered along both the x and y directions to allow for points mapping to the same coordinates to be visualized. The intersection of the two dotted lines indicates the optimal results.

**
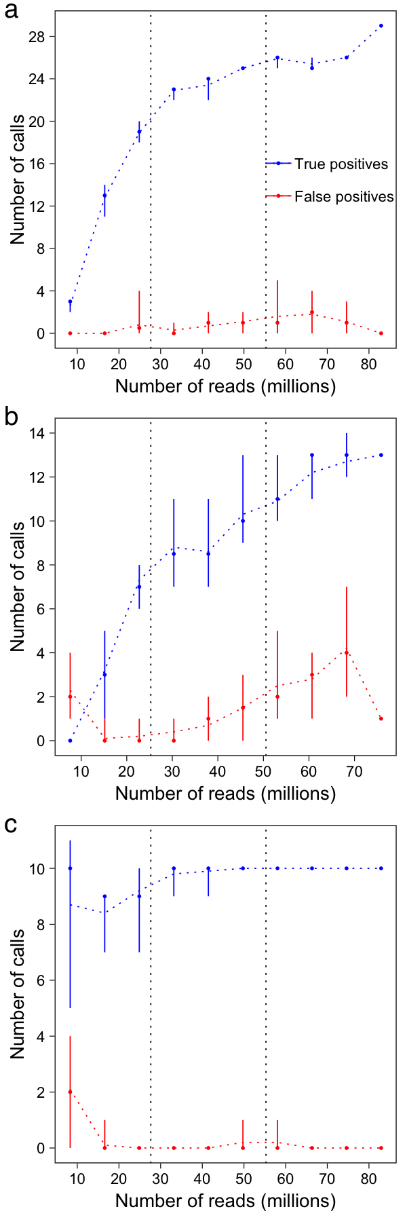
**

**Figure S17:** Number of true positive and false positive calls of different event types for various simulated sequencing depths using data from the three sequencing runs of the first GBS library. Events are labeled as true positives if they overlap with an event of similar type found by the aCGH array and considered as false positives otherwise. (a) Homozygous deletions. (b) Heterozygous deletions. (c) Duplications. Points represent the median, and solid vertical lines the minimum and maximum of 10 replications, while the dotted colored lines link the means of the replications. The largest number of reads in each graph corresponds to the full dataset, for which there are no replicates. The two vertical dotted lines represent the average number of mapped reads expected from one and two Ion Proton chips, respectively. Note that the reads originating from the 11 individuals that were removed from the analysis are not included.

**
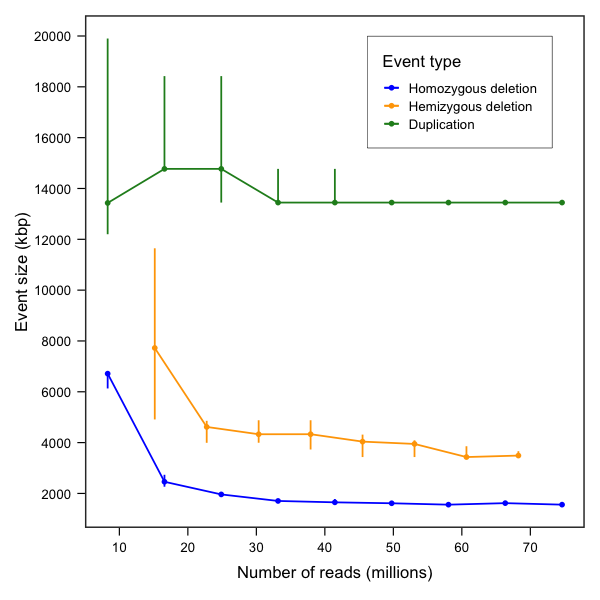
**

**Figure S18:** Mean size of different event types detected at different simulated sequencing depths. Points represent the median and solid vertical lines the minimum and maximum of the mean sizes of 10 replications. The highest number of reads in each graph corresponds to the full dataset, for which there are no replicates. No hemizygous deletions were called when sampling only 10% of the total number of reads, which explains the absence of data for this number of reads. Only true positives were included.

**
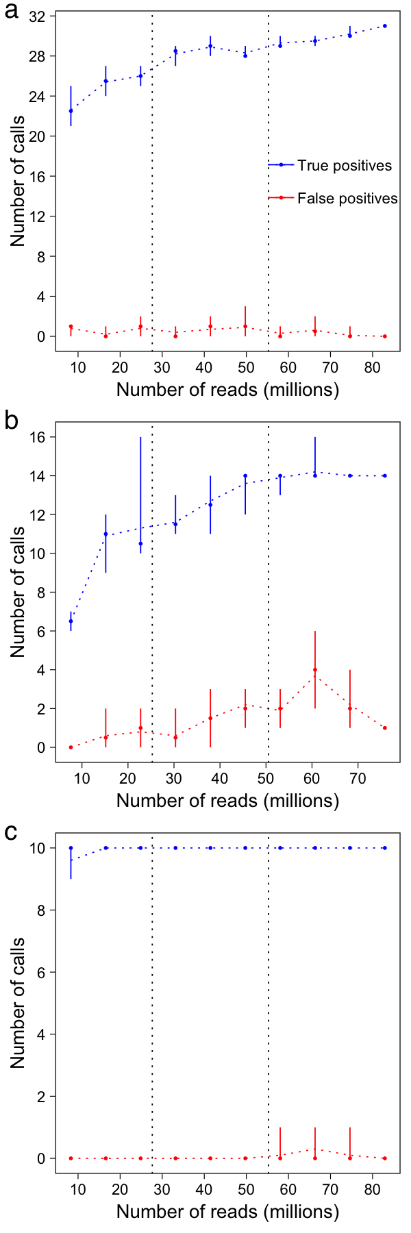
**

**Figure S19:** Number of true and false positive calls of different event types for various simulated sequencing depths combining data from the two GBS libraries. Events are labeled as true positives if they overlap with an event of similar type found by the aCGH array and considered as false positives otherwise. For each iteration, the complete dataset from the second library was added to reads sampled at different proportions from the three sequencing runs of the first library. The number of reads on the x-axis corresponds to the number of reads sampled from the first library. An additional 38.9 million reads came from the second library in subfigures a and c, and 35.9 million reads in subfigure b. (a) Homozygous deletions. (b) Heterozygous deletions. (c) Duplications. Points represent the median, and solid vertical lines the minimum and maximum of 10 replications, while the dotted colored lines link the means of the replications. The largest number of reads in each graph corresponds to the full dataset, for which there are no replicates. The two vertical dotted lines represent the average number of mapped reads expected from one and two Ion Proton chips of the first library, respectively. Note that the reads originating from the 11 individuals that were removed from the analysis are not included.


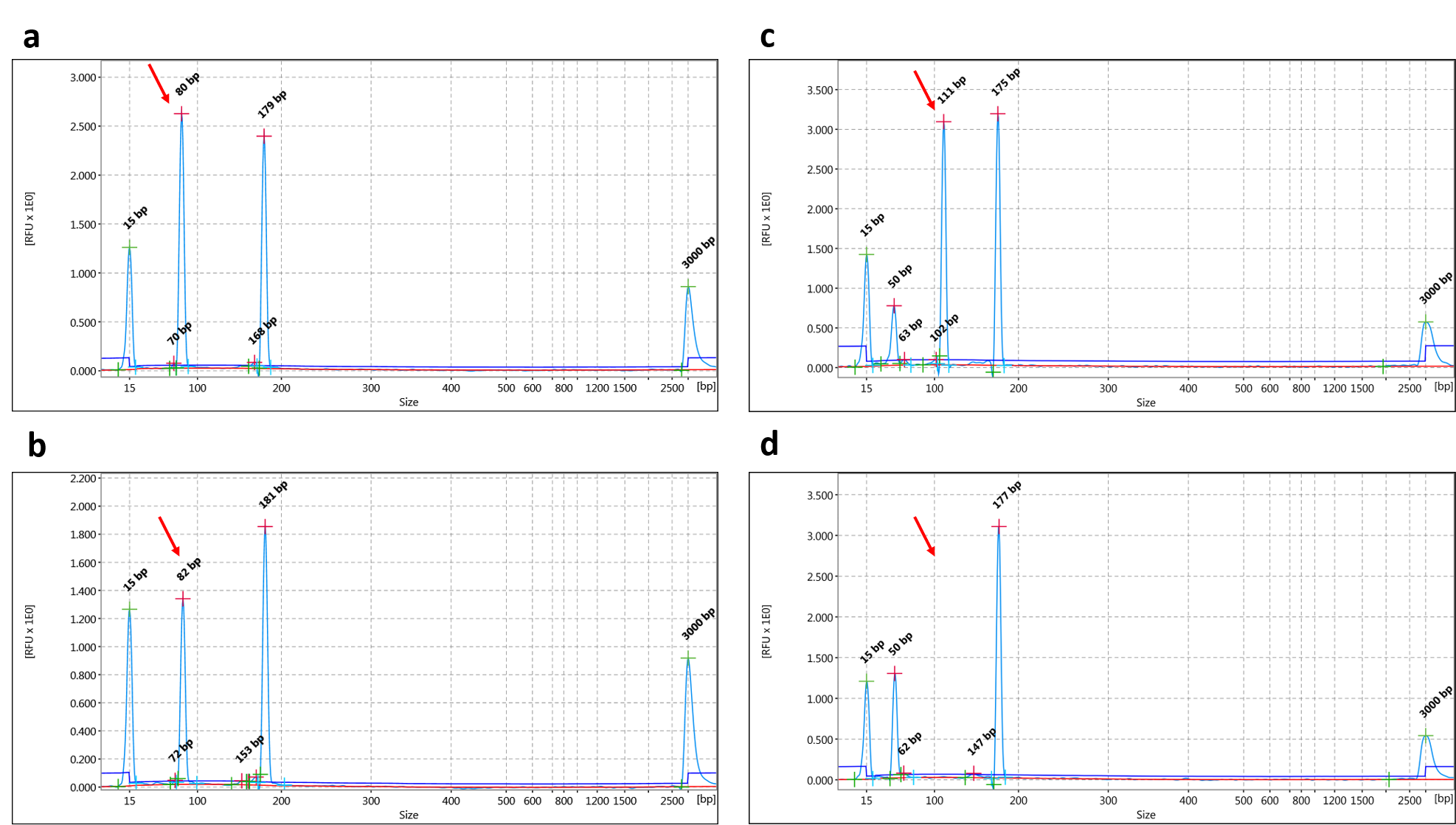


**Figure S20:** Electropherograms showing relative fluorescence units (RFU) as a function of fragment size (in bp) produced by the QIAxcel system for the validation of the putative homozygous deletion in mutant line FNMN0012 at position Chr14:14,436,000-14,508,000 by PCR amplification using diagnostic and control primer pairs on control wild-type DNA (a, c) and mutant DNA (b, d). (a-b) Amplification with control primer pair AW (expected amplicon size of 173 bp) and diagnostic primer pair BI (expected amplicon size of 75 bp). (c-d) Amplification with control primer pair AW (expected amplicon size of 173 bp) and diagnostic primer pair BZ (expected amplicon size of 105 bp). Only primer pair BZ supported the existence of a deletion at this locus in FNMN0012. The red arrows indicate the location (or expected location) of the diagnostic amplicons. The bands observed at 15 bp and 3,000 bp are the size markers.


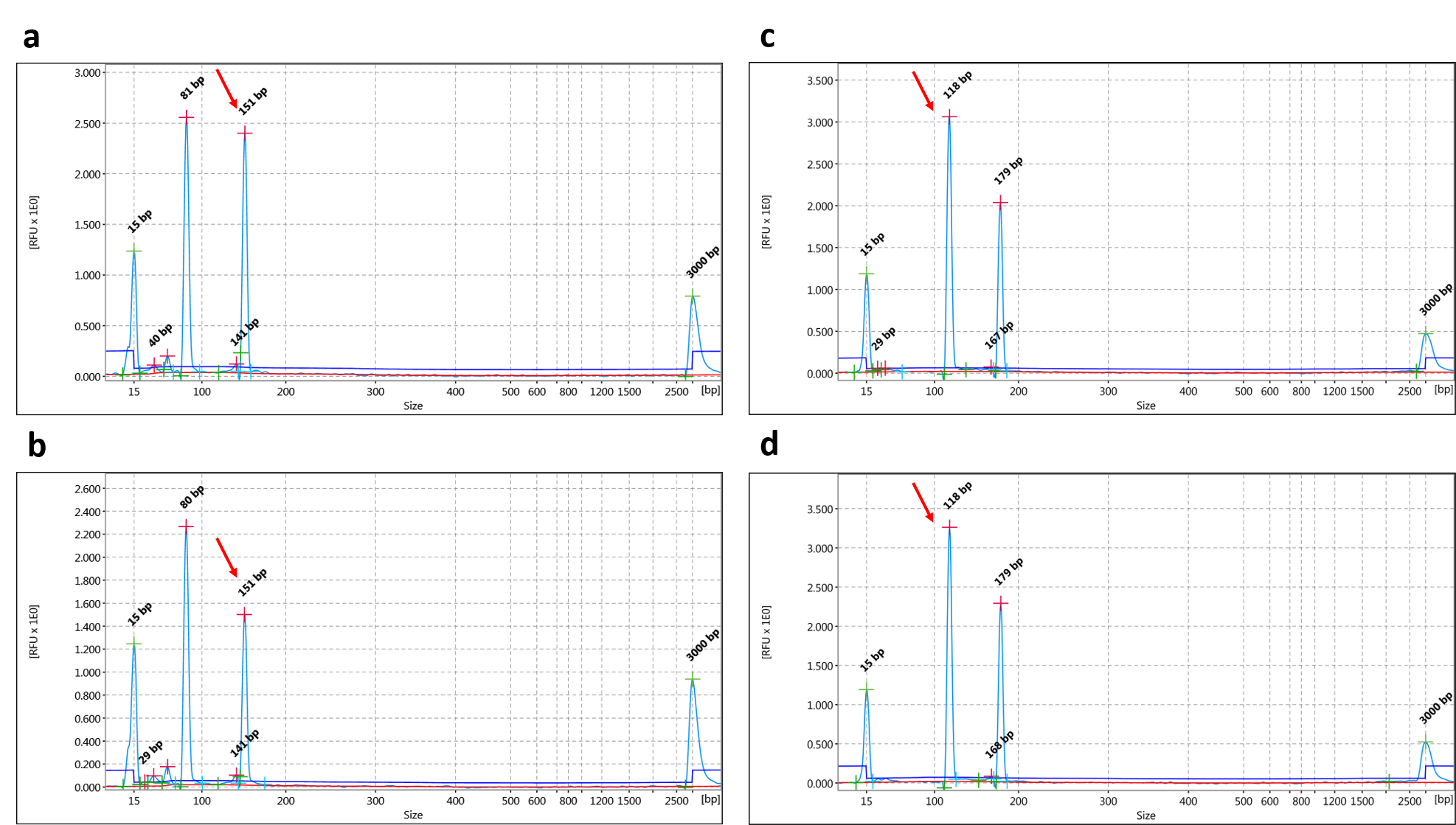


**Figure S21:** Electropherograms showing relative fluorescence units (RFU) as a function of fragment size (in bp) produced by the QIAxcel system for the validation of the putative homozygous deletion in mutant line FNMN0017 at position Chr08:9,450,000-9,458,000 by PCR amplification using diagnostic and control primer pairs on control wild-type DNA (a, c) and mutant DNA (b, d). (a-b) Amplification with control primer pair AM (expected amplicon size of 75 bp) and diagnostic primer pair AR (expected amplicon size of 146 bp). (c-d) Amplification with control primer pair AW (expected amplicon size of 173 bp) and diagnostic primer pair AS (expected amplicon size of 112 bp). None of the primer pairs supported the existence of a deletion at this locus in FNMN0017. The red arrows indicate the location (or expected location) of the diagnostic amplicons. The bands observed at 15 bp and 3,000 bp are the size markers.


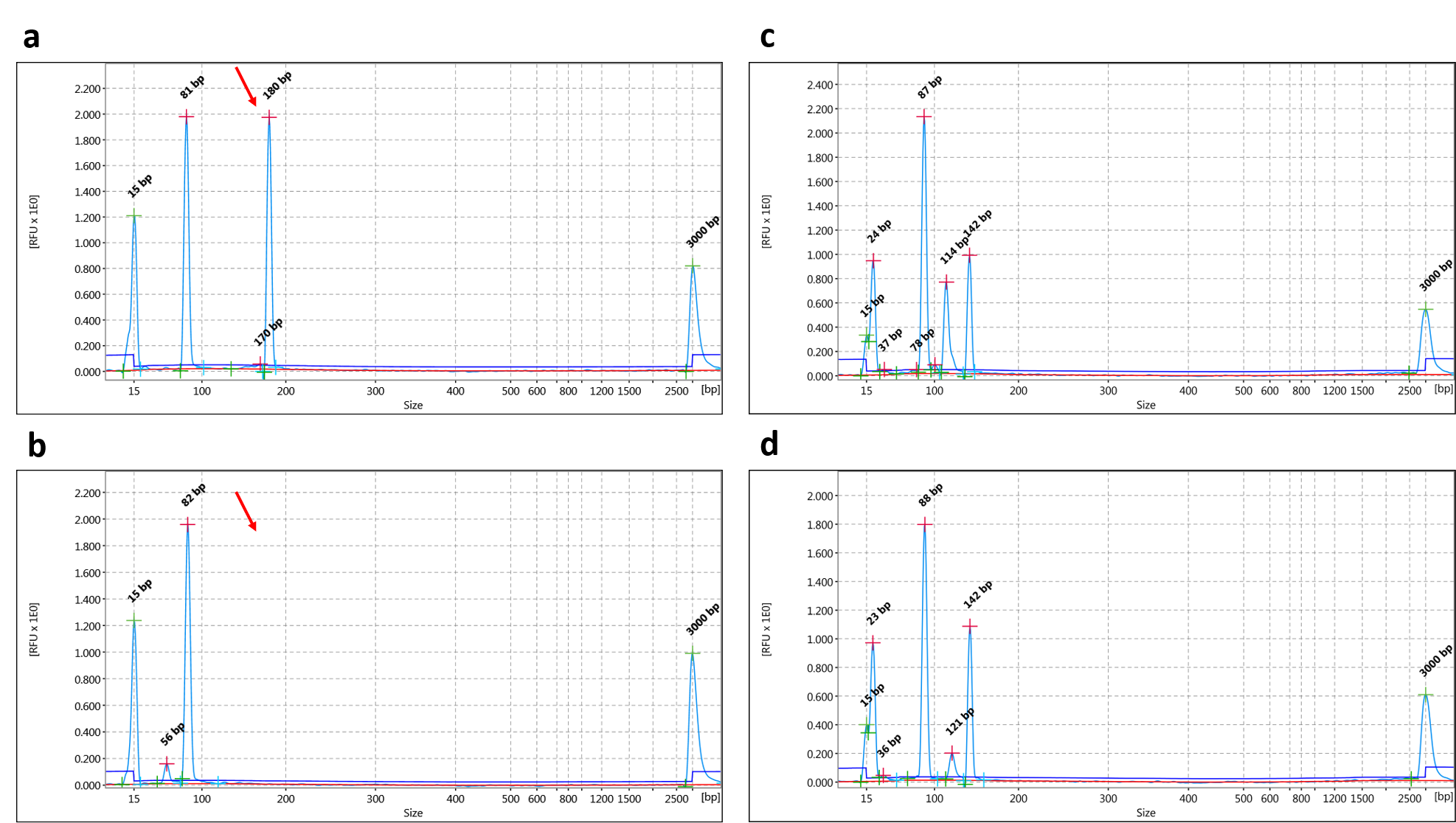


**Figure S22:** Electropherograms showing relative fluorescence units (RFU) as a function of fragment size (in bp) produced by the QIAxcel system for the validation of the putative homozygous deletion in mutant line FNMN0037 at position Chr10:6,034,000-6,069,000 by PCR amplification using diagnostic and control primer pairs on control wild-type DNA (a, c) and mutant DNA (b, d). (a-b) Amplification with control primer pair AM (expected amplicon size of 75 bp) and diagnostic primer pair AW (expected amplicon size of 173 bp). (c-d) Amplification with control primer pair AM (expected amplicon size of 75 bp) and diagnostic primer pair AX (expected amplicon size of 100 bp). Primer pair AW supported the existence of a deletion at this locus in FNMN0037, but no conclusion could be made from primer pair AX as it was unclear whether the intended target was generated. The red arrows indicate the location (or expected location) of the diagnostic amplicons. The bands observed at 15 bp and 3,000 bp are the size markers.


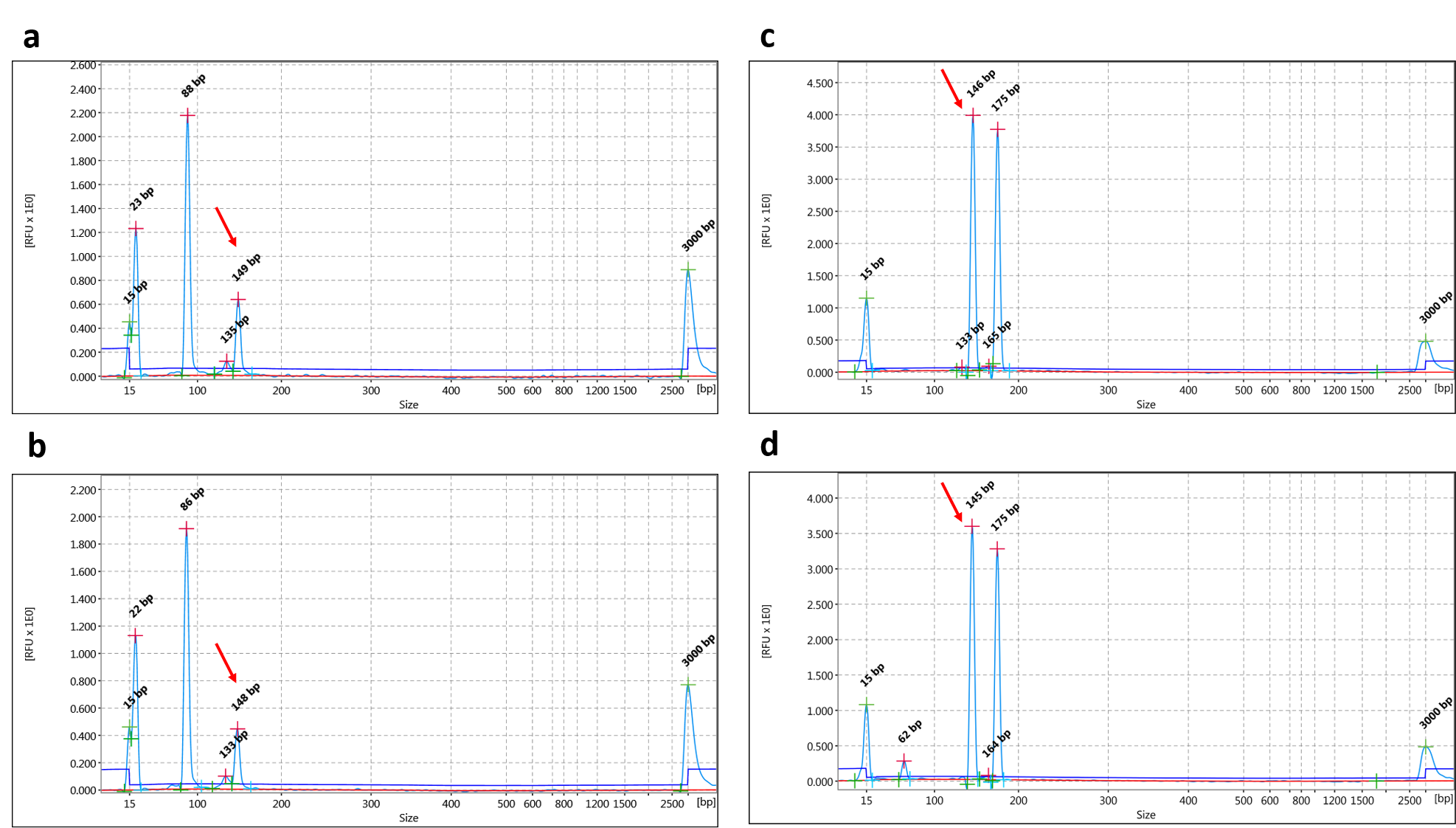


**Figure S23:** Electropherograms showing relative fluorescence units (RFU) as a function of fragment size (in bp) produced by the QIAxcel system for the validation of the putative homozygous deletion in mutant line FNMN0038 at position Chr01:47,907,000-47,936,000 by PCR amplification using diagnostic and control primer pairs on control wild-type DNA (a, c) and mutant DNA (b, d). (a-b) Amplification with control primer pair AM (expected amplicon size of 75 bp) and diagnostic primer pair AH (expected amplicon size of 133 bp). (c-d) Amplification with control primer pair AW (expected amplicon size of 173 bp) and diagnostic primer pair CB (expected amplicon size of 142 bp). None of the primer pairs supported the existence of a deletion at this locus in FNMN0038. The red arrows indicate the location (or expected location) of the diagnostic amplicons. The bands observed at 15 bp and 3,000 bp are the size markers.


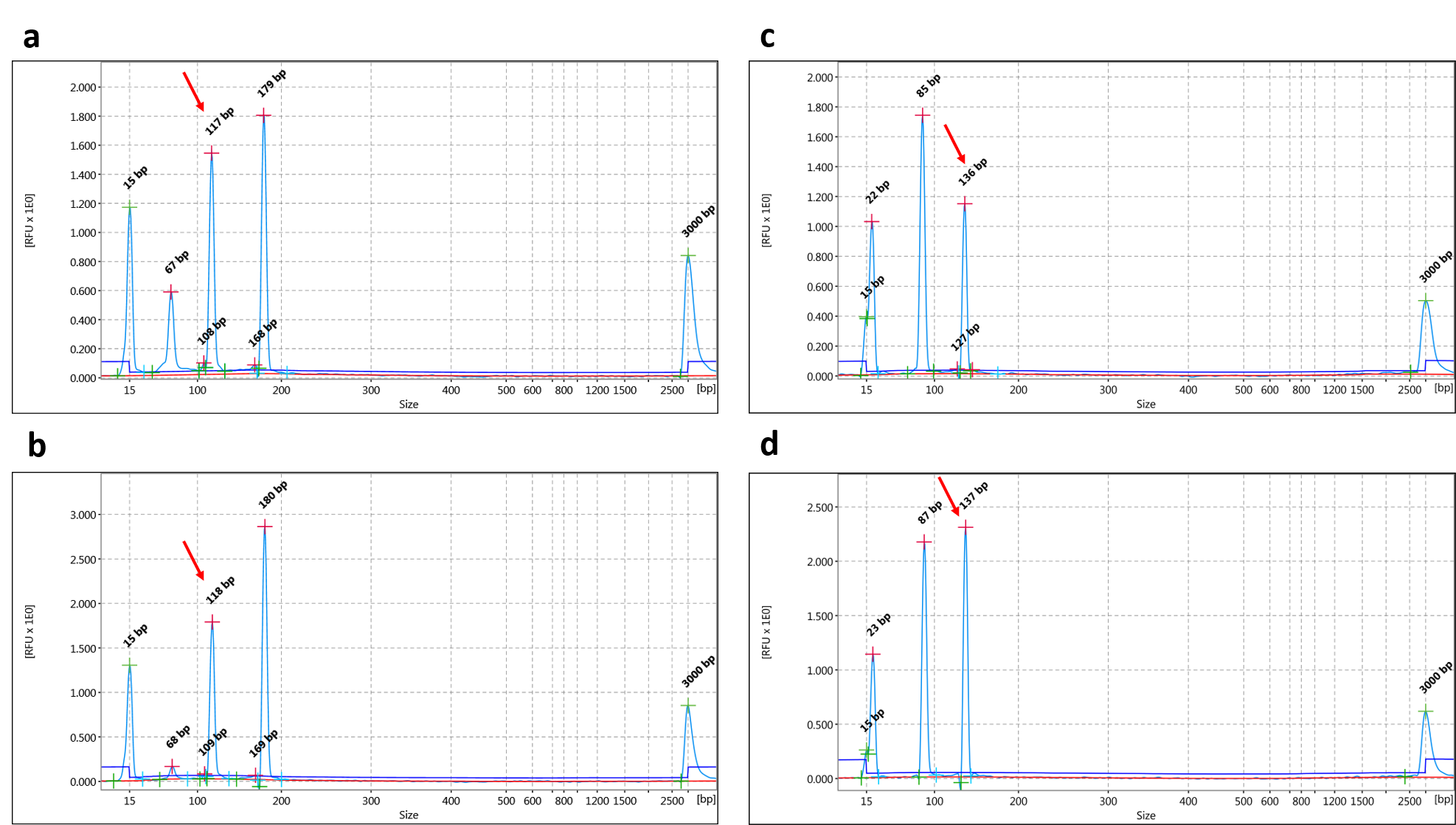


**Figure S24:** Electropherograms showing relative fluorescence units (RFU) as a function of fragment size (in bp) produced by the QIAxcel system for the validation of the putative homozygous deletion in mutant line FNMN0038 at position Chr09:39,115,000-39,202,000 by PCR amplification using diagnostic and control primer pairs on control wild-type DNA (a, c) and mutant DNA (b, d). (a-b) Amplification with control primer pair AW (expected amplicon size of 173 bp) and diagnostic primer pair AU (expected amplicon size of 110 bp). (c-d) Amplification with control primer pair AM (expected amplicon size of 75 bp) and diagnostic primer pair AV (expected amplicon size of 122 bp). None of the primer pairs supported the existence of a deletion at this locus in FNMN0038. The red arrows indicate the location (or expected location) of the diagnostic amplicons. The bands observed at 15 bp and 3,000 bp are the size markers.


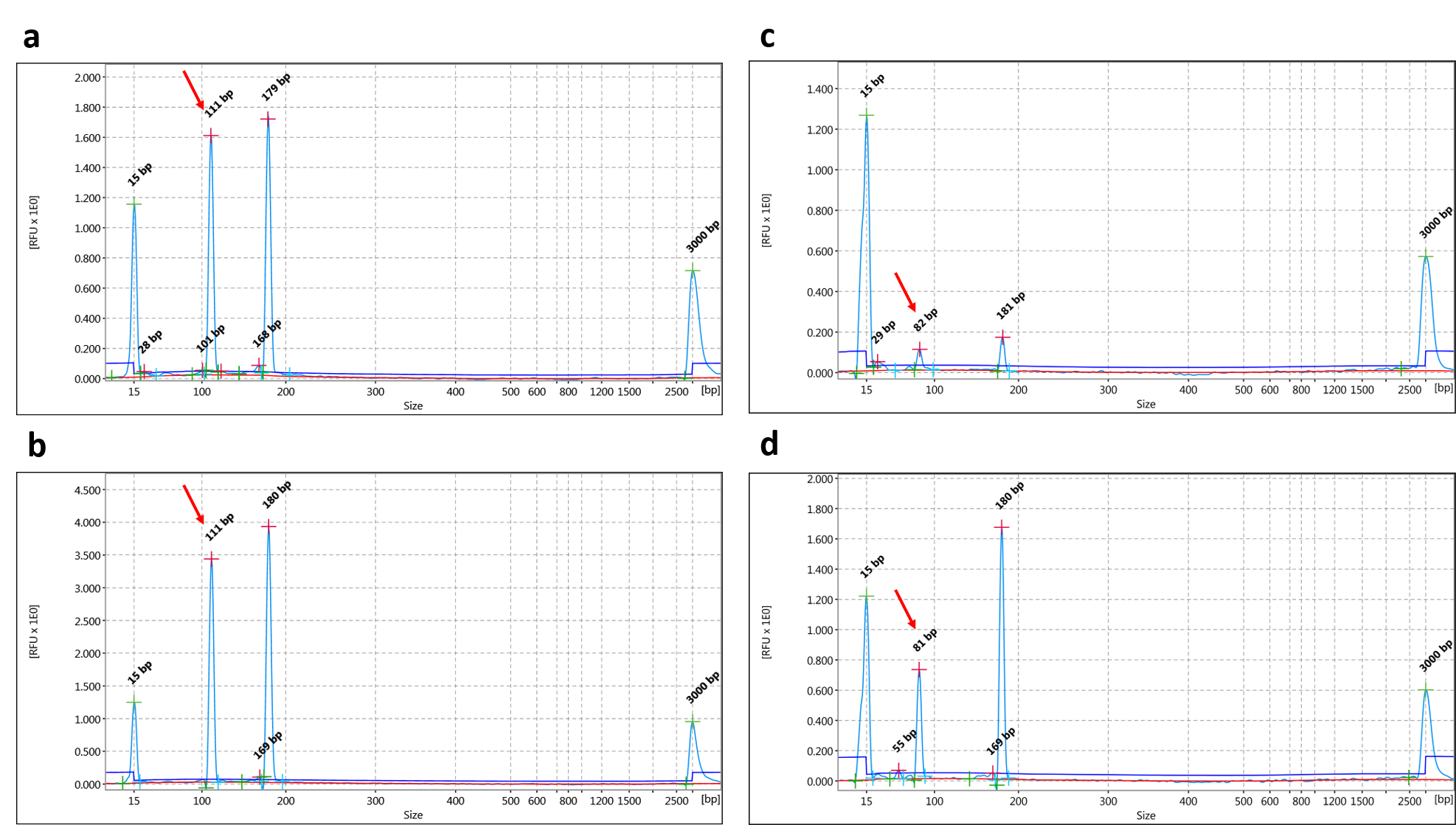


**Figure S25:** Electropherograms showing relative fluorescence units (RFU) as a function of fragment size (in bp) produced by the QIAxcel system for the validation of the putative homozygous deletion in mutant line FNMN0038 at position Chr13:23,112,000-23,126,000 by PCR amplification using diagnostic and control primer pairs on control wild-type DNA (a, c) and mutant DNA (b, d). (a-b) Amplification with control primer pair AW (expected amplicon size of 173 bp) and diagnostic primer pair BG (expected amplicon size of 101 bp). (c-d) Amplification with control primer pair AW (expected amplicon size of 173 bp) and diagnostic primer pair BH (expected amplicon size of 75 bp). None of the primer pairs supported the existence of a deletion at this locus in FNMN0038. The red arrows indicate the location (or expected location) of the diagnostic amplicons. The bands observed at 15 bp and 3,000 bp are the size markers.


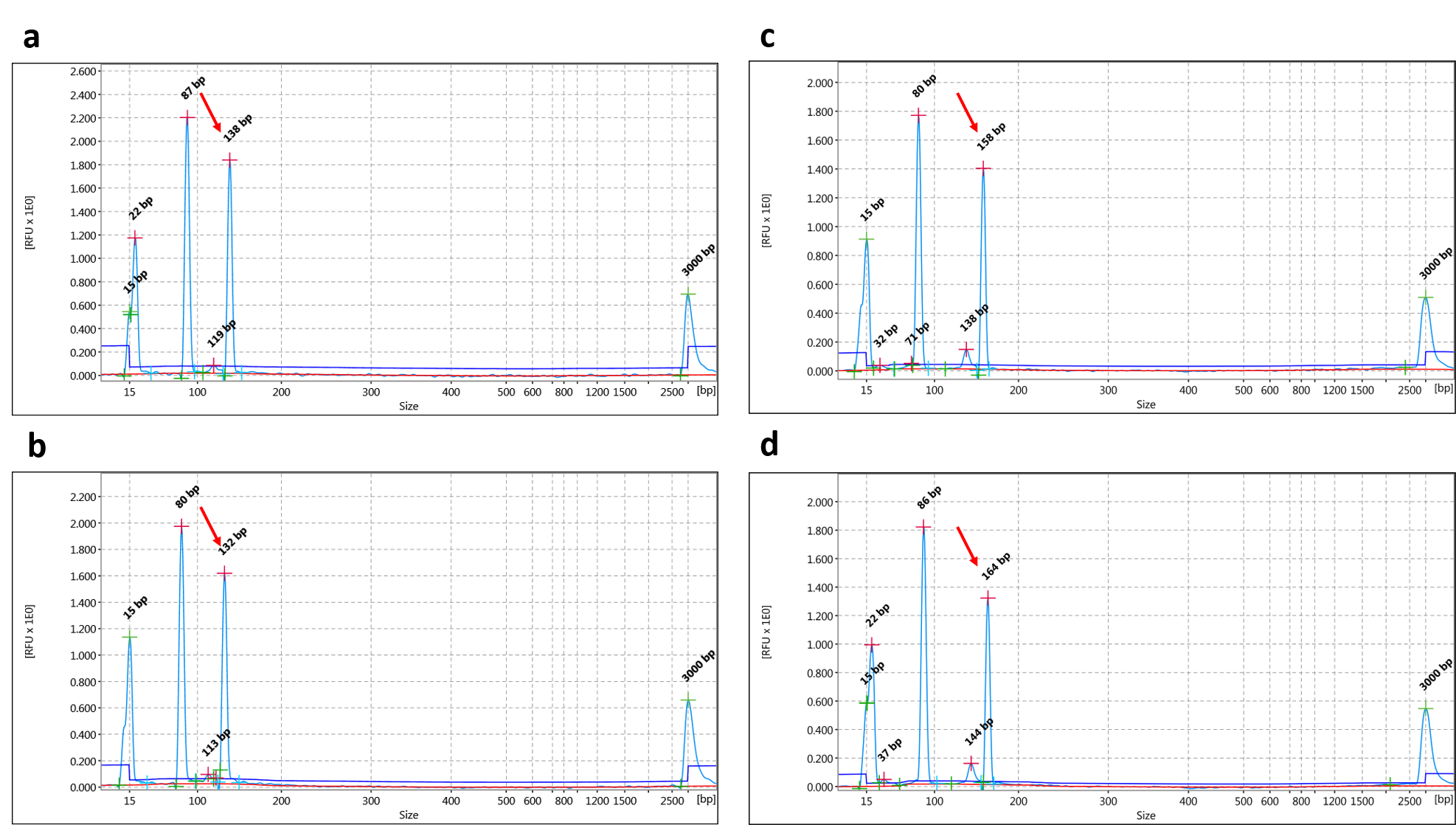


**Figure S26:** Electropherograms showing relative fluorescence units (RFU) as a function of fragment size (in bp) produced by the QIAxcel system for the validation of the putative homozygous deletion in mutant line FNMN0038 at position Chr16:33,120,000-33,137,000 by PCR amplification using diagnostic and control primer pairs on control wild-type DNA (a, c) and mutant DNA (b, d). (a-b) Amplification with control primer pair AM (expected amplicon size of 75 bp) and diagnostic primer pair BM (expected amplicon size of 126 bp). (c-d) Amplification with control primer pair AM (expected amplicon size of 75 bp) and diagnostic primer pair BL (expected amplicon size of 150 bp). None of the primer pairs supported the existence of a deletion at this locus in FNMN0038. The red arrows indicate the location (or expected location) of the diagnostic amplicons. The bands observed at 15 bp and 3,000 bp are the size markers.


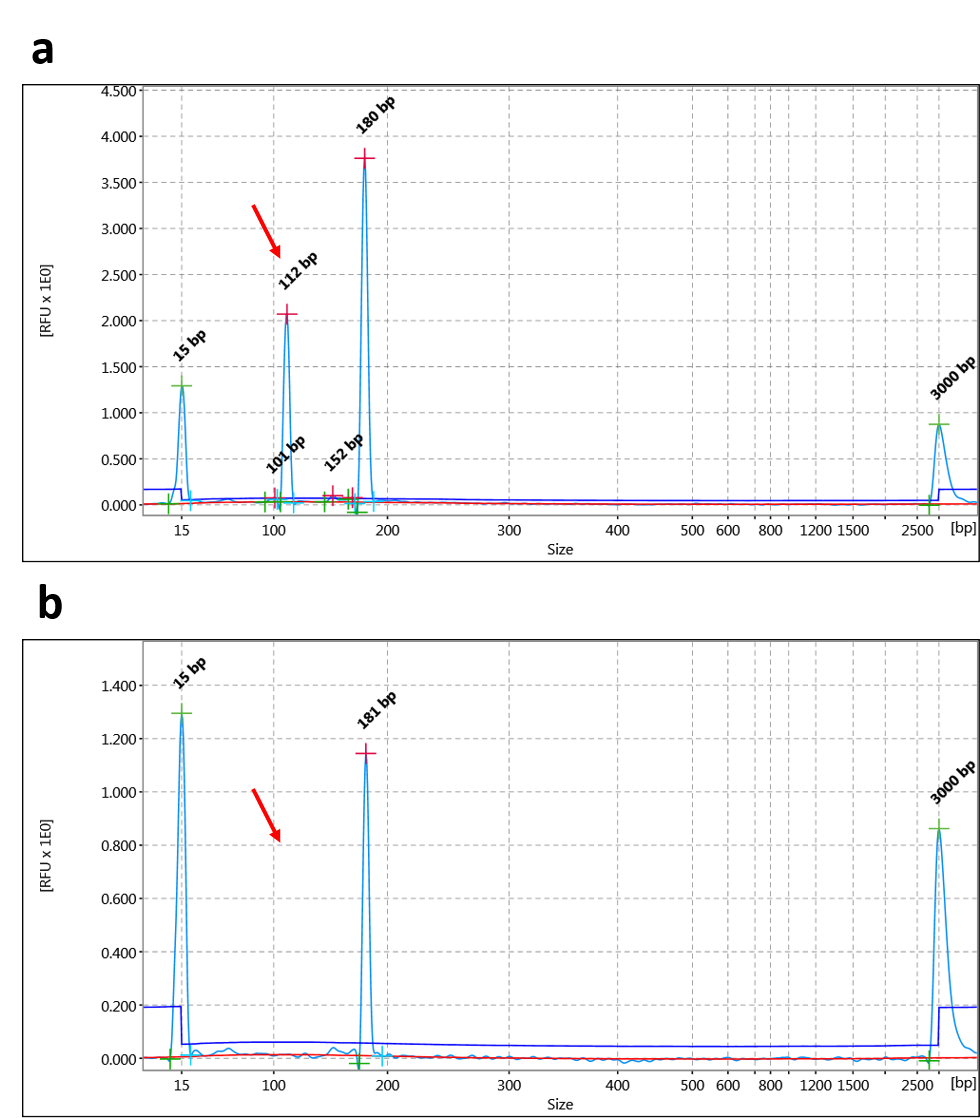


**Figure S27:** Electropherograms showing relative fluorescence units (RFU) as a function of fragment size (in bp) produced by the QIAxcel system for the validation of the putative homozygous deletion in mutant line FNMN0049 at position Chr14:24,802,000-24,913,000 by PCR amplification using diagnostic and control primer pairs on control wild-type DNA (a) and mutant DNA (b). (a-b) Amplification with control primer pair AW (expected amplicon size of 173 bp) and diagnostic primer pair BJ (expected amplicon size of 101 bp). Primer pair BJ supported the existence of a deletion at this locus in FNMN0049. The red arrows indicate the location (or expected location) of the diagnostic amplicons. The bands observed at 15 bp and 3,000 bp are the size markers.


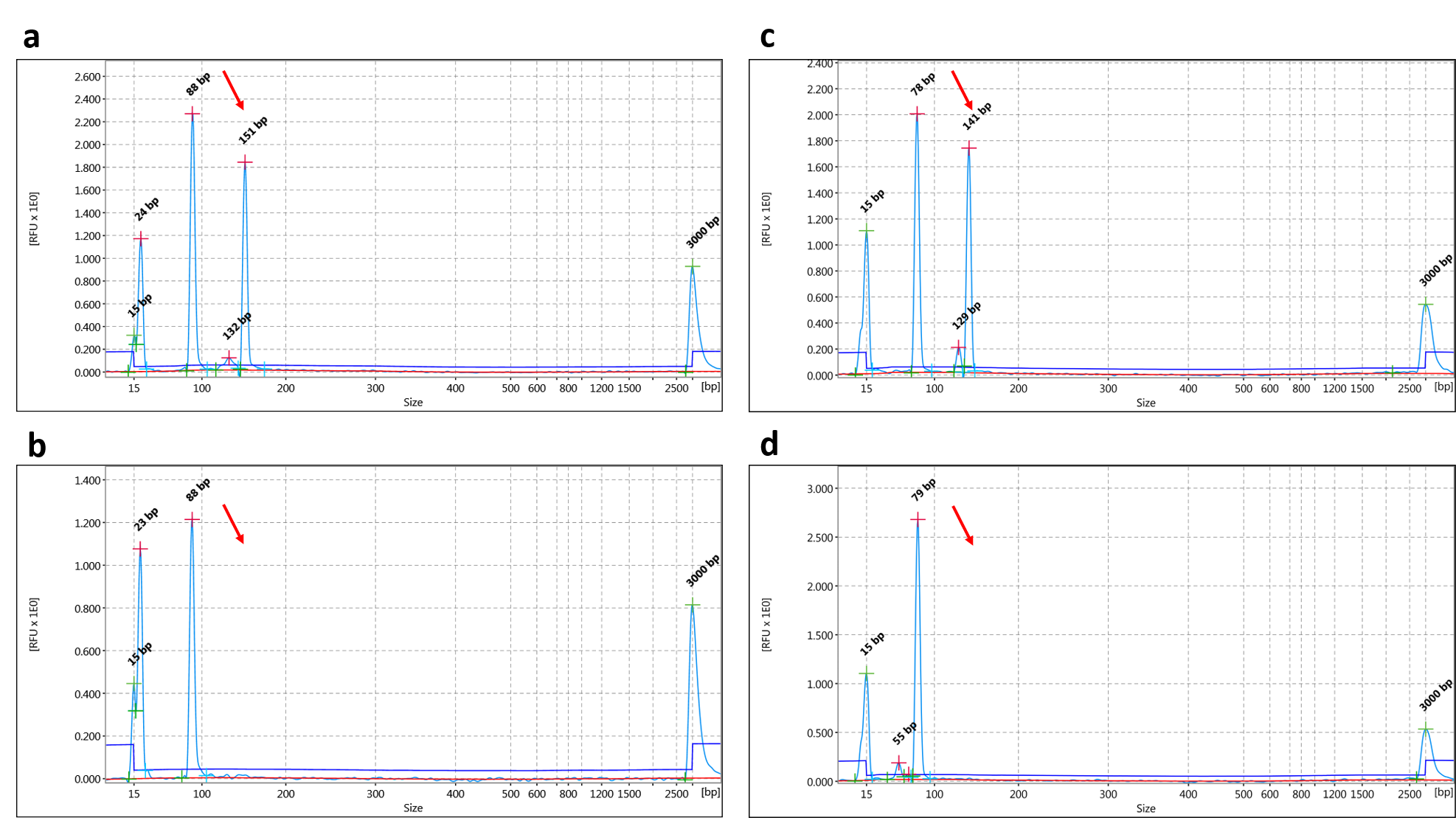


**Figure S28:** Electropherograms showing relative fluorescence units (RFU) as a function of fragment size (in bp) produced by the QIAxcel system for the validation of the putative homozygous deletion in mutant line FNMN0051 at position Chr01:46,717,000-46,729,000 by PCR amplification using diagnostic and control primer pairs on control wild-type DNA (a, c) and mutant DNA (b, d). (a-b) Amplification with control primer pair AM (expected amplicon size of 75 bp) and diagnostic primer pair AC (expected amplicon size of 137 bp). (c-d) Amplification with control primer pair AM (expected amplicon size of 75 bp) and diagnostic primer pair AD (expected amplicon size of 136 bp). Both diagnostic primer pairs supported the existence of a deletion at this locus in FNMN0051. The red arrows indicate the location (or expected location) of the diagnostic amplicons. The bands observed at 15 bp and 3,000 bp are the size markers.


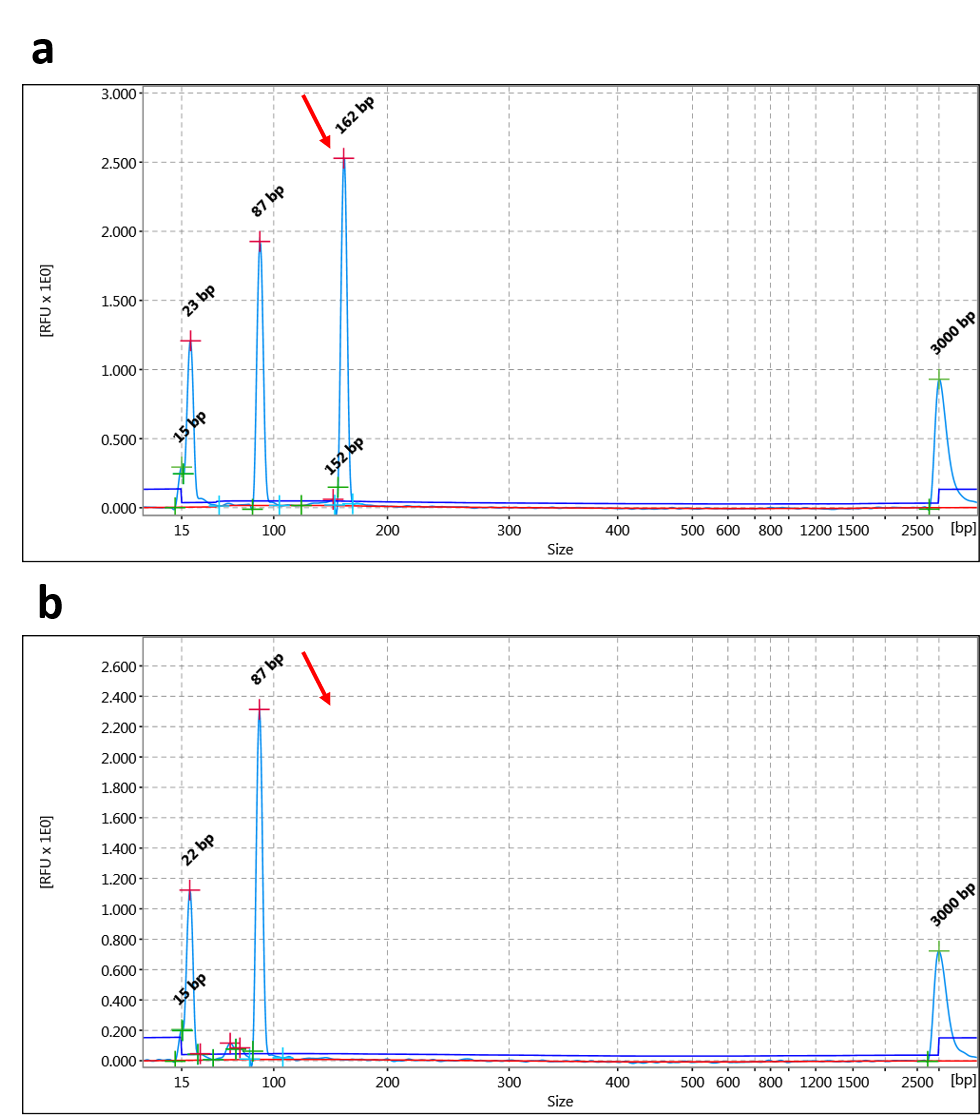


**Figure S29:** Electropherograms showing relative fluorescence units (RFU) as a function of fragment size (in bp) produced by the QIAxcel system for the validation of the putative homozygous deletion in mutant line FNMN0055 at position Chr20:40,774,000-40,782,000 by PCR amplification using diagnostic and control primer pairs on control wild-type DNA (a) and mutant DNA (b). (a-b) Amplification with control primer pair AM (expected amplicon size of 75 bp) and diagnostic primer pair BU (expected amplicon size of 149 bp). Primer pair BU supported the existence of a deletion at this locus in FNMN0055. The red arrows indicate the location (or expected location) of the diagnostic amplicons. The bands observed at 15 bp and 3,000 bp are the size markers.


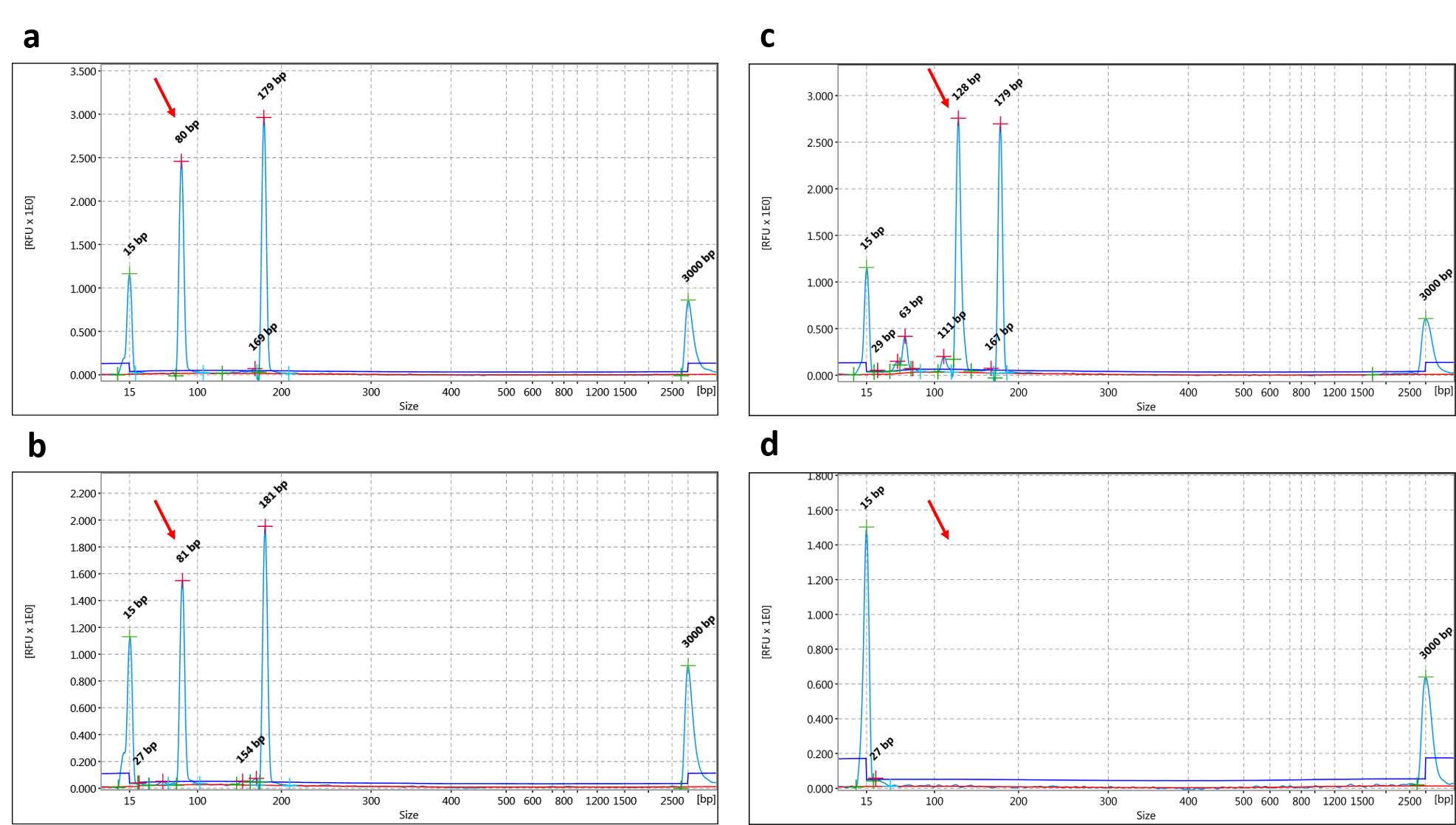


**Figure S30:** Electropherograms showing relative fluorescence units (RFU) as a function of fragment size (in bp) produced by the QIAxcel system for the validation of the putative homozygous deletion in mutant line FNMN0057 at position Chr05:29,564,000-29,766,000 by PCR amplification using diagnostic and control primer pairs on control wild-type DNA (a, c) and mutant DNA (b, d). (a-b) Amplification with control primer pair AW (expected amplicon size of 173 bp) and diagnostic primer pair AM (expected amplicon size of 75 bp). (c-d) Amplification with control primer pair AW (expected amplicon size of 173 bp) and diagnostic primer pair AN (expected amplicon size of 119 bp). Primer pair AM did not support the existence of a deletion at this locus in FNMN0057, whereas we could not conclude from primer pair AN as neither this primer pair nor the control pair amplified in the mutant. The red arrows indicate the location (or expected location) of the diagnostic amplicons. The bands observed at 15 bp and 3,000 bp are the size markers.


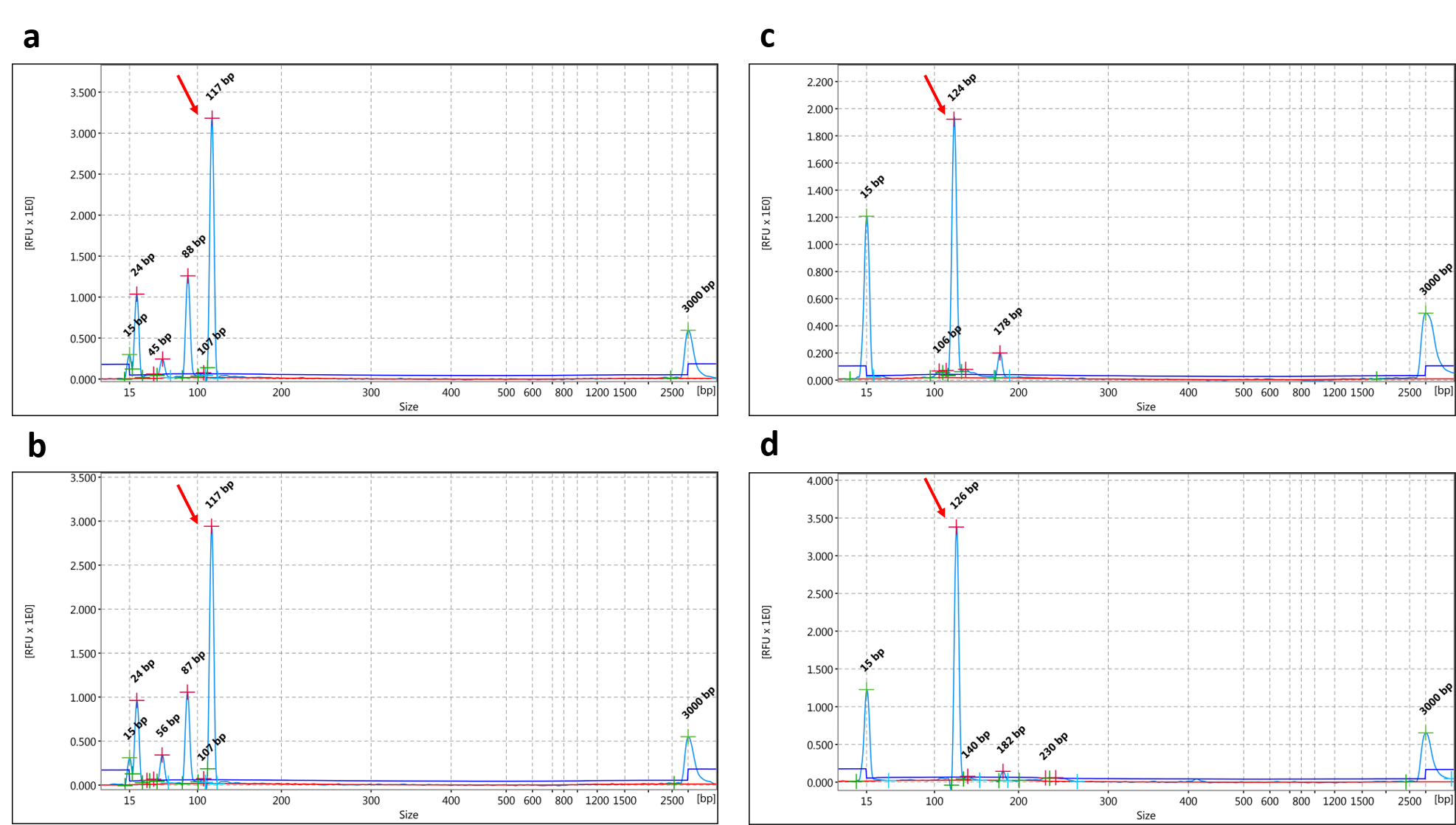


**Figure S31:** Electropherograms showing relative fluorescence units (RFU) as a function of fragment size (in bp) produced by the QIAxcel system for the validation of the putative homozygous deletion in mutant line FNMN0064 at position Chr06:47,068,000-47,086,000 by PCR amplification using diagnostic and control primer pairs on control wild-type DNA (a, c) and mutant DNA (b, d). (a-b) Amplification with control primer pair AM (expected amplicon size of 75 bp) and diagnostic primer pair AO (expected amplicon size of 101 bp). (c-d) Amplification with control primer pair AW (expected amplicon size of 173 bp) and diagnostic primer pair CD (expected amplicon size of 117 bp). None of the primer pairs supported the existence of a deletion at this locus in FNMN0064. The red arrows indicate the location (or expected location) of the diagnostic amplicons. The bands observed at 15 bp and 3,000 bp are the size markers.


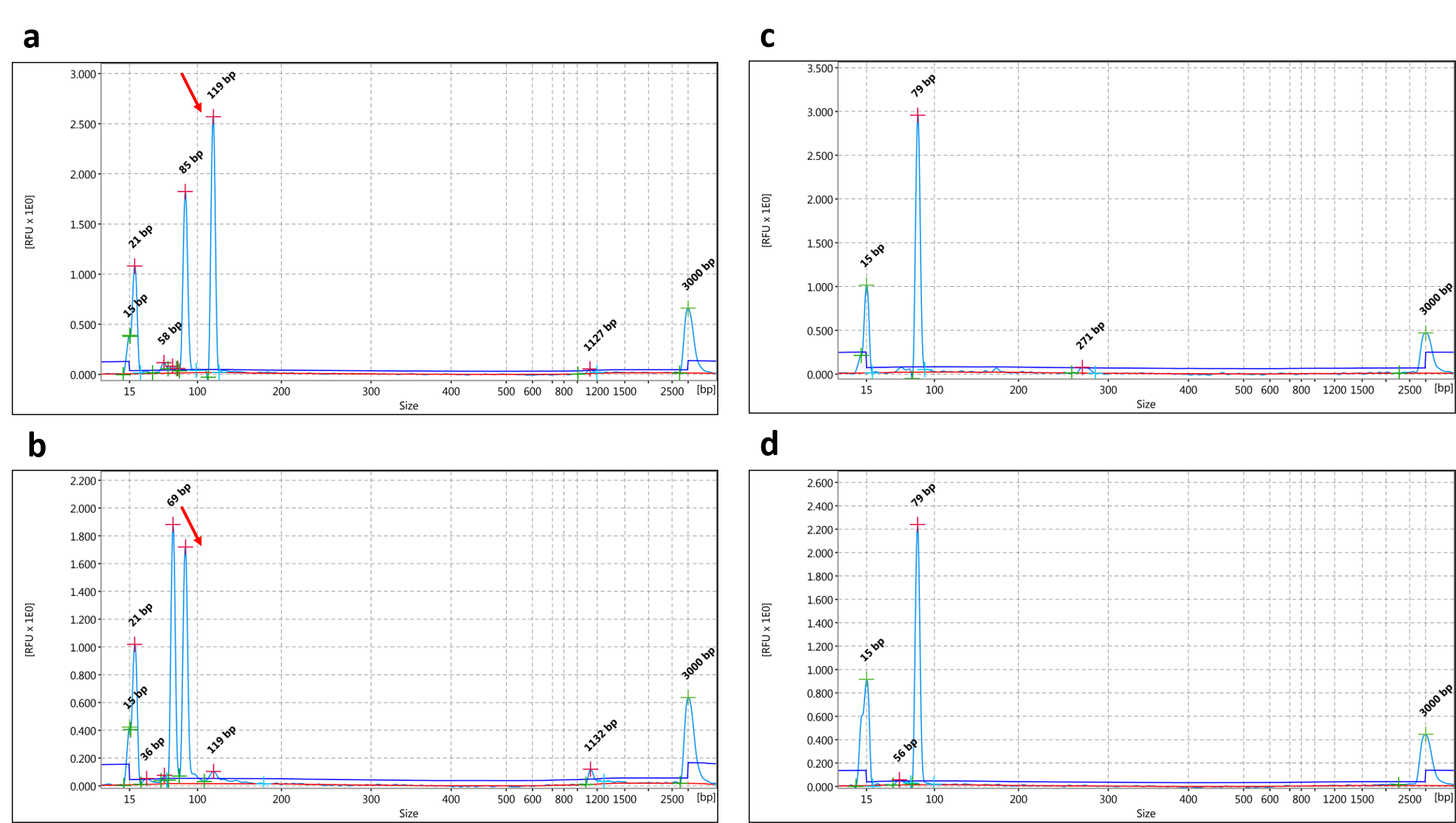


**Figure S32:** Electropherograms showing relative fluorescence units (RFU) as a function of fragment size (in bp) produced by the QIAxcel system for the validation of the putative homozygous deletion in mutant line FNMN0064 at position Chr20:35,731,000-35,750,000 by PCR amplification using diagnostic and control primer pairs on control wild-type DNA (a, c) and mutant DNA (b, d). (a-b) Amplification with control primer pair AM (expected amplicon size of 75 bp) and diagnostic primer pair BT (expected amplicon size of 107 bp). (c-d) Amplification with control primer pair AM (expected amplicon size of 75 bp) and diagnostic primer pair BS (expected amplicon size of 146 bp). Primer pair BT supported the existence of a deletion at this locus in FNMN0064, although a band not observed in the control was observed in the mutant. We could not conclude from primer pair BS as it amplified neither in the control nor in the mutant. The red arrows indicate the location (or expected location) of the diagnostic amplicons. The bands observed at 15 bp and 3,000 bp are the size markers.


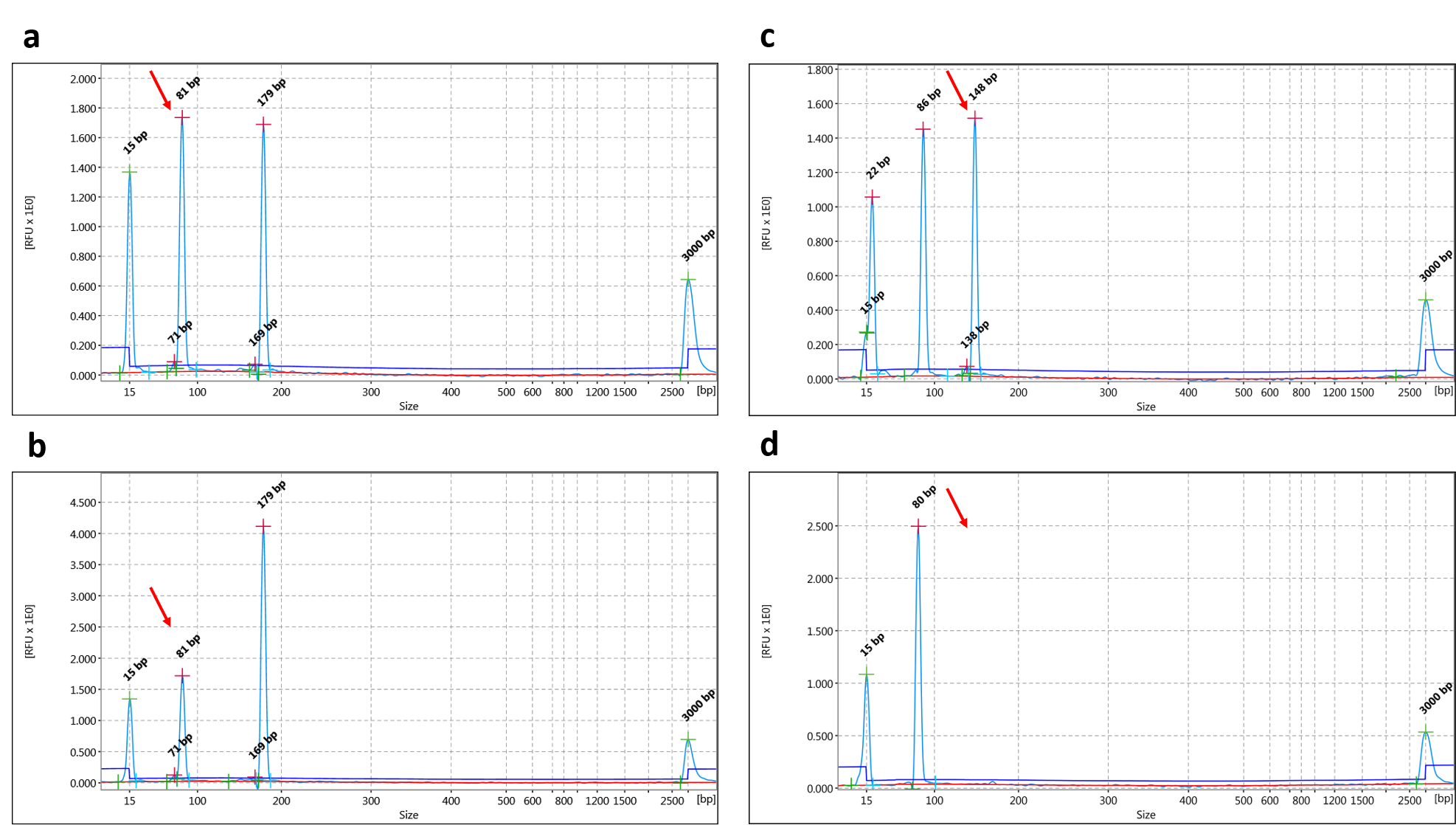


**Figure S33:** Electropherograms showing relative fluorescence units (RFU) as a function of fragment size (in bp) produced by the QIAxcel system for the validation of the putative homozygous deletion in mutant line FNMN0086 at position Chr17:31,528,000-31,674,000 by PCR amplification using diagnostic and control primer pairs on control wild-type DNA (a, c) and mutant DNA (b, d). (a-b) Amplification with control primer pair AW (expected amplicon size of 173 bp) and diagnostic primer pair BO (expected amplicon size of 75 bp). (c-d) Amplification with control primer pair AM (expected amplicon size of 75 bp) and diagnostic primer pair BN (expected amplicon size of 135 bp). Only primer pair BN supported the existence of a deletion at this locus in FNMN0086. The red arrows indicate the location (or expected location) of the diagnostic amplicons. The bands observed at 15 bp and 3,000 bp are the size markers.


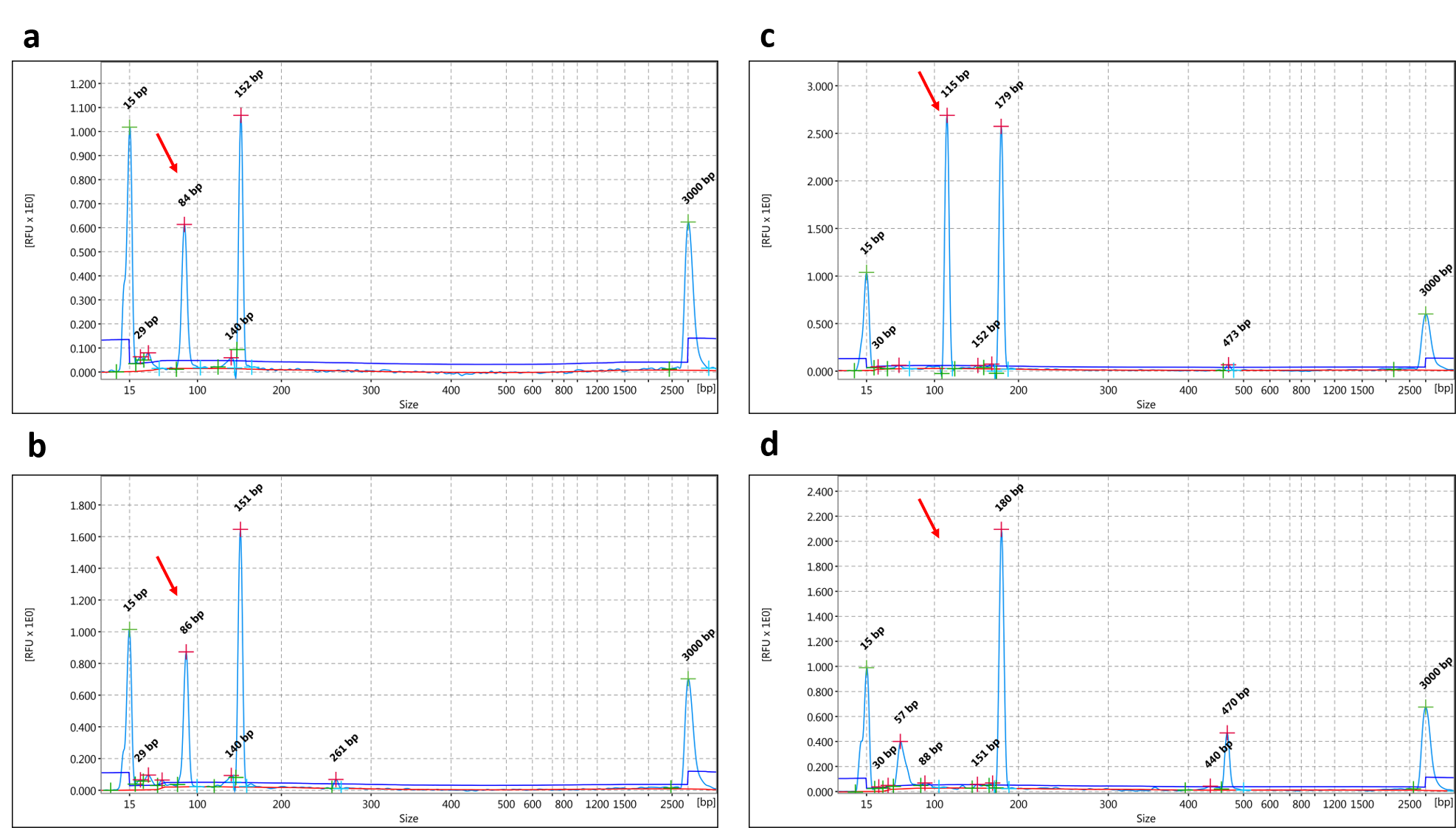


**Figure S34:** Electropherograms showing relative fluorescence units (RFU) as a function of fragment size (in bp) produced by the QIAxcel system for the validation of the putative homozygous deletion in control line M92-220-Long R6-1 at position Chr02:21,966,000-22,879,000 by PCR amplification using diagnostic and control primer pairs on control wild-type DNA (a, c) and M92-220-Long R6-1 DNA (b, d). (a-b) Amplification with control primer pair AR (expected amplicon size of 146 bp) and diagnostic primer pair AI (expected amplicon size of 77 bp). (c-d) Amplification with control primer pair AW (expected amplicon size of 173 bp) and diagnostic primer pair AJ (expected amplicon size of 106 bp). Only primer pair AJ supported the existence of a deletion at this locus in M92-220-Long R6-1. The red arrows indicate the location (or expected location) of the diagnostic amplicons. The bands observed at 15 bp and 3,000 bp are the size markers.


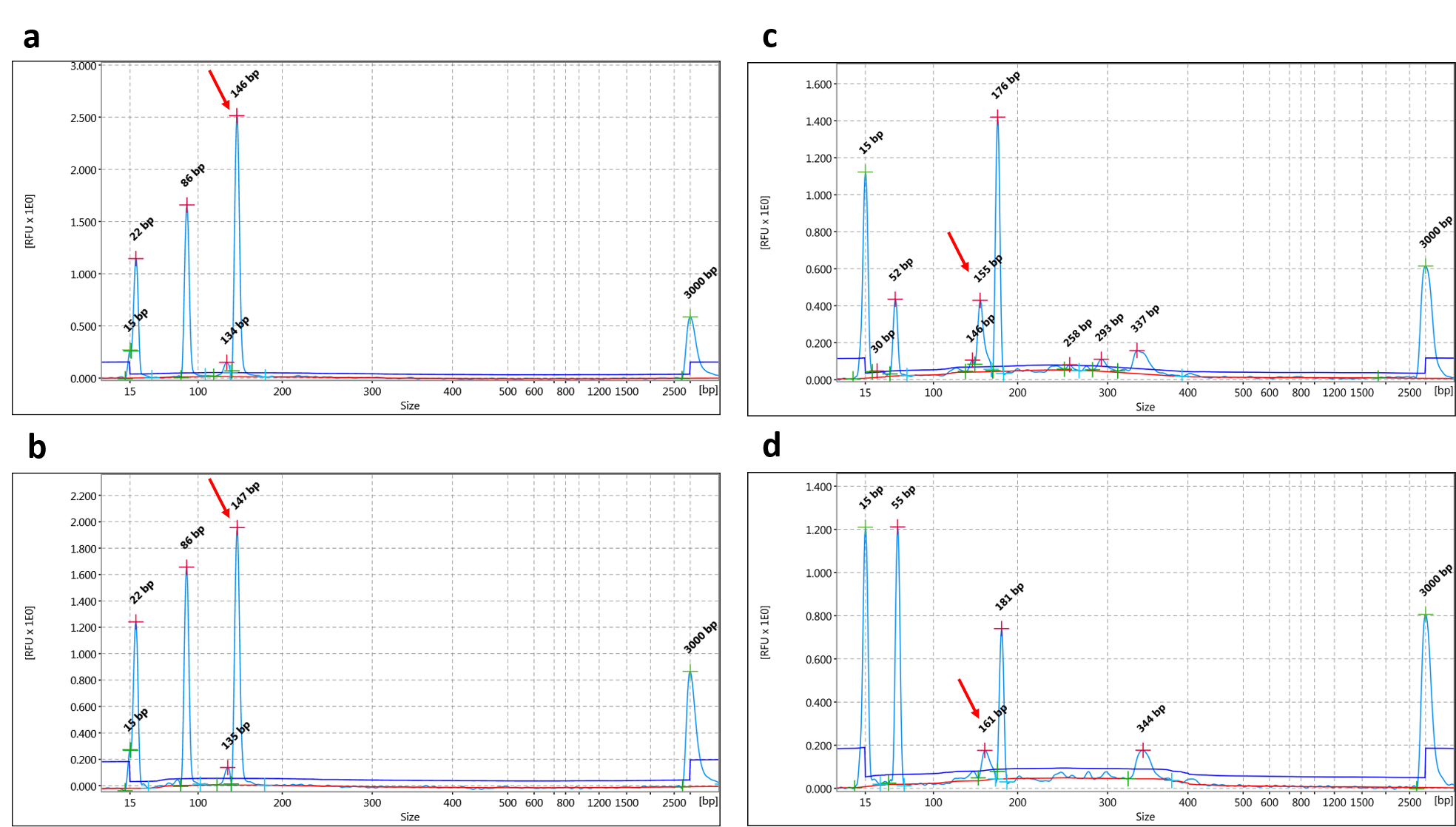


**Figure S35:** Electropherograms showing relative fluorescence units (RFU) as a function of fragment size (in bp) produced by the QIAxcel system for the validation of the putative homozygous deletion in control line M92-220-Long R6-1 at position Chr09:13,714,000-14,037,000 by PCR amplification using diagnostic and control primer pairs on control wild-type DNA (a, c) and M92-220-Long R6-1 DNA (b, d). (a-b) Amplification with control primer pair AM (expected amplicon size of 75 bp) and diagnostic primer pair AT (expected amplicon size of 134 bp). (c-d) Amplification with control primer pair AW (expected amplicon size of 173 bp) and diagnostic primer pair CA (expected amplicon size of 150 bp). None of the primer pairs supported the existence of a deletion at this locus in M92-220-Long R6-1. The red arrows indicate the location (or expected location) of the diagnostic amplicons. The bands observed at 15 bp and 3,000 bp are the size markers.
